# Single-molecule live-cell imaging reveals RecB-dependent function of DNA polymerase IV in double strand break repair

**DOI:** 10.1093/nar/gkaa597

**Published:** 2020-07-20

**Authors:** Sarah S Henrikus, Camille Henry, Amy E McGrath, Slobodan Jergic, John P McDonald, Yvonne Hellmich, Steven T Bruckbauer, Matthew L Ritger, Megan E Cherry, Elizabeth A Wood, Phuong T Pham, Myron F Goodman, Roger Woodgate, Michael M Cox, Antoine M van Oijen, Harshad Ghodke, Andrew Robinson

**Affiliations:** Molecular Horizons Institute and School of Chemistry and Molecular Bioscience, University of Wollongong, Wollongong, NSW 2522, Australia; Illawarra Health and Medical Research Institute, Wollongong, NSW 2522, Australia; Department of Biochemistry, University of Wisconsin-Madison, WI 53706, USA; Molecular Horizons Institute and School of Chemistry and Molecular Bioscience, University of Wollongong, Wollongong, NSW 2522, Australia; Illawarra Health and Medical Research Institute, Wollongong, NSW 2522, Australia; Molecular Horizons Institute and School of Chemistry and Molecular Bioscience, University of Wollongong, Wollongong, NSW 2522, Australia; Illawarra Health and Medical Research Institute, Wollongong, NSW 2522, Australia; Laboratory of Genomic Integrity, National Institute of Child Health and Human Development, National Institutes of Health, Bethesda, MD 20892, USA; Institute of Biochemistry, Goethe Universität, Frankfurt 3MR4+W2, Germany; Department of Biochemistry, University of Wisconsin-Madison, WI 53706, USA; Department of Biochemistry, University of Wisconsin-Madison, WI 53706, USA; Molecular Horizons Institute and School of Chemistry and Molecular Bioscience, University of Wollongong, Wollongong, NSW 2522, Australia; Illawarra Health and Medical Research Institute, Wollongong, NSW 2522, Australia; Department of Biochemistry, University of Wisconsin-Madison, WI 53706, USA; Department of Biological Sciences, University of Southern California, Los Angeles, CA 90089, USA; Departments of Biological Sciences and Chemistry, University of Southern California, Los Angeles, CA 90089, USA; Laboratory of Genomic Integrity, National Institute of Child Health and Human Development, National Institutes of Health, Bethesda, MD 20892, USA; Department of Biochemistry, University of Wisconsin-Madison, WI 53706, USA; Molecular Horizons Institute and School of Chemistry and Molecular Bioscience, University of Wollongong, Wollongong, NSW 2522, Australia; Illawarra Health and Medical Research Institute, Wollongong, NSW 2522, Australia; Molecular Horizons Institute and School of Chemistry and Molecular Bioscience, University of Wollongong, Wollongong, NSW 2522, Australia; Illawarra Health and Medical Research Institute, Wollongong, NSW 2522, Australia; Molecular Horizons Institute and School of Chemistry and Molecular Bioscience, University of Wollongong, Wollongong, NSW 2522, Australia; Illawarra Health and Medical Research Institute, Wollongong, NSW 2522, Australia

## Abstract

Several functions have been proposed for the *Escherichia coli* DNA polymerase IV (pol IV). Although much research has focused on a potential role for pol IV in assisting pol III replisomes in the bypass of lesions, pol IV is rarely found at the replication fork *in vivo*. Pol IV is expressed at increased levels in *E. coli* cells exposed to exogenous DNA damaging agents, including many commonly used antibiotics. Here we present live-cell single-molecule microscopy measurements indicating that double-strand breaks induced by antibiotics strongly stimulate pol IV activity. Exposure to the antibiotics ciprofloxacin and trimethoprim leads to the formation of double strand breaks in *E. coli* cells. RecA and pol IV foci increase after treatment and exhibit strong colocalization. The induction of the SOS response, the appearance of RecA foci, the appearance of pol IV foci and RecA-pol IV colocalization are all dependent on RecB function. The positioning of pol IV foci likely reflects a physical interaction with the RecA* nucleoprotein filaments that has been detected previously *in vitro*. Our observations provide an *in vivo* substantiation of a direct role for pol IV in double strand break repair in cells treated with double strand break-inducing antibiotics.

## INTRODUCTION

DNA polymerase IV (pol IV), encoded by *dinB*, is one of three error-prone DNA polymerases to be produced at increased levels in *Escherichia coli* cells that experience DNA damage (1–3). *In vitro*, pol IV is capable of translesion synthesis (TLS) on a variety of different lesion-containing DNA substrates (4–10). The most commonly discussed function for pol IV within cells is TLS at stalled replication forks, which may help to maintain chromosomal replication in cells experiencing DNA damage (11–15). However, there is significant evidence that pol IV participates in other pathways, including recombinational repair (16–24) and transcription-coupled TLS (25–28). The recruitment of pol IV to the processivity factor β strongly depends on the source of DNA damage (29), indicating that ultimately the type of DNA lesion and changes in metabolism may affect which repair pathway(s) pol IV participates in (10).

We recently completed a single-molecule study of fluorescently tagged pol IV in live *E. coli* cells (30), providing direct evidence that exposure to various DNA-damaging agents (ciprofloxacin, UV light and methyl methanesulfonate [MMS]) leads to the upregulation of pol IV production. Damage-induced upregulation of pol IV is triggered due to SOS induction (31). Following exposure to various DNA-damaging agents, we also detected the binding of individual pol IV molecules to the nucleoid, which presented as punctate foci in fluorescence images (30). Focus formation required the catalytic activity of pol IV. Most interestingly, the study revealed that only 10% of the pol IV foci occurred in the vicinity of replisomes (a colocalization threshold of 218 nm was used; (30)), suggesting that in cells treated with a range of DNA-damaging agents, many of the binding sites for pol IV on the nucleoid are located away from replication forks, potentially at sites of DSB repair (10).

In *E. coli*, DSBs are predominantly repaired through the RecBCD pathway (for a review see (32)). Once formed, DSBs are end-resected by the RecBCD helicase-nuclease complex, generating 3′ single-stranded DNA overhangs. These serve as substrates for loading of the recombinase RecA, generating nucleoprotein filaments denoted RecA*. These filaments facilitate repair of the break via homologous recombination reactions. RecA* filaments are also formed during the repair of single-stranded DNA (ssDNA) gaps via the RecF pathway (33). Several lines of evidence indicate that pol IV can play a role during DSB repair (16–23,34–38). When expressed from a low-copy plasmid, fluorescently labelled pol IV colocalizes with RecA extensively at sites of induced chromosomal DSBs (23). Several genetic studies have demonstrated that the gene encoding pol IV, *dinB*, is required for both induced and spontaneous error-prone DSB repair (18,20,34–37). *In vitro* studies have also linked pol IV to RecA-mediated recombination, showing that pol IV efficiently utilizes model D-loop recombination intermediates (21,22). Additionally, pol IV physically interacts with the RecA protein *in vitro* (8,38). The results of a recent study indicate that pol IV-dependent error-prone break repair is a major pathway underlying increased rates of mutagenesis in cells treated with ciprofloxacin at sub-inhibitory concentrations (39). The study revealed that ciprofloxacin-induced mutagenesis is concentrated within a distinct sub-population of cells that express high levels of RpoS, brought on by elevated production of reactive oxygen species (ROS).

Here, we extended our single-molecule fluorescence microscopy study (30) to investigate whether the formation and processing of DSBs influence the regulation of pol IV expression levels and the formation of pol IV foci on the nucleoid. We chose to investigate two antibiotics, ciprofloxacin and trimethoprim, that each induce DSB formation, albeit via entirely different mechanisms. Ciprofloxacin is an inhibitor of the type II topoisomerases DNA gyrase and topoisomerase IV (40). Ciprofloxacin, like other (fluoro)quinolones, traps a DNA-cleaved intermediate state of the topoisomerase cycle in a quinolone-stabilized cleavage complex, which inhibits replication fork progression and induces DSB formation through multiple mechanisms (41,42). Trimethoprim induces DSBs through mechanisms that involve ROS (43,44). By inhibiting folate biosynthesis, trimethoprim treatment leads to metabolic disturbances, including depletion of the nucleotide pool and kills cells through a pathway that is related, but not identical to, thymineless death (44,45). There are two models for the formation of DSBs in cells treated with trimethoprim. First, Giroux *et al.* demonstrated recently that the killing of cells by trimethoprim is linked to maladaptive DNA repair. In this model, base-excision repair enzymes, acting at sites of oxidative damage on the chromosome, inadvertently create DSBs when they attempt to repair lesions that are close to each other (44). Second, a recent study of the thymineless death mechanism (43) revealed that thymine depletion induces the formation of ssDNA gaps behind replication forks, which are subsequently converted to DSBs by ROS. Given that thymineless death and trimethoprim-induced death share common features (44,45), it is plausible that a similar pathway operates in trimethoprim-treated cells.

For both ciprofloxacin and trimethoprim treatments, we show that levels of SOS-associated pol IV expression and the formation of pol IV foci correlate with the number of DSBs detected using a fluorescent MuGam probe. We found that SOS induction and pol IV focus formation are highly dependent on the RecBCD pathway. The presence of RecA*-like structures in cells is sufficient to promote the binding of pol IV to the nucleoid, even in the absence of exogenous DNA damage. The observations strongly suggest that in cells treated with the DSB-promoting antibiotics ciprofloxacin and trimethoprim, the primary substrates for pol IV in cells are DSB repair intermediates.

## MATERIALS AND METHODS

### Strain construction

Strains used in this study are summarized in Table 1. Plasmids are summarized in Table 2.

**Table 1.** Strains used in this study

Strain	Relevant genotype	Parent strain	Source/technique
MG1655	*dinB^+^ dnaX^+^ recB^+^ lexA^+^*		Published (92)
EAW102	Δ*recB*::Kan^R^	MG1655	Lambda Red recombination
HG356	Δ*recB*::FRT	MG1655	EAW102
DE407	*lexA3*(Ind^−^)	DE192	Published (48)
RW1568	*lexA3*(Ind^−^)	MG1655	Transduction of MG1655 with P1 grown on DE407
EAW114	Δ*recO*::Kan^R^	MG1655	Lambda Red recombination
EAW629	Δ*recF*::Kan^R^	MG1655	Lambda Red recombination
EAW669	Δ*recR*::Kan^R^	MG1655	Lambda Red recombination
EAW671	Δ*recF*::FRT Δ*recR*::Kan^R^	EAW669	Transduction of EAW669 with P1 grown on EAW629
EAW693	Δ*recF*::FRT Δ*recR*::FRT Δ*recO*::Kan^R^	EAW671	Transduction of EAW671 with P1 grown on EAW114
EAW287	*recA*(E38K) *sulA*^−^::FRT	MG1655	Published (50)
EAW18	Δ*dinB*::Kan^R^	MG1655	Published (30)
EAW642	*dnaX-mKate2*::Kan^R^	MG1655	Published (30)
EAW633	*dinB-YPet*::Kan^R^	MG1655	Published (30)
EAW643	*dinB-YPet*::FRT *dnaX-mKate2*::Kan^R^	EAW633	Published (30)
JJC5945	*dnaX-YPet*::Kan^R^	MG1655	Published (50)
EAW191	*umuC-mKate2*::Kan^R^	MG1655	Published (50)
EAW282	*umuC-mKate2*::FRT *dnaX-YPet*::Kan^R^	JJC5945	Published (50)
RW120	*recA* ^+^ *sulA* ^−^ *lexA* ^+^ Δ*umuDC*::Cm^R^	RW118	Published (49)
RW880	Δ*umuDC*::Cm^R^	MG1655	Transduction of MG1655 with P1 grown on RW120 (49)
EAW13	*sulA* ^−^::Kan^R^	MG1655	Published (50)
EAW1134	*dinB-YPet*::FRT *dnaX-mKate2*::FRT *sulA^−^*::Kan^R^	EAW643	Transduction of EAW643 with P1 grown on EAW13 (46)
DE406	*lexA*(Def)::Cm^R^	DE192	Published (51)
EAW1141	*dinB-YPet*::FRT *dnaX-mKate2*::FRT *sulA*^−^::FRT *lexA51*(Def)::Cm^R^	EAW1134	Transduction of EAW1134 with P1 grown on DE406 (51)
EAW1144	*dinB-YPet*::FRT *dnaX-mKate2*::FRT *sulA*^−^::FRT *lexA51*(Def)::Cm^R^ Δ*recB*::Kan^R^	EAW1141	Transduction of EAW1141 with P1 grown on EAW102
RW244	*recA730 srlD300*::Tn*10*	MG1655	Published (52)
RW1594	*dinB-YPet dnaX-mKate2 sulA* ^−^::Kan^R^*lexA51*(Def)::Cm^R^	RW1588	Published (30)
RW1598	*dinB-YPet dnaX-mKate2 sulA*::kan^R^*lexA*(Def)::Cm^R^*recA*(E38K) *srlD300*::Tn*10*	RW1594	Transduction of RW1594 with P1 grown on RW244
EAW830	*dinB*(D103N)*-YPet*	MG1655	Published (30)
AR023	*recA730 sulA* ^−^::FRT *dinB*(D103N)*-YPet*	EAW287	Transduction of EAW287 with P1 grown on EAW830
EAW282 *sulA*^−^	*umuC-mKate2*::FRT *dnaX-YPet*::FRT *sulA*^−^::Kan^R^	EAW282	Transduction of EAW282 with P1 grown on EAW13
RW1286	*umuC-mKate2*::FRT *dnaX-YPet*::FRT *sulA*^−^::Kan^R^*lexA51*(Def)::Cm^R^	EAW282 *sulA*^−^	Transduction of EAW282 *sulA*^−^ with P1 grown on DE406
RW546	*recA+ sulA* ^−^ *lexA51*(Def) Δ*umuDC*::Cm^R^	RW542	Published (93)

**Table 2.** Plasmids used in this study

Plasmid	Description	Source
pEAW1159	pBAD derivative expressing *MuGam*	This study
pEAW1162	pBAD derivative expressing *MuGam-PAmCherry* fusion	This study
pSTB-*sodA-gfp*	pQBI63 derivative expressing superfolder GFP from the SoxS/Fur-regulated *sodA* promoter	This study
pCJH0008	pQBI63 derivative expressing superfolder GFP from the OxyR-regulated *ahpC* promoter	This study
pCJH0009	pQBI63 derivative expressing superfolder GFP from the Fur-regulated *fepD* promoter	This study
pUA139-P*_sulA_*-*gfp*	pUA139 derivative expressing GFP from the SOS-regulated *sulA* promoter	(80)
pBAD-PAmCherry-mcI	pBAD derivative expressing *PAmCherry* fusion of mCI, a probe derived from the cI protein from bacteriophage λ	(57)
pLH29	Plasmid expressing FLP recombinase	(46)
pRW154	Plasmid expressing UmuD and UmuC	(49)

EAW102 is *E. coli* K-12 MG1655 Δ*recB* and was constructed using *λ*_RED_ recombination. The kanamycin resistance marker in EAW102 was removed via FLP-FRT recombination (46) using the plasmid pLH29 to obtain the kanamycin-sensitive variant HG356.

EAW693 is *E. coli* K-12 MG1655 Δ*recF* Δ*recO* Δ*recR* and was constructed following three *λ*_RED_ recombinations. Three P1 phage lysates were raised, using strains constructed in a previous study (47): (i) EAW114 (Δ*recO*::Kan^R^), (ii) EAW629 (Δ*recF*::Kan^R^) and (iii) EAW669 (Δ*recR*::Kan^R^). The kanamycin resistance was cured after each transduction step using FLP-FRT recombination (46) to obtain the kanamycin-sensitive variants. Finally, a kanamycin-sensitive variant of EAW693 (Δ*recF*::FRT Δ*recO*::FRT Δ*recR*::FRT) was produced.

The SOS-uninducible strain RW1568 was made by P1 transduction of *lexA3*(ind^−^) *malB*::*Tn9* from DE407 (48) into MG1655, selecting for selecting for chloramphenicol resistance and then screening for *lexA3*(ind^−^) associated UV sensitivity. For testing sensitivites to DNA damaging agents, the Δ*umuDC* strain RW880 was constructed by P1 transduction of Δ*umuDC595*::*cat* from RW120 (49) into MG1655, selecting for chloramphenicol resistance.

EAW1144 is *E. coli* K-12 MG1655 *dinB-YPet dnaX-mKate2 sulA*^−^*lexA51*(Def) Δ*recB* and was constructed in three steps. First, *sulA*^−^ FRT-Kan-FRT was P1 transduced into EAW643 (Kan^S^) using a P1 lysate grown on EAW13 (50) to obtain the strain EAW1134. Second, Kan cassette was removed using FLP recombinase, which was expressed from pLH29 (46). Third, *lexA51*(Def) *malB*::Tn*9* was transduced into EAW1134 using a P1 lysate grown on DE406 (51) to obtain the strain EAW1141. A Δ*recB* derivative, EAW1143, was prepared by transducing Δ*recB* FRT-KanR-FRT into EAW1141 using P1 lysate grown on EAW102. All mutations introduced were confirmed by polymerase chain reaction (PCR).

RW1598 was made by P1 transduction of *recA730 srlD300*::Tn*10* from RW244 (52) into RW1594, selecting for TetR. Colonies were then screened for constitutive UmuD cleavage using Western blotting, using materials and methods described previously (53). *recA* and *srlD* are approximately 90% linked.

AR023 was made by P1 transduction of *dinB*(D103N)-YPet::kan^R^ from EAW830 (30) into EAW287 ((50), *recA*[E38K]::FRT[kan^S^]), selecting for kanamycin resistance.

RW1286 is *E. coli* MG1655 *umuC-mKate2 dnaX-YPet sulA*^−^::kan^R^*lexA51*(Def)::Cm^R^ and was made in two steps. First, the wild-type *sulA* gene of EAW282 (50) was replaced with *sulA*^−^::kan by P1 transduction from EAW13 (50), to create EAW282 *sulA*^−^. Next, the *lexA51*(Def) *malB*::Tn*9* allele was transferred from DE406 (51) into EAW282 *sulA*^−^ by P1 transduction, selecting for chloramphenicol resistance. To confirm the presence of the *lexA*(Def) genotype, colonies were then screened for high levels of RecA expression by Western blotting with anti-RecA antibodies (54).

The pBAD-*MuGam* plasmid (pEAW1159) was constructed using a PCR-amplified *muGam* gene fragment (us = GGATATCCATATGGCTAAACCAGCAAAACGTA) consisting of a NdeI site and the beginning of the *muGam* gene, and MuGam (ds = GCGAATTCTTAAATACCGGCTTCCTGTTCA consisting of an EcoRI site and the end of the *muGam* gene) from EAW727 (MG1655 Founder (55) e14 with chromosomal *muGam-gfp* in the *att*Tn*7* site). EAW727 was constructed by transducing *muGam-gfp* into Founder e14 using a P1 lysate grown on SMR14350 (56). The PCR product was digested with NdeI and EcoRI and inserted into pBAD NdeI which was cut with the same enzymes. pBAD NdeI is pBAD/Myc-HisA (Invitrogen) that has been mutated to add a NdeI site in place of the original NcoI site. All other NdeI sites were filled in before the mutagenesis. The resulting plasmid was directly sequenced to confirm presence of wild-type *muGam* gene

The pBAD-*MuGam-PAmCherry* vector (pEAW1162) was constructed by using two PCR fragments: (i) NdeI-MuGam-linker-EcoRI generated from pEAW1159 using the following PCR primers: MuGam us = GGATATCCATATGGCTAAACCAGCAAAACGTA consisting of a NdeI site and the beginning of the *muGam* gene, and MuGam ds no stop link = GGATATCGAATTCGCCAGAACCAGCAGCGGAGCCAGCGGAAATACCGGCTTCCTGTTC AAATG consisting of an EcoRI site, an 11aa linker, and the end of the *muGam* gene without a stop codon. The PCR product was digested with NdeI and EcoRI. (ii) EcoRI-PAmCherry- HindIII generated from pBAD-*PAmCherry-mCI* (57) using the following PCR primers PAmCherry usEco = GGATATCGAATTCATGGTGAGCAAGGGCGAGGAG consisting of an EcoRI site and the beginning of mCherry, and PAmCherry dsHind = GGATATCAAGCTTTTACTTGTACAGCTCGTCCAT consisting of a HindIII site and the end of the *mCherry* gene. The PCR product was digested with EcoRI and HindIII. Both PCR products were ligated to pBAD NdeI that had been digested with NdeI and HindIII. The resulting plasmid was directly sequenced to confirm the presence of *muGam-PAmCherry*.

### ROS reporter fusions construction

Three promoters of genes regulated by changes in ROS or iron levels were cloned upstream of the *sf-gfp* gene (58) in a pQBI63 plasmid (Qbiogene). Briefly, upstream regions of *sodA* gene (consisting of the 284 nt intergenic region of *rhaT* and *sodA*) regulated by *soxS* and Fur (59,60), or *ahpC* gene (−372 to −1 nt of ATG) regulated by OxyR (59,61–62), or *fepD* gene (−170 to −1 nt of ATG) regulated by Fur (63), were amplified and cloned into the pQBI63 plasmid using BglII/NheI restriction enzymes to generate respectively pSTB-*sodA- gfp*, pCJH0008 and pCJH0009. All constructions were confirmed by sequencing.

### DNA damaging agent sensitivity assay

Cells were grown overnight in EZ rich defined medium (Teknova) that contained 0.2% w/v glucose medium (EZ glucose) at 37°C. The next day, a dilution 1/1000 of each culture was grown in EZ glucose (at 37°C, 150 rpm) until reaching mid log phase (OD_600_ = 0.3). Six aliquots of 300 μl of each culture were transferred into 24-well microplates. The first aliquot was used as control of no treatment, 2% dimethyl sulfoxide (DMSO, 282 mM, 0.2 × MIC (43)), 30 ng/ml ciprofloxacin, 30 ng/ml ciprofloxacin + 2% DMSO, 1 μg/ml trimethoprim or 1 μg/ml trimethoprim + 2% DMSO were added in the others. Samples of 150 μl were taken at 0 and 60 min; samples at 0 h were taken just before treatment. Each sample was serial diluted in PBS by factor 10 down to 10^−6^ and dilutions 10^−1^ to 10^−6^ were spotted on fresh LB plates (Difco brand). Plates were incubated overnight at 37°C in the dark.

### Survival assay following MuGam-PAmCherry expression

To test the effect of MuGam-PAmCherry expression levels on lethality following ciprofloxacin and trimethoprim exposure, seven cell cultures were set up, expressing different levels of MuGam-PAmCherry from a pBAD plasmid. Cells cultures 1–7 (each 1 ml) were grown in EZ rich defined medium (Teknova) that contained 0.2% w/v glycerol medium (EZ glycerol) in the presence of ampicillin (100 μg/ml) and different concentrations of L-arabinose (0, 0.001, 0.003, 0.01, 0.03, 0.1%). Cell culture 8 (1 ml) was grown EZ glucose medium in the presence of ampicillin (100 μg/ml) overnight at 37°C, 950 rpm. The next day, a 1/1000 dilution of each culture (final volume of 1.5 ml) was grown under the same conditions as over-night growth for 3 h. Each culture was split in three and no drug, 30 ng/ml ciprofloxacin or 1 μg/ml trimethoprim was added. These cultures were grown (at 37°C, 950 rpm) for 2 h. Then, cultures were spun down (5 min; 5000 × *g*) and cell pellets were resuspended in 0.5 ml corresponding EZ medium. Centrifugation and resuspension was carried out a total of three times. Each cell culture was serial diluted in PBS by factor ten down to 10^−5^ and dilutions 10^−1^ to 10^−5^ were spotted on fresh LB plates containing 100 μg/ml ampicillin (Difco brand). Plates were incubated overnight at 37°C in the dark. For each condition, biological triplicates were performed. From these experiments, an L-arabinose concentration of 0.003% was chosen for fluorescence microscopy experiments because this L-arabinose concentration showed no drastic decrease in survival compared to the sample grown in the presence of glucose.

### Plate reader assay

Cells were grown in EZ glucose medium overnight at 37°C. The next day, a dilution 1:100 of each culture was grown in EZ glucose (at 37°C, 950 rpm) for 3 h. These cultures were diluted to 1/200. Then, 10 μl of these diluted cultures were added to a total volume of 200 μl medium in each well of a 96-well plate. These 200 μl of media contained antibiotic, or hydrogen peroxide, and/or ROS mitigators (final concentration: 5, 10, 20 and 40 ng/ml ± 2% DMSO or ± 0.35 mM 2,2′-bipyridyl [BiP]; 0.1, 0.3, 1 and 3 μg/ml ± 2% DMSO or ± 0.35 mM BiP; 30, 100, 300 and 500 mM hydrogen peroxide [H_2_O_2_] ± 2% DMSO). For experiments with antibiotics and/or ROS mitigators, antibiotics and/or ROS mitigators were added just prior to the cells being added. For experiments with hydrogen peroxide, hydrogen peroxide was added immediately after the cells were added. For each well, absorbance (OD_600_) and fluorescence (*λ*_excitation_ = 470 ± 15 nm, *λ*_emission_ = 515 ± 20 nm) were measured every 30 min over 17 h. For cells carrying pUA139-P*_sulA_*-*gfp*, experiments were carried out in 96-well plates from Nalge Nunc International (no. 265301). For cells carrying pSTB-*sodA-gfp*, pCJH0008 or pCJH0009, experiments were carried out in 96-well plates from Thermo Scientific (no. 165305). The experiments were carried out using the CLARIOstar plate reader (BMG Labtech; settings: orbital reading 4 mm (for 96-well plates from Nalge Nunc International) or 2 mm (for 96-well plates from Thermo Scientific), orbital shaking at 200 rpm, at 37 °C).

Cell cultures were also serial diluted and plated on LB agar plates in order to calculate the number of cells added to each well. To each well, when adding wild-type or Δ*recFOR* cells, 10^5^–10^6^ cells were added at the beginning of the experiment. For experiments when adding Δ*recB* cells, 10^5^ cells were added at the beginning of the experiment.

### Fluorescence microscopy

Measurements were recorded on three microscope setups. Most of the imaging was conducted on an inverted microscope (IX-81, Olympus with a 1.49 NA 100× objective) in an epifluorescence configuration (50). Continuous excitation was provided using semidiode lasers (Sapphire LP, Coherent) of the wavelength 514 nm (150 mW max. output) and 568 nm (200 mW max. output). τ-mKate2 in EAW643 and UmuC-mKate2 in EAW282 were imaged using yellow excitation light (*λ* = 568 nm) at high intensity (2750 W cm^−2^), collecting emitted light between 610–680 nm (ET 645/75m filter, Chroma) on a 512 × 512 pixel EM-CCD camera (C9100-13, Hamamatsu). Images of UmuC-mKate2 in RW1286 were recorded at 275 W cm^-2^. For DinB-YPet imaging of EAW643 time-lapse experiments, we used green excitation (*λ* = 514 nm) at 160 W cm^−2^ collecting light emitted between 525–555 nm (ET540/30m filter, Chroma). For DinB-YPet imaging of RW1594, RW1598 and EAW643 burst acquisitions, cells were imaged at 51 W cm^−2^. τ-YPet imaging (EAW282, RW1286) was performed at 51 Wcm^−2^. Cells carrying the SOS reporter plasmid pUA139-P*_sulA_*-*gfp* were imaged at 16 W cm^−2^. Data comparing SOS induction in wild-type, Δ*recB* and *lexA*(Ind^−^) backgrounds, as well as data comparing DinB-YPet and DinB(D103N)-YPet foci in the *recA*(E38K) background, were recorded on a Nikon Eclipse Ti2 microscope equipped with a 1.49 NA 100 × objective, using the same excitation optics and camera described above.

For experiments involving MuGam-PAmCherry, imaging was conducted on an inverted microscope (Nikon Eclipse-Ti), equipped with a 1.49 NA 100 × objective and a 512 × 512 pixel Photometrics Evolve CCD camera (Photometrics, AZ, US). NIS-Elements equipped with JOBS module was used to operate the microscope (Nikon, Japan). Continuous excitation is provided using semidiode lasers of the wavelength 405 nm (OBIS, Coherent, 200 mW max. output), 514 nm (Sapphire LP, Coherent, 150 mW max. output) and 568 nm (Sapphire LP, Coherent, 200 mW max. output). MuGam-PAmCherry was imaged by simultaneous illumination with the activation laser 405 nm (1–5 W cm^−2^) and 568 nm readout laser (540 W cm^−2^), a PALM (photoactivation localization microscopy) acquisition protocol, collecting emitted light from 590 nm (ET590LP, Chroma). DinB-YPet was imaged using green excitation (*λ* = 514 nm) at lower power (∼2200 W cm^−2^), collecting light emitted between 535–550 nm (ET535/30m filter, Chroma). NIS-Elements equipped with JOBS module was used to operate the microscope (Nikon, Japan).

Two-color time-lapse movies were recorded to visualize if DinB-YPet foci overlap with τ-mKate2 foci (EAW643). Sets of three images were recorded (bright-field [34 ms exposure], mKate2 fluorescence [100 ms exposure], YPet fluorescence [50 ms exposure]) at an interval of 10 min for 3 h. To measure colocalization between UmuC-mKate2 with the replisome marker τ-YPet (EAW282), we recorded time-lapse movies at the same intervals but different exposures for the replisome marker (bright-field [34 ms exposure], mKate2 fluorescence [100 ms exposure], YPet fluorescence [500 ms exposure]).

Burst acquisitions of DinB-YPet (movies of 300 × 50 ms frames taken every 100 ms light at 514 nm) were collected, subsequently to each burst acquisition, an image of τ-mKate2 (568 nm) was taken (imaging sequence for RW1594) and a bright-field image (34 ms exposure). With this imaging sequence, we determined the number of DinB-YPet foci per cell in Figures 4 and 5. RW1286 was imaged similarly (Figure 6); we recorded burst acquisitions of UmuC-mKate2 (568 nm) followed by a snapshot of τ-YPet (514 nm). All images were analyzed with ImageJ (64).

The MuGam-PAmCherry imaging acquisition was recorded as a set of two acquisitions (bright-field image [100 ms exposure], PAmCherry fluorescence [simultaneous illumination with the activation laser 405 and 568 nm readout laser for 200 frames each with 100 ms exposure]). This protocol was only executed once for a field-of-view to minimize laser damage. Consequently, before and after antibiotic treatment shows a new set of cells. Images taken after antibiotic addition were recorded following 2 h of antibiotic treatment.

Time-sampling of DinB-YPet and PAmCherry-mCI expressing cells were performed as follows: sets of three acquisitions were recorded (bright-field [100 ms exposure], YPet fluorescence [50 ms exposure]; PAmCherry fluorescence [simultaneous illumination with the activation laser 405 and 568 nm readout laser for 200 frames each with 100 ms exposure]). This protocol was only executed once for a field-of-view to minimize laser damage. Consequently, each time point shows a new set of cells. The experiment was conducted over 3 h, an image was taken every 5 min.

### Flow cell designs

All imaging experiments were carried out in home-built quartz-based flow cells. These flow cells were assembled from a no. 1.5 coverslip (Marienfeld, REF 0102222, for imaging on IX-81, Olympus) or (Marienfeld, REF 0107222, for imaging on Nikon Eclipse-Ti), a quartz top piece (45 × 20 × 1 mm) and PE-60 tubing (Instech Laboratories, Inc.). Prior to flow-cell assembly, coverslips were silanized with (3-aminopropyl)triethoxysilane (APTES, from Alfa Aeser). First, coverslips were sonicated for 30 min in a 5M KOH solution to clean and activate the surface. The cleaned coverslips were rinsed thoroughly with MilliQ water and then treated with a 5% (v/v) solution of APTES in MilliQ water. The coverslips were subsequently rinsed with ethanol and sonicated in ethanol for 20 seconds. Afterward, the coverslips were rinsed with MilliQ water and dried in a jet of N_2_. Silanized slides were stored under vacuum prior to use.

To assemble each flow cell, polyethylene tubing (BTPE-60, Instech Laboratories, Inc.) was glued (BONDiT B-482, Reltek LLC) into two holes that were drilled into a quartz piece. After the glue solidified overnight, double-sided adhesive tape was stuck on two opposite sides of the quartz piece to create a channel. Then, the quartz piece was stuck to an APTES-treated coverslip. The edges were sealed with epoxy glue (5 Minute Epoxy, PARFIX). Each flow cell was stored in a desiccator under mild vacuum while the glue dried. Typical channel dimensions were 45 × 5 × 0.1 mm (length × width × height).

### Preparation of cell cultures for microscopy

To minimize background fluorescence in cells, strains were first cultured overnight in EZ rich defined medium (Teknova), then sub-cultured by diluting the overnight culture 1:1000 in fresh EZ rich defined medium and incubating for ∼3 h prior to imaging. With the exception of cells carrying the MuGam-PAmCherry plasmid pEAW1162, all strains were grown at 37°C in EZ glucose. All strains containing a Kan^R^ cassette were grown in the presence of kanamycin (20 μg/ml). Cells carrying pEAW1162 were grown at 37°C in EZ glycerol containing 0.001% L-arabinose and ampicillin (100 μg/ml). Cultures used for imaging under ROS-mitigating conditions were grown in the presence of the particular mitigator used for the experiment during the sub-culturing step; DMSO (2% v/v, 282 mM, 0.2 × MIC (43)) or BiP (0.35 mM, 0.5 × MIC (43)).

### Imaging in flow cells

Cells were loaded into flow cells, allowed a few minutes to associate with the APTES surface, then loosely associated cells were removed by pulling through fresh medium. The experiment was then initiated by adding either an antibiotic alone or in combination with DMSO to the medium (30 ng/ ml ciprofloxacin, 30 ng/ ml ciprofloxacin with 2% (v/v) DMSO, 1 μg/ml trimethoprim, 1 μg/ml trimethoprim with 2% (v/v) DMSO or 1 μg/ml trimethoprim with 0.35 mM BiP). Throughout the experiment, medium was pulled through the flow cell using a syringe pump, at a rate of 50 μl/min. For each condition, triplicate measurements were recorded.

### Analysis of cell filamentation, concentrations, SOS induction level and number of foci

We selected single cells to obtain information about SOS induction, DinB and UmuC levels upon UV irradiation (>100 cells for every time point). MicrobeTracker 0.937 (65), a MATLAB script, was used to create cell outlines as regions of interest (ROI). We manually curated cell outlines designated by MicrobeTracker at *t* = 0 min (time point of antibiotic addition) and at 30 min time intervals until 180 min. By curating cell outlines manually, we ensure accuracy and purely select non-overlapping, in-focus cells for analysis. These ROI were imported in ImageJ 1.50i. The cell outlines were then used to measure mean cell intensities, cell lengths and the number of foci per cell. Parameters describing foci (number, positions and intensities) were obtained using a Peak Fitter plug-in, described previously (30,50). Prior to determining DinB-YPet foci UmuC-mKate2 per cell from burst acquisition movies in *lexA*(Def), average projections in time were curated from frame 1 to 101 (10 × 100 ms = 1 s). Prior to determining MuGam-PAmCherry or PAmCherry-mCI foci per cell from burst acquisition movies, maximum projections in time were curated over the entire movie, capturing all binding events of MuGam-PAmCherry.

Using information of mean cell brightness derived from DinB-YPet expressing cells, we also calculated DinB-YPet concentrations of cells grown in the absence or presence of antibiotic. In a previous study (30), we calculated the DinB-YPet concentration which correlates with a certain mean cell brightness (in the absence of ciprofloxacin: 6 ± 1 nm [SEM]; 180 min after ciprofloxacin treatment: 34 ± 3 nM [SEM]). We utilized these values to calculate the DinB-YPet concentration for ciprofloxacin ± DMSO or trimethoprim ± DMSO treated cells.

### Analysis of colocalization events

Foci were classed as colocalized if their centroid positions (determined using our peak fitter tool) fell within 2.18 px (218 nm) of each other. When treating with ciprofloxacin, we determined that for DinB-YPet–τ-mKate2 localization the background of DinB foci expected to colocalize with replisomes purely by chance is ∼4% at 180 min. This was calculated by taking the area of each cell occupied by replisome foci (including the colocalization search radius) and dividing by the total area of the cell. The value of 4% corresponds to the mean of measurements made over 121 cells. Since the foci density of replisomes stays fairly constant following ciprofloxacin treatment, the chance colocalization of DinB-YPet foci with τ-mKate2 is ∼4% during the experiment (30). Chance colocalization of τ-mKate2 with DinB-YPet is however not constant over time because most cells contain no pol IV foci in the absence of any DNA damage. Chance colocalization is close to zero at 0 min; at 60 min, chance colocalization is ∼5%; at 120 min, chance colocalization is ∼3%. Moreover, chance colocalization of τ-mKate2 with DinB-YPet is overall reduced under ROS-mitigating conditions due to a reduced number of foci per cell (chance colocalization close to zero at 0 min; at 120 min, ∼2%). Chance colocalization of τ-mKate2 with DinB-YPet in trimethoprim-treated cells amounts to ∼1% from 60–90 min (close to zero before 60 min). Under ROS-mitigating conditions, chance colocalization is always close to zero because both the mean cell size and the number of pol IV foci per cell do not increase post treatment (see Supplementary Materials).

The chance colocalization of UmuC-mKate2 with τ-YPet is similar to the chance colocalization of DinB-YPet with τ-mKate2 (chance colocalization: ∼4%). The expected colocalization of τ-YPet with UmuC-mKate2 by background is close to zero until 90 min as UmuC-mKate2 is neither upregulated nor released from the membrane (see ‘Results’ section). Chance colocalization is ∼3% at 180 min after ciprofloxacin treatment and ∼2% after the combinational treatment of ciprofloxacin/DMSO.

### Proteins

The wild-type *E. coli* RecA protein was purified using strains and protocols described by Craig and Roberts (66). The RecA concentration was determined using the extinction coefficient ϵ_280_ = 2.23 × 10^4^ M^−1^ cm^−1^ (66).

The *E. coli* RecA(E38K) protein was purified as previously described (67) with the following modifications. After washing the protein pellet with R buffer plus 2.1 M ammonium sulfate, the pellet was resuspended in R buffer plus 1 M ammonium sulfate. The sample was loaded onto a butyl-Sepharose column and washed with 1.5 column volumes of R buffer plus 1 M ammonium sulfate. It was then eluted with a linear gradient from R buffer plus 1 M ammonium sulfate to R buffer, carried out over five column volumes. Peak fractions were identified by sodium dodecyl sulphate-polyacrylamide gelelectrophoresis (SDS-PAGE) analysis and pooled. The protein was loaded onto a hydroxyapatite column as done previously, but with the linear gradient from 10–500 mM P buffer. The fractions were dialyzed against R buffer plus 50 mM KCL and 1 mM dithiothreitol three times. The fractions were loaded onto a Source 15S column and washed with R buffer plus 50 mM KCl and 1 mM dithiothreitol until the UV trace receded from peak. Next, the pool was loaded onto a Source 15Q column and eluted with a linear gradient from 0.05–1 M KCl over 25 column volumes. Peak fractions were identified as above and pooled. A DEAE-Sepharose column was not used. Protein in this pool was precipitated by the addition of equal volume of 90% saturated ammonium sulfate. The precipitate was stirred and then spun down at 13 000 rpm for 30 min. The pellet was resuspended in R buffer plus 1 M ammonium sulfate, stirred for an hour, and then spun down again. This protein was loaded onto a butyl-Sepharose column and eluted in a gradient from R buffer and 1 M ammonium sulfate to R buffer. The fractions were identified, pooled, and concentrated using GE Vivispin 20 10K MWCO centrifuge filter concentrating units. The protein was flash frozen in liquid nitrogen and stored at −80°C. The concentration was determined as above. No exonuclease or other endonuclease activities were detected.

Pol IV (*dinB*) coding sequence was cloned into NcoI and BamHI sites of pET16b to generate a native pol IV expression construct. *E. coli* strain Tuner/pLysS (Novagen) carrying the expression construct was grown in LB medium supplemented with 20 μg/ml chloramphenicol and 100 μg ml–1 ampicillin. Expression of pol IV was induced by adding IPTG to 1 mM and growing for 3–4 h at 30°C. Collected cells (∼20 g) were resuspended in 50 mL of lysis buffer (50 mM Tris–HCl, pH 7.5, 1 M NaCl, 10% sucrose, 2 mM dithiothreitol, 1 mM ethylenediaminetetraacetic acid (EDTA) and protease inhibitor cocktail). Cells were lysed by lysozyme (2 mg/ml) and the clarified extract was collected following centrifugation at 15 000 × *g* for 30 min. Pol IV was then precipitated by ammonium sulfate added to 30% saturation and stirring for 10 min. The precipitate was subjected to gel-filtration in GF-buffer (20 mM Tris–HCl, pH 7.5, 1 M NaCl, 0.1 mM EDTA, 1 mM dithiothreitol) using a GE Healthcare Superdex-75 XK-26/60 gel filtration column. Pol IV fractions were pooled, dialyzed overnight in PC-buffer (20 mM Tris–HCl, pH 7.5, 0.1 mM EDTA 1 mM dithiothreitol, 10% glycerol), containing 200 mM NaCl and then subjected to phosphocellulose chromatography (P-11, Whatman). After washing extensively with PC-buffer + 200 mM NaCl, pol IV was eluted with a linear gradient of 200–500 mM NaCl. Fractions containing native pol IV (>99% pure) were pooled and stored at −70°C.

### Surface plasmon resonance (SPR) experiments

Surface plasmon resonance (SPR) experiments were conducted on BIAcore T200 instrument (GE Healthcare) using streptavidin (SA) coated sensor chips, probing the formation of RecA structures (assembled from RecA[E38K]) on ssDNA and dsDNA. Experiments were carried out at 20°C at a flow rate of 5 μl min^−1^. As described previously (57), SA chips were activated and stabilized, single-stranded biotinylated 71-mer poly-dT oligonucleotide bio-(dT)_71_ was immobilized, followed by RecA(E38K) filament assembly. RecA(E38K) filaments were assembled on bio-(dT)_71_ by injecting 1 μM RecA(E38K) in SPR^RecA(E38K)^ buffer (20mM Tris–HCl, pH 8.0, 10 mM KCl, 10 mM MgCl2, 0.005% surfactant P20 and 0.5 mM dithiothreitol) supplemented with 1 mM adenosine 5′-(γ-thio) triphosphate (ATPγS) at 10 μl min^−1^ for 400 s. Similarly, biotinylated dsDNA was immobilized (as previously described (57)), followed by RecA(E38K) filament assembly. RecA(E38K) filaments were assembled on dsDNA (sequence: 5′-TCC TTT CGT CTT CAA AGT TCT AGA CTC GAG GAA TTC TAA AGA TCT TTG ACA GCT AGC CAG-3′, 5′ end is biotinylated) by injecting 1 μM RecA(E38K) in SPR^RecA(E38K)^ buffer (20 mM Tris–HCl, pH 8.0, 10 mM KCl, 10 mM MgCl2, 0.005% surfactant P20 and 0.5 mM dithiothreitol) supplemented with 0.5 mM ATPγS at 5 μl min^−1^ for 500 s. Then, SPR^RecA(E38K)^ supplemented with 0.5 or 1 mM ATPγS buffer was flowed in at 5 μl min^−1^ for 2500 s, in order to stabilize the formed filaments. From 3000 s, 1 μM RecA(E38K) in SPR^RecA(E38K)^ buffer supplemented with 0.5 mM ATPγS was injected at a flow rate of 5 μl min^−1^ for 4200 s.

Pol IV association with RecA(E38K)-dsDNA filaments was observed by injecting 0.65 μM pol IV in SPR^RecA(E38K)^ buffer supplemented with 0.5 mM ATPγS for 220 s at 5 μl min^−1^, monitoring pol IV association. From 220 s, buffer containing 0.5 mM ATPγS was flowed in at 5 μl min^−1^ and fast dissociation of pol IV was observed. Similarly, pol IV association with dsDNA was monitored, giving a lower response curve. We also observed non-specific binding of pol IV to the chip surface, making it impossible to measure binding kinetics of pol IV.

The surface was regenerated as previously reported (57). Furthermore, the SPR signal was corrected using a flow cell without immobilized bio-(dT)_71_ or dsDNA and corrected for the amount of immobilized RecA(E38K) (57). Ghodke *et al.* utilized this assay to monitor the binding kinetics of mCI, a probe derived from the bacteriophage λ repressor CI, at RecA-ssDNA filaments (57).

### DNA substrates for ATPase and LexA cleavage assay

M13mp18 cssDNA was purified as previously described (68), and M13mp18 cdsDNA was prepared as previously described (68–70). The M13mp18 nicked dsDNA was prepared by nicking with DNaseI according to manufacturer's recommendations. All DNA concentrations are given in terms of total nucleotides.

### ATPase assay

Adenosinetriphosphate (ATP) hydrolysis of wild-type RecA and RecA(E38K) on nicked cdsDNA was measured using a spectrophotometric enzyme assay (71,72). ATP regeneration from phosphoenolpyruvate and ADP was coupled to the oxidation of NADH, which was monitored by the decrease in absorbance of NADH at 380 nm. A total of 380-nm light was used so that the signal remained within the linear range of the spectrophotometer during the experiment. The assays were carried out on a Varian Cary 300 dual beam spectrophotometer equipped with a temperature controller and a 12-position cell changer. The cell path length and band pass were 0.5 and 2 nm, respectively. The NADH extinction coefficient at 380 nm of 1.21 mM^−1^ cm^−1^ was used to calculate the rate of ATP hydrolysis.

The reactions were carried out at 37°C in a buffer containing 25 mM Tris-Ac (80% cation, pH 7.5), 3 mM potassium glutamate, 10 mM magnesium acetate, 5% (w/v) glycerol, 1mM dithiothreitol, an ATP regeneration system (10 units/ml pyruvate kinase, 3 mM phosphoenolpyruvate) and a coupling system (2 mM NADH and 10 units/ml lactate dehydrogenase). The concentration of DNA (pEAW951 nicked cdsDNA) was 5 μM. One cuvette was a blank control that contained everything except the DNA (volume compensated with TE). The nicked cdsDNA, buffer, and ATP regeneration system were preincubated at 37°C for 10 min before addition of 3 mM ATP and 3 μM wild-type RecA or RecA(E38K). The process of data collection was then started.

### LexA cleavage assay

The cleavage of LexA was performed essentially as previously described (73). Reaction mixtures (125 μl) contained 40 mM Tris–HCl, pH 8.0, 10 mM MgCl_2_, 30 mM NaCl, 2 mM dithiothreitol, 3 μM of M13mp18 circular single-stranded DNA or pEAW951 nicked circular double-stranded DNA, 3 mM ATPγS, LexA and RecA as noted. Reactions were incubated at 37°C for 10 min before addition of LexA. The reaction products were separated and visualized by 15% SDS-PAGE stained with Coomassie blue.

### Western blotting

To analyze the cleavage of UmuD in cells treated with antibiotics, SOS-normal (RW120) and SOS-constitutive (RW546) strains were transformed with pRW154, which expresses the UmuD and UmuC proteins from their native promoter (49). Overnight *E. coli* LB cultures of RW120/pRW154 and RW546/pRW154 were diluted 1–100 in fresh LB with appropriate antibiotics and grown to mid-log (∼OD 0.5, ∼3 h). Aliquots were then taken for the untreated samples. Either ciprofloxacin (30 ng/ml) or trimethoprim (1 μg/ml) was added to the remaining culture and incubated with or without the addition of 2% DMSO. Samples were taken at 1, 2 and 3 h. Whole cell extracts were made by centrifuging 1.5 ml of culture and adding 90 μl of sterile deionized water and 30 μl of NuPAGE LDS sample buffer (4×) (Novex, Life Technologies) to the cell pellet. Five cycles of freeze/thaw on dry ice and in a 37°C water bath were performed to lyse the cells. Extracts were boiled for 5 min prior to loading. Samples were run on NuPAGE 4–12% Bis-Tris gels (Novex Life Technologies) and transferred to Invitrolon PVDF (0.45 μm pore size) membranes (Novex Life Technologies). Membranes were incubated with anti-UmuD antibodies (54) (1:5000 dilution) at room temperature overnight. Then the membranes were incubated with goat anti-rabbit IgG (H+L) alkaline phosphatase conjugate (1:10 000 dilution) (BIO-RAD). Subsequently, the membranes were treated with the CDP-Star substrate (Tropix). Membranes were then exposed to BioMax XAR film (Carestream) to visualize UmuD protein bands.

## RESULTS

### Induction of DNA double-strand breaks by ciprofloxacin and trimethoprim

We first investigated how many DSBs are created following ciprofloxacin and trimethoprim treatment. We imaged cells expressing a fluorescent fusion of the DSB reporter MuGam (56) to the photoactivatable mCherry protein (PAmCherry1 (57)). The data collection strategy is summarized in Supplementary Figure S1. MuGam-PAmCherry was expressed from pBAD-derived plasmid pEAW1162, which was introduced into the MG1655 wild-type *E. coli* background. For these single-molecule microscopy experiments, cells were grown in medium containing 0.003% L-arabinose. These conditions supported MuGam expression levels that had minimal effects on survival upon drug treatment (Supplementary Figure S2A) and yielded satisfactory MuGam-PAmCherry signals for imaging (Supplementary Figure S2B).

**Figure 1.**
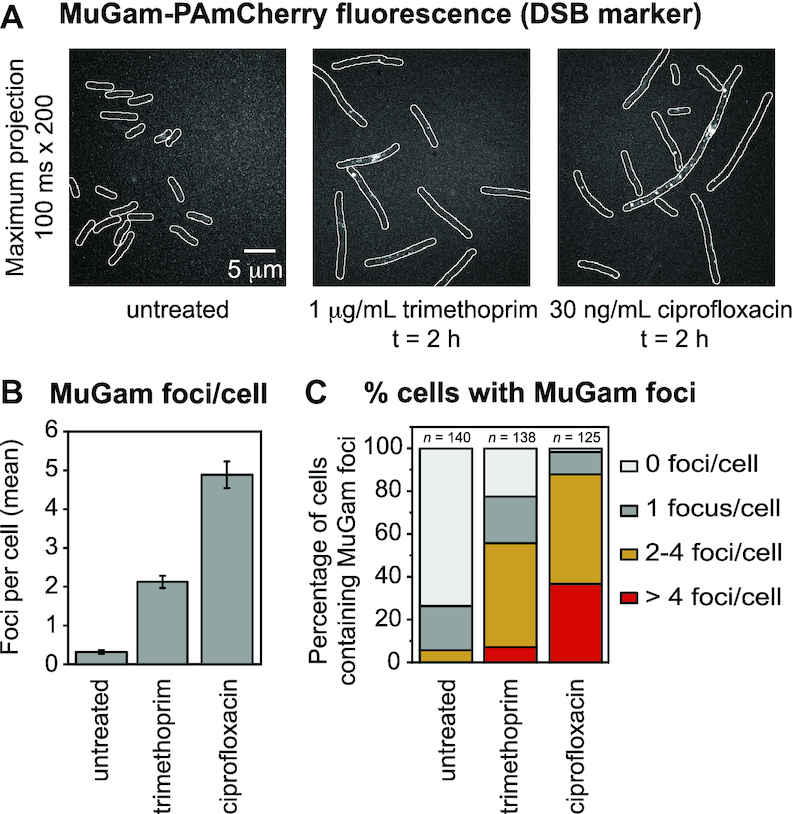
Number of MuGam-PAmCherry foci per cell following ciprofloxacin or trimethoprim treatment. *Escherichia coli* MG1655 cells carrying plasmid pEAW1162 were imaged on a single-molecule sensitive fluorescence microscope. (**A**) Fluorescence signal from MuGam-PAmCherry at 0.003% L-arabinose: maximum intensity projections over 200 × 100 ms frames showing MuGam-PAmCherry foci. From left to right: MuGam signal with no antibiotic, 2 h treatment with 1 μg/ml trimethoprim, 2 h treatment with 30 ng/ml ciprofloxacin. (**B**) Mean number of MuGam foci per cell. Cells were left untreated (*n* = 140), treated with 1 μg/ml trimethoprim (*n* = 138), or treated with 30 ng/ml ciprofloxacin (*n* = 125). The error bars represent standard error of the mean over the number of cells. * for *P* < 0.05; ** for *P* < 0.01 in two-sample *t*-test for differences of means. (**C**) Percentage of cells containing MuGam foci: 0 foci (light gray), 1 focus (gray), 2–4 foci (amber) and >4 foci (red). Cells were left untreated (*n* = 140), treated with 1 μg/ml trimethoprim (*n* = 138), or treated with 30 ng/ml ciprofloxacin (*n* = 125).

In the absence of antibiotic, cells exhibited 0.3 ± 0.1 MuGam foci per cell (Figure 1A and B) with most cells (74%) containing no foci (Figure 1C). Trimethoprim treatment for 2 h (1 μg/ml trimethoprim) led to a distinct increase in MuGam foci (1.9 ± 0.1 foci per cell Figure 1B) with 78% of cells now containing foci (Figure 1C). These results are consistent with earlier reports that trimethoprim treatment can induce the formation of DSBs (44). Following ciprofloxacin treatment (30 ng/ml ciprofloxacin), cells contained even more MuGam foci (4.9 ± 0.3 foci per cell, Figure 1B) with 98% of cells containing foci (Figure 1C). Thus, the analysis indicated that both the trimethoprim and ciprofloxacin treatments promoted the formation of DSBs, with ciprofloxacin treatment inducing approximately 2.5-fold more DSBs than trimethoprim. Ciprofloxacin-induced DSBs have also been reported by Pribis *et al.* (39), using a similar MuGam reporter.

DSB formation in trimethoprim-treated cells is dependent on the intracellular production of ROS (44). Consistent with this, we observed that co-treatment of cells with the ROS-mitigating compound DMSO (74) reduced the activities of ROS-induced promoters (Supplementary Figures S3–5) and reduced DSB formation (Supplementary Figure S6) in response to trimethoprim exposure.

It was recently demonstrated that the killing of bacterial cells by ciprofloxacin is also strongly dependent on intracellular ROS production (42). We found that co-treatment with DMSO suppressed a significant portion of ciprofloxacin-induced DSBs (Supplementary Figure S6). This seemingly conflicts with conclusions made during two recent studies (39,75). Pribis *et al.* concluded that mitigation of ROS did not prevent DSB formation in ciprofloxacin-treated cells (39). They reported that ∼80% of cells contained MuGam foci following ciprofloxacin treatment, both in the absence of ROS mitigators, and in the presence of either 2,2′-bipyridine or thiourea. In a second recent study, which included analysis of DSB formation in cells treated with another quinolone, nalidixic acid, Hong *et al.* concluded that mitigation of ROS did not prevent DSB formation (75). In that study, DSB formation in cells was monitored using a fluorescent fusion of the RecN protein, a recombination mediator that is involved in DSB repair. The authors observed that 1 h after treatment 97% of cells contained foci in the absence of ROS mitigators, compared with 81% of cells in the presence of bipyridyl and thiourea. In the current study we observed that very similar proportions of cells exhibited MuGam foci following ciprofloxacin treatment, with 98% of cells containing foci in the absence of ROS mitigators and 79% containing foci in the presence of DMSO (Supplementary Figure S6B). This result is in good agreement with the data presented in the Pribis *et al.* and Hong *et al.* studies. Notably however, we observed that the mean number of MuGam foci per cell was significantly reduced in the presence of the ROS-mitigating agent DMSO (2.2 ± 0.2 foci per cell; (Supplementary Figure S6C) compared with treatment in the absence of ROS mitigators (4.9 ± 0.3 foci per cell, Figure 1B). The mean number of DSBs per cell was not reported in either the Pribis *et al.* or Hong *et al.* studies. We conclude that fewer DSBs are formed in ciprofloxacin-treated cells when the damaging effects of ROS are mitigated with DMSO.

### DSB resection is the primary SOS response trigger in ciprofloxacin- and trimethoprim-treated cells

Damage-induced upregulation of pol IV expression is facilitated by the SOS response (reviewed in (76)). SOS is triggered by the presence of RecA* nucleoprotein filaments, which form on regions of ssDNA (77). RecA* filaments formed on ssDNA gaps and end-resected DSBs both act as SOS triggers (78). Both trimethoprim and ciprofloxacin are known to induce the SOS response (39,79). Following treatment with each drug, we investigated the degree to which induction of the SOS response depends on DSB repair via the RecBCD pathway, and thus the level of SOS-induced pol IV upregulation. We repeated the time-lapse experiments on cells that carried an SOS-reporter plasmid, in which GFP is expressed from the SOS-inducible *sulA* promoter (pUA139-P*_sulA_*-*gfp*; fast-folding GFP, *gfpmut2* (80)).

**Figure 2.**
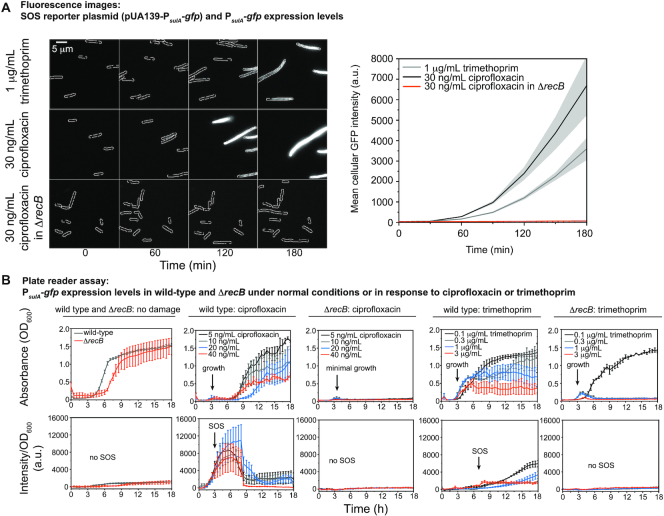
P*_sulA_-gfp* expression levels (SOS response levels) following ciprofloxacin or trimethoprim treatment is *recB*-dependent. (**A**) Left panel: Fluorescence images showing the expression of GFP from an SOS reporter plasmid (pUA139-P*_sulA_-gfp*) at 0, 60, 120 and 180 min (left to right) after treatment with 1 μg/ml trimethoprim (MG1655 cells), 30 ng/ml ciprofloxacin (MG1655 cells) or 30 ng/ml ciprofloxacin (Δ*recB* cells; HG356) (top to bottom). Scale bar represents 5 μm. Right panel: Quantitation of GFP expression levels. Mean cell intensity is plotted against time (trimethoprim: light gray line, ciprofloxacin: dark gray line, ciprofloxacin in Δ*recB*: red line). In this analysis the precise number of cells included is not determined but is well in excess of 100 cells at each time point. Gray-shaded error bands represent standard error of the mean. (**B**) P*_sulA_-gfp* expression levels in wild-type (MG1655) and Δ*recB* cells (HG356). For each strain, 10^4^–10^6^ cells were added to each well at the beginning of the experiment. Measurements of absorbance (OD_600_) and fluorescence intensity (a.u.) were carried out every 30 min over 18 h. Upper row shows absorbance (OD_600_) and bottom row illustrates intensity values/OD_600_, consistent with expression levels. Error bars represent standard error of the mean over three independent biological replicates. First column: Normal growth condition for wild-type or Δ*recB* cells (wild-type: dark gray; Δ*recB*: orange). Second column: ciprofloxacin treatment of wild-type cells (5 ng/ml: black; 10 ng/ml: gray; 20 ng/ml: blue; 40 ng/ml: orange). Third column: ciprofloxacin treatment of Δ*recB* cells (same color coding as second column). Fourth column: trimethoprim treatment of wild-type cells (0.1 μg/ml: black; 0.3 μg/ml: gray; 1 μg/ml: blue; 3 μg/ml: orange). Fifth column: trimethoprim treatment of Δ*recB* cells (same color coding as fourth column).

In the absence of any antibiotic treatment, cells exhibited very low fluorescence, consistent with the repression of the *sulA* promoter in the absence of exogenously applied DNA damage (Figure 2A, ‘0 min’). Cells exposed to trimethoprim exhibited clear SOS induction (100-fold increase from 0 to 180 min in the mean cell intensity derived from GFP signal). Cells exhibited even more robust SOS induction upon treatment with ciprofloxacin as evidenced by the increase in GFP fluorescence in the 180 min time window after addition of ciprofloxacin (170-fold induction from 0 to 180 min, Figure 2A). These results were confirmed using plate-reader assays (Figure 2B; Supplementary Figure S7A and B).

Next, we monitored SOS induction in cells lacking *recB* (HG356 [Δ*recB*] + pUA139-P*_sulA_*-*gfp*) to determine if SOS induction by ciprofloxacin is dependent on DSB processing. The deletion of *recB* strongly inhibited the SOS response following ciprofloxacin treatment (0.4-fold induction at 180 min in comparison to *recB^+^*, Figure 2A). While *recB* deletions are known to reduce survival in cells treated with ciprofloxacin (81), we observed that most cells lacking *recB* continued to grow, forming short filaments and occasionally dividing, during the 180 min time-lapse measurement (Supplementary Figure S8A and Movie S1), indicating that the lack of SOS induction observed for ciprofloxacin-treated *recB*-deficient cells did not stem from gross inhibition of all cellular functions. Plate-reader assays did not reveal a sustained increase in cell density for *recB* deletion cells following ciprofloxacin treatment (Figure 2B, middle column), suggesting that the initial growth observed by microscopy stagnates soon after the 180 min observation window. Deletion of *recB* attenuated GFP levels to the same extent as observed in cells carrying an SOS-defective allele (RW1568, *lexA*[Ind^−^]), indicating that ciprofloxacin-induced SOS is attenuated completely in the *recB* strain (Supplementary Figure S8B and C).

Plate-reader assays demonstrated that induction of the SOS response upon trimethoprim exposure is also highly dependent on *recB* (Figure 2B, last column). In contrast, deletion of the *recF*, *O* and *R* genes (EAW693, Δ*recF* Δ*recO* Δ*recR*), which are associated with SOS induction *via* the ssDNA gap repair pathway, had little impact on SOS induction in both ciprofloxacin- and trimethoprim-treated cells (Supplementary Figure S9). Beyond this, the inclusion of ROS mitigators, which reduce the number of MuGam foci (DSB markers, Supplementary Figure S6), also reduced SOS-induced GFP levels (Supplementary Figures S7 and 10). Measurements within an SOS-constitutive strain (EAW287, *recA*[E38K]) confirmed that this reduction in signal was not caused by DMSO-induced quenching of the GFP signal (Supplementary Figure S11).

Taken together our measurements indicate that SOS induction levels are strongly dependent on *recB*-dependent DSB processing in cells treated with ciprofloxacin or trimethoprim. The observation that SOS induction depends strongly on *recB* (Figure 2 or Supplementary Figure S7), but not *recF*, *O* and *R* (Supplementary Figure S9; (47)), suggests that DSBs are formed frequently under both ciprofloxacin and trimethoprim conditions and that ssDNA gaps do not accumulate to appreciable levels or are subsequently converted to DSBs. The reduction of the number of DSBs (as monitored by counting MuGam-PAmCherry foci) in the presence of the ROS mitigator is consistent with the hypothesis that ssDNA gaps are converted to DSBs in cells.

### DNA polymerase IV activity correlates with number of DNA double-strand breaks

We next monitored changes in intracellular pol IV concentrations and levels of focus formation in antibiotic-treated cells, using a strain that expressed fluorescently tagged pol IV (DinB-YPet). Following ciprofloxacin treatment, we further investigated if pol IV colocalizes with RecA* structures, which can form at DSB repair intermediates. Using two-color imaging, we measured colocalization of fluorescently tagged pol IV with the fluorescently tagged RecA* probe, mCI (57). In our previous study we demonstrated that the DinB-YPet allele is functionally active (30). In the current study, we confirmed that cells expressing fluorescently tagged pol IV exhibited similar sensitivity to both ciprofloxacin and trimethoprim as wild-type MG1655 cells (Supplementary Figure S12, first two panels).

**Figure 3.**
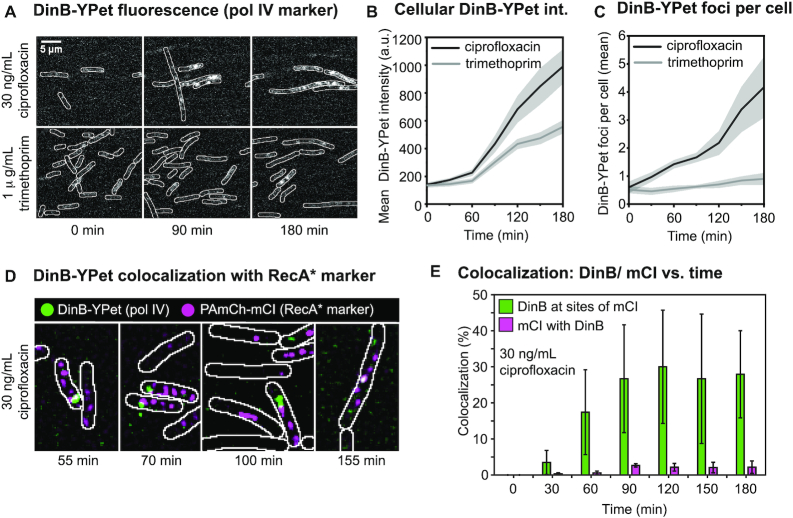
Pol IV concentration and focus formation following ciprofloxacin or trimethoprim treatment and pol IV colocalization with RecA* structures. (**A**) Fluorescence images showing cells (EAW643) expressing DinB-YPet (Pol IV) at 0, 90 and 180 min (left to right) after trimethoprim (1 μg/ml) or ciprofloxacin (30 ng/ml) treatment (top to bottom). Scale bar represents 5 μm. (**B**) Cellular DinB-YPet intensities during treatment. Mean cell brightness is plotted against time (1 μg/ml trimethoprim: light gray line; 30 ng/ml ciprofloxacin: dark gray line). In this analysis the precise number of cells included is not determined but is well in excess of 100 cells at each time point. Gray-shaded error bands represent standard error of the mean. (**C**) Number of DinB-YPet foci per cell are plotted against time (1 μg/ml trimethoprim: light gray line; 30 ng/ml ciprofloxacin: dark gray line). In this analysis, the precise number of cells included is not determined but is well in excess of 100 cells at each time point. Gray-shaded error bands represent standard error of the mean. (**D** and**E**) Colocalization between DinB and mCI after ciprofloxacin treatment (30 ng/ml) in EAW633 cells transformed with pEAW1162. (D) Merged images of discoidal filtered DinB-YPet (green) and PAmCherry-mCI (magenta) images at 55, 70, 100 and 155 min after ciprofloxacin addition (30 ng/ml). (E) Colocalization percentages after ciprofloxacin addition (30 ng/ml): percentage of DinB-YPet foci that colocalize with mCI features (green); percentage of mCI features that colocalize with DinB-YPet foci. Time points are grouped into 30 min bins. Error bar represents standard deviation of biological quadruplicates.

Trimethoprim treatment resulted in a clear increase in DinB-YPet intensity (Figure 3A) and was accompanied by cell filamentation (Supplementary Figure S13), a hallmark of the SOS response. 180 min after trimethoprim addition, the mean cellular fluorescence intensity (a proxy for intracellular DinB-YPet concentration) had increased by more than four-fold (intensity increase from 135 to 557, Figure 3B). Treatment with ciprofloxacin also led to filamentation (Supplementary Figure S13) and a significant increase in DinB-YPet fluorescence intensity (Figure 3A), resulting in an increase in the intracellular DinB-YPet concentration (7-fold increase from 140 to 990 mean cell brightness, Figure 3B). This increase is in line with the results of our previous study, in which we measured an increase in intracellular DinB-YPet (pol IV) concentrations from 6 ± 1 nM prior to treatment (standard error of the mean, SE) to 34 ± 3 nM (SE) 180 min after ciprofloxacin addition (30). Comparing intensity levels in our current and previous studies, we infer that the intracellular pol IV concentration is ∼23 nM in trimethoprim-treated cells and 34 nM in ciprofloxacin-treated cells. In general, conditions in which a large number of DSBs were detected yielded high intracellular concentrations of DinB-YPet (compare Figures 1B and 3B, Supplementary Figure S14A). This is consistent with the SOS-induction results described above (Figure 2) and suggests that DSB processing drives an increase in pol IV production via the SOS response.

Cells exhibit distinct pol IV foci when individual DinB-YPet molecules bind to DNA and thus experience decreased diffusional mobility (30,82). Since cells expressing a fluorescently tagged, catalytically dead mutant of pol IV do not exhibit foci (30), the foci observed in response to antibiotic treatment represent pol IV molecules that could be engaged in catalytic functions. Prior to the addition of antibiotic, cells contained on average 0.6 ± 0.2 foci per cell (SE) (Figure 3C). Trimethoprim treatment induced a slight increase in the number of DinB-YPet foci with 0.9 ± 0.2 per cell (SE) at 180 min. Following treatment with ciprofloxacin, the number of foci steadily increased, and by 180 min, cells exhibited 4.2 ± 1.1 foci per cell. Conditions in which a large number of DSBs were detected also yielded a large number of DinB-YPet (compare Figures 1B and 3B; Supplementary Figure S14B), suggesting that pol IV binding might be involved in DSB repair.

We next determined how frequently pol IV foci colocalized with RecA* structures formed at sites of DSB repair. The MuGam-PAmCherry probe used above to detect DSBs blocks DSB repair (56), thus we could not use MuGam-PAmCherry to test whether pol IV localized to DSB repair intermediates. Instead, we visualized the localizations of fluorescent pol IV (DinB-YPet) and a RecA* marker PAmCherry-mCI; a red fluorescent protein fusion of a monomeric C-terminal fragment of the λ repressor that retains the ability to bind RecA* in cells (57). We treated EAW633 (*dinB-YPet*) cells carrying pBAD-PAmCherry-mCI with ciprofloxacin, then carried out live-cell photoactivatable localization microscopy (PALM), collecting the signal of pol IV (DinB-YPet) and mCI foci (PAmCherry-mCI bound to RecA* structures). We have previously noted that most of the mCI foci appear at locations distal to the replisome in UV-irradiated cells (57).

Following ciprofloxacin treatment, cells typically contained multiple mCI foci (Figure 3D). At later time points, some cells contained more elongated ‘bundle’ structures as described previously (57). We next determined the percentage of DinB-YPet foci that colocalized with mCI foci and bundle-like structures (Figure 3E). Prior to the introduction of ciprofloxacin, mCI foci were rarely formed in cells during normal metabolism (<0.1 mCI foci per cell) consistent with our previous study (57). Unsurprisingly, we did not detect colocalization of pol IV with the RecA* probe in untreated cells (Figure 3E). Upon introduction of ciprofloxacin to the flow chamber, colocalization remained low during the early phase of the SOS response (i.e. between 0 and 45 min after treatment). From 45 min after the introduction of ciprofloxacin, pol IV exhibited significant colocalization (10–40%) with mCI in cells. This colocalization persisted into the late stages of SOS (up to 180 min after treatment). The measured colocalization represents an underestimate since the mCI probe sub-stoichiometrically labels RecA* filaments. Nevertheless, it is clear that RecA* structures represent a major site for pol IV activity in cells. A much lower proportion of mCI-labelled RecA* structures, 1–3%, spatially overlap with a pol IV focus (Figure 3E). This indicates that only a small subset of RecA* structures contain pol IV at any particular time.

### Double-strand break resection by RecBCD creates substrates for pol IV

We next set out to determine whether the formation of nucleoid associated pol IV foci requires DSB resection *via* the RecBCD pathway. We examined the extent of DinB-YPet focus formation in ciprofloxacin and trimethoprim treated cells, comparing backgrounds that permitted (*recB*^+^) or prevented (Δ*recB*) DSB resection. To separate effects on focus formation from effects on DinB-YPet expression, these measurements were performed in a *lexA*(Def) background (48) (*dinB-YPet dnaX-mKate2 lexA*[Def]). In these cells, constitutive cleavage of the mutant LexA repressor results in constitutive and elevated expression of the SOS induced genes including DinB-YPet even in the absence of exogenous DNA damage (30). To capture DinB-YPet binding events, we recorded burst acquisitions of the DinB-YPet signal (Supplementary Movie S2; 300 × 50 ms exposures taken every 100 ms; for further explanation of method see Supplementary Figure S1A and D).

**Figure 4.**
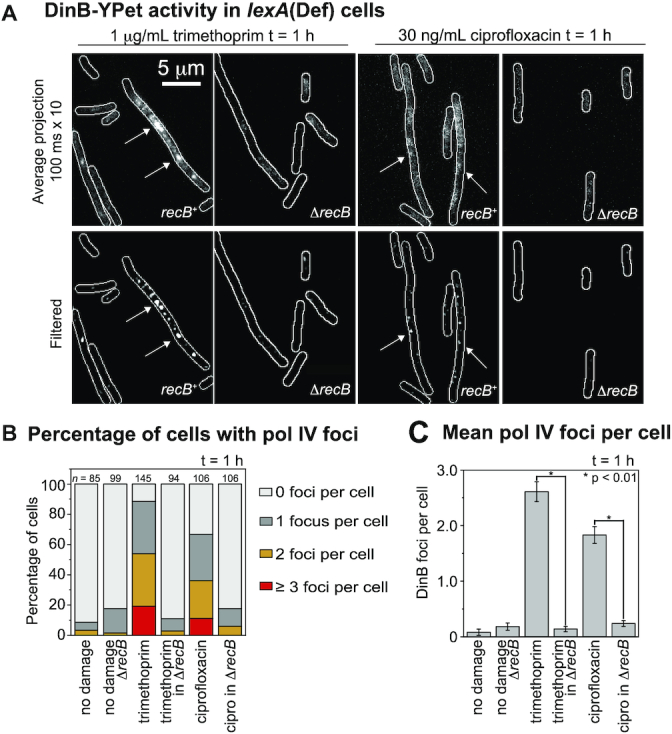
Number of pol IV foci per cell in *lexA*(Def) cells following ciprofloxacin (30 ng/ml) or trimethoprim (1 μg/ml) treatment is *recB*-dependent. (**A**) Upper row: Average projection in time (100 ms × 10 frames) showing DinB-YPet (pol IV) foci. Bottom row: Discoidal filtered projections. Columns 1 and 3: *recB*^+^ cells (EAW1141). Columns 2 and 4: Δ*recB* cells (EAW1144). Cells were treated for 60 min prior to imaging. (**B**) Percentage of cells containing pol IV foci: 0 foci (light gray), 1 focus (gray), 2 foci (amber) and ≥3 foci (red). Cells were treated with 30 ng/ml ciprofloxacin (*n* = 106), 30 ng/ml ciprofloxacin + 2% DMSO (*n* = 109), 30 ng/ml ciprofloxacin in Δ*recB* (*n* = 106), 1 μg/ml trimethoprim (*n* = 145), 1 μg/ml trimethoprim + 2% DMSO (*n* = 102), 1 μg/ml trimethoprim in Δ*recB* (*n* = 94) experienced no damage for wild-type (*n* = 85) and Δ*recB* (*n* = 99). (**C**) Number of DinB-YPet foci per cell. Error bars represent standard error of the mean. Number of cells included in analysis: *n*(untreated *recB*^+^) = 85, *n*(untreated Δ*recB*) = 99, *n*(trimethoprim) = 145, *n*(trimethoprim in Δ*recB*) = 94, *n*(ciprofloxacin) = 106, *n*(ciprofloxacin in Δ*recB*) = 106. * for *P* < 0.01 in two-sample *t*-test for differences of means.

Consistent with our previous results (30), few DinB-YPet foci were observed (Figure 4A) in *lexA*(Def) cells in the absence of either antibiotic (0.08 ± 0.05 foci per cell, Figure 4B and C), indicating that pol IV rarely binds to the nucleoid in the absence of exogenous DNA damage. In contrast, cells treated with trimethoprim for 60 min contained multiple DinB-YPet foci (2.6 ± 0.2 foci per cell, Figure 4B and C). Interestingly, trimethoprim-treated Δ*recB* cells contained very few foci (0.1 ± 0.05, Figure 4B and C), indicating that focus formation is strongly *recB*-dependent. Cells treated with ciprofloxacin for 60 min exhibited many foci (1.8 ± 0.15 foci per cell, Figure 4B and C), whereas cells lacking *recB* again produced very few foci (0.2 ± 0.05 foci per cell, Figure 4B and C). Taken together these results demonstrate that the formation of DinB-YPet foci is strongly dependent on the presence of the *recB* gene. This, in conjunction with the significant colocalization of pol IV foci and a RecA* marker (Figure 3D and E), implies that pol IV is predominantly active at DSB repair intermediates in cells treated with ciprofloxacin or trimethoprim. Additionally, ROS mitigation, which can reduce the number of DSB per cell (Supplementary Figure S6), also reduced the number of pol IV foci per cell (Supplementary Figure S15).

### Evidence supporting a physical interaction between pol IV and RecA* in cells

**Figure 5.**
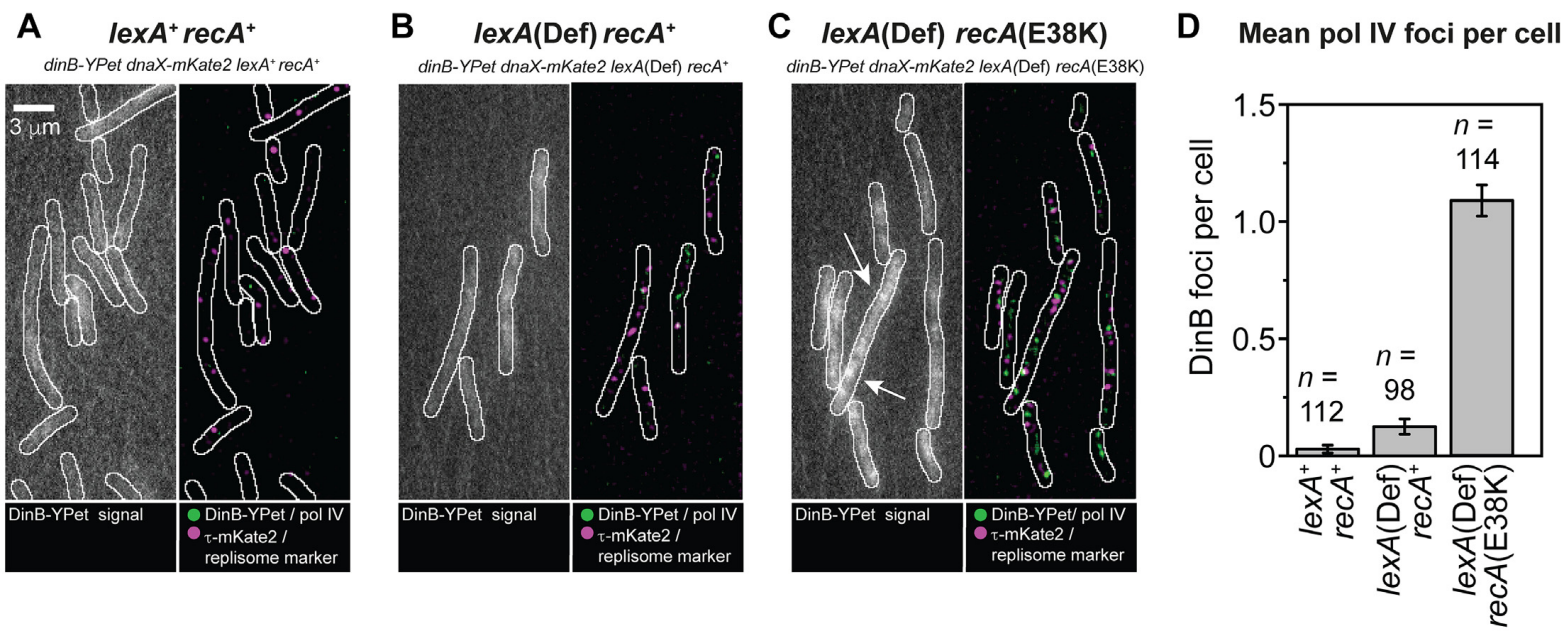
Formation of pol IV foci in *lexA* and *recA* mutants. (**A**) Images of DinB-YPet and DnaX-mKate2 signals in *lexA*^+^*recA*^+^ cells (EAW643). Left: Average projection of movie, producing an image with an effective exposure time of 300 ms. Right: Merged images of discoidal filtered DinB-YPet (green) and DnaX-mKate2 signals (magenta). (**B**) similar analysis for *lexA*(Def) *recA*^+^ cells (RW1594). (**C**) similar analysis for *lexA*(Def) *recA*(E38K) cells (RW1598). (**D**) Number of DinB-YPet foci per cell. Error bars represent standard error of the mean. Number of cells included in analysis: *n*(*lexA*^+^*recA*^+^) = 112, *n*(*lexA*[Def] *recA*^+^) = 98, *n*(*lexA*[Def] *recA*[E38K]) = 114.

Previous reports (8,38) provided *in vitro* evidence of a physical interaction between pol IV and RecA, which is likely to be relevant to its role in homologous recombination. We next determined whether a physical interaction between pol IV and RecA* occurs *in vivo*. Evidence supporting such an interaction arose from a series of measurements involving a mutant form of RecA. We observed that cells carrying the *recA*(E38K) mutation (also known as *recA730*) produce multiple pol IV foci, even in the absence of exogenous DNA damage (Figure 5; *lexA*[Def] *recA*[E38K]: 1.2 ± 0.2 foci per cell; *lexA*[Def] *recA*^+^: 0.2 ± 0.1; *lexA*^+^*recA*^+^: 0.1 ± 0.05). A strain expressing a catalytically dead version of the pol IV probe (*dinB*[D103N-YPet]) produced a similar number of foci to the strain expressing the catalytically competent probe (Supplementary Figure S16), indicating that foci formed in the *recA*(E38K) background are unlikely to represent pol IV molecules engaged in DNA synthesis.

*In vitro*, the RecA(E38K) mutant protein readily forms RecA*-like structures that are each competent for LexA cleavage on both ssDNA and dsDNA (Supplementary Figure S17 and 18). *In vivo*, RecA(E38K) constitutively promotes induction of the SOS response (83). Since RecA(E38K) readily binds dsDNA *in vitro* and cleaves LexA on dsDNA-RecA(E38K) filaments, we suggest that in the absence of exogenous DNA damage, most of the RecA(E38K)* structures in cells would form on dsDNA. In undamaged *recA*(E38K) cells, pol IV could form foci as it physically associates (unproductively) with these dsDNA-nucleated RecA(E38K)* structures. Surface plasmon resonance analysis confirmed such an interaction occurs *in vitro* (Supplementary Figure S17E and F). In wild-type (*recA*^+^) cells, foci would form as pol IV associates with RecA* structures that form as intermediates during DSB repair following ciprofloxacin or trimethoprim treatment. In sum, we infer from the data that pol IV physically interact with RecA(E38K)* structures *in vivo* and observe that pol IV and mCI colocalize at sites of RecA* filaments. From this, we infer that in wild-type cells pol IV physically interacts with RecA* filament structures engaged in homologous recombination.

### Focus formation by pol V does not correlate with DSBs

Finally, we used the single-molecule imaging approach to explore if the formation of DSBs affected the nucleoid-binding activity of the other major error-prone polymerase present in *E. coli*, pol V (UmuD′_2_C). Formation of the active form of pol V, known as pol V Mut (UmuD′_2_-UmuC-RecA-ATP), occurs through a series of tightly regulated steps (84,85). In cells, UmuC foci might be expected to form at two different stages of this activation process; when pol V Mut is formed through the interaction of pol V (UmuD′_2_-UmuC) with RecA* filaments (84,85), and when active pol V Mut complexes synthesize DNA (50).

Since pol V is also a member of the SOS regulon (31,84), we again used the *lexA*(Def) background (RW1286, *umuC-mKate2 dnaX-YPet lexA*[Def]) to separate effects on focus formation from effects on UmuC-mKate2 expression. As before, *lexA*(Def) cells were treated for 60 min with ciprofloxacin or trimethoprim (Supplementary Figure S1A). Movies of the UmuC-mKate2 signal were recorded (for further explanation see Supplementary Figure S1D, 300 × 50 ms exposures taken every 100 ms). We additionally monitored focus formation in cells that were co-treated with the ROS-mitigator DMSO, which reduces DSB formation in both ciprofloxacin- and trimethoprim-treated cells (Figure 1).

**Figure 6.**
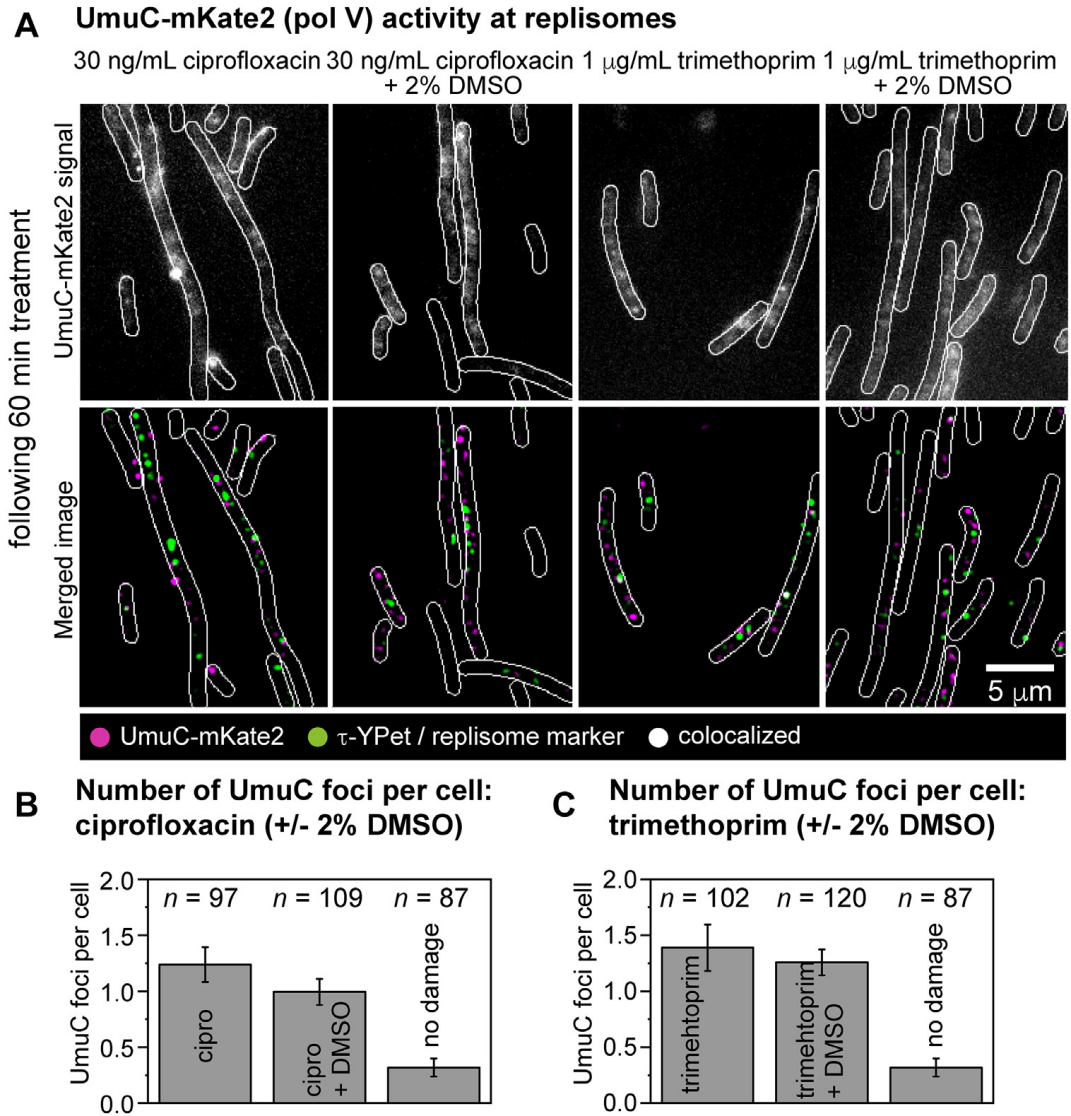
Measuring the number of pol V foci per cell following ciprofloxacin (30 ng/mL) or trimethoprim (1 μg/ml) treatment under normal conditions or ROS-mitigating (2% DMSO) conditions in *lexA*(Def) cells. (**A**) Formation of UmuC-mKate2 foci in *lexA*(Def) cells (RW1286). Cells were treated for 60 min prior to imaging. Upper row: unfiltered image of an average projection showing UmuC-mKate2 foci that persist for >1 s (from left to right: ciprofloxacin, ciprofloxacin-DMSO, trimethoprim, trimethoprim-DMSO). Bottom row: merged image showing UmuC-mKate2 foci in magenta and DnaX-YPet foci in green (from left to right: ciprofloxacin, ciprofloxacin-DMSO, trimethoprim, trimethoprim-DMSO). Scale bar represents 5 μm. (**B**) Number of UmuC-mKate2 foci per cell of foci that persist for >1 s following ciprofloxacin treatments (30 ng/ml). Error bars represent standard error of the mean. Number of cells included in analysis: *n*(ciprofloxacin) = 97, *n*(ciprofloxacin-DMSO) = 109, n(untreated) = 87. (**C**) Number of UmuC-mKate2 foci per cell of foci that persist for >1 s following trimethoprim treatments (1 μg/ml). Error bars represent standard error of the mean. Number of cells included in analysis: *n*(trimethoprim) = 102, n(trimethoprim-DMSO) = 120, n(untreated) = 87.

Few UmuC-mKate2 foci were observed (Figure 6A) in the absence of antibiotic in *lexA*(Def) cells (0.3 ± 0.1 foci per cell, Figure 6B). In *lexA*(Def) cells treated with ciprofloxacin or trimethoprim for 60 min, foci were clearly visible (ciprofloxacin: 1.2 ± 0.2 foci per cell,; trimethoprim 1.4 ± 0.2 foci per cell, Figure 6B). In both cases, co-treatment with DMSO to reduce the number of DSBs had little effect on the number of UmuC-mKate2 foci (ciprofloxacin-DMSO: 1.0 ± 0.1 foci per cell; trimethoprim-DMSO 1.3 ± 0.2 foci per cell) or on the overall levels of UmuC-mKate2 fluorescence in the cells. Thus, in contrast to the effects observed for pol IV (Supplementary Figure S15), the addition of DMSO had little effect on pol V focus formation.

In trimethoprim-treated *lexA*^+^ cells (SOS is damage-induced) independently of DMSO co-treatment, UmuC-mKate2 was primarily associated with the cell membrane (Supplementary Figure S19). In a previous study, we showed that this phenomenon stems from a form of spatial regulation in which UmuC is only released from the cell membrane once sufficient cleavage of UmuD_2_ to UmuD′_2_ has taken place (50). Consistent with this, western blots showed that the levels of both UmuD_2_ and UmuD′_2_ were much lower in trimethoprim-treated cells than in ciprofloxacin-treated cells (Supplementary Figure S20B and D).

## DISCUSSION

Previous work established that pol IV foci increase in response to DNA damage and SOS induction, although most are not associated with replisomes. The current study extends this work, providing four main conclusions which intimately link pol IV activity to DSB repair: (i) SOS-associated upregulation of pol IV production in response to DSBs induced by trimethoprim or ciprofloxacin treatment is blocked in a *recB* mutant (Figures 1–3). (iii) The formation of pol IV foci, which requires the catalytic activity of pol IV and is indicative of pol IV binding to substrates on the nucleoid, is drastically reduced in a *recB* mutant (Figure 4). (iii) Binding of pol IV to the nucleoid involves a physical interaction between pol IV and RecA* nucleoprotein filaments (Figure 5). (iv) The number of foci formed by the other Y-family polymerase present in *E. coli*, pol V, does not correlate with the number of DSBs formed (Figure 6). From these results we infer that DSB repair intermediates are the major substrates for pol IV activity in ciprofloxacin and trimethoprim treated cells. In contrast, pol V either does not act in the DSB repair pathway or does so rarely. Thus, pol IV has a major role in DSB repair that is not shared by pol V.

### Pol IV works on recombination intermediates

**Figure 7.**
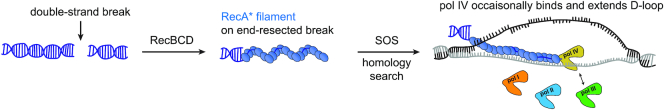
Model for pol IV extending D loops. Double-strand breaks are processed by the RecBCD helicase-nuclease multiprotein complex. RecBCD resects breaks and loads RecA on the single-stranded DNA regions, forming RecA* filaments. RecA* filaments are competent to induce the SOS response while undergoing homology search. Once a homologous sequence is found in the genome, a D loop can form which can be extended by certain DNA polymerases. Pol IV, for instance, can participate in recombination reactions following RecBCD activity.

We showed that the *recB* gene, which encodes a key component of the RecBCD helicase-nuclease complex that processes DSBs, is required for formation of the vast majority of pol IV foci formed in cells treated with ciprofloxacin or trimethoprim (Figure 4). Our observations are consistent with previously proposed models in which pol IV carries out repair synthesis during DSB repair (16–24,39,86). The results described here provide the first indication that once DSB repair intermediates begin to appear in cells treated with DSB-inducing antibiotics, they become the predominant substrates for pol IV-dependent DNA synthesis. Prior to treatment, a small number of pol IV foci are present (1 focus for every two cells on average, (30)). The majority of these (90%) do not colocalize with replisomes (30). Beginning 30 min after treatment, the number of mCI features (which form at RecA* structures) steadily increase as the cells begin to repair DSBs (Figure 3). In parallel there is a concordant increase in the number of pol IV foci. As described in our previous work a replisome-proximal subpopulation of pol IV foci remains up until 90 min post-treatment (30). This subpopulation disappears abruptly after 90 min. During the late SOS response (90–180 min after the onset of treatment) cells contain 2–4 pol IV foci on average. The majority of these foci (90–95%) are *recB*-dependent (Figure 4) and many of them colocalize with RecA* structures (Figure 3). The most likely role for pol IV in this context would be extension of D-loop intermediates (Figure 7).

Focus formation by fluorescently tagged pol IV requires the catalytic activity of pol IV in the wild-type *recA*^+^ background (30), suggesting that pol IV might specifically associate with available 3′ termini on RecA*-coated substrates, leading to pol IV-dependent DNA synthesis, as opposed to binding unproductively along the entire length of the RecA* nucleoprotein filament. It is important to note that pol IV is not the sole DNA polymerase charged with carrying out repair synthesis at DSB repair intermediates; pol III is almost certainly involved (87) and pols I and II likely also play roles (88–90). Our results instead indicate that when pol IV acts within cells treated with ciprofloxacin or trimethoprim, it acts within the DSB repair pathway. We hypothesize that D-loop extension by pol IV may be particularly relevant under conditions of heavy DNA damage, in which the template strand may itself contain lesions and thus require TLS activity to complete recombination. The observation that a relatively small proportion of RecA* structures contain a spatially overlapping pol IV focus (Figure 3E) indicates that at any particular time, only a subset of DNA repair-intermediates contain pol IV. There are two potential explanations for this, which are not mutually exclusive. First, pol IV might bind to a subset of RecA* structures. This would indeed be expected as RecA* structures are present within multiple intermediates that form during DSB repair—at end-resected breaks, in molecules undergoing homology search, and in D-loops—yet only D-loops would be suitable substrates for pol IV-mediated DNA synthesis. Second, binding of pol IV at D-loops may be transient. Here, pol IV would occasionally take over the substrate from pol III, synthesize a short patch of DNA, then dissociate, handing the substrate back to pol III (Figure 7). In our previous study, we monitored the formation and loss of pol IV foci as a function of time, in the milliseconds–seconds range (30). We observed signal fluctuations that would be consistent with a scenario in which pol IV remains bound at substrates for a few hundred milliseconds. This observation, while far from conclusive, is supportive of transient binding. In principle the exchange of pol III for pol IV at D-loops could occur stochastically, or alternatively, might be directed by the presence of lesions in the DNA being used as the template for repair synthesis.

The results of the current study support another recent study, which points to error-prone break repair as a major mechanism for ciprofloxacin-induced mutagenesis (39). In the Pribis *et al.* study, increased mutagenesis was found to be concentrated within a small sub-population of cells, and pol IV was found to be a major driver of the increased mutagenesis (along with pols II and V). In principle, this mutagenesis could arise through one of two scenarios. In the first scenario, pol IV only acts at break-repair intermediates in a subset of cells, and always acts in an error-prone fashion. In the second scenario, pol IV acts at repair intermediates in all cells, but is error-prone in a sub-set of cells. In both the current study and our microscopy study of pol IV (30), we observed increases in pol IV expression and focus formation following ciprofloxacin treatment that were relatively uniform across cells. Our observations appear to be most consistent with the model in which pol IV acts at double-strand breaks in all cells, but becomes error-prone in a sub-set of cells that express high levels of RpoS. The mechanism for this change in the fidelity of pol IV-dependent repair synthesis is not yet clear.

Mutagenic effects that arise from pol IV acting in DSB repair are now well documented (18–20,36,39). It is pertinent to consider whether another class of pol IV-dependent phenomena, in which pol IV promotes the survival of cells exposed to certain alkylating agents (8,17,23,91), might also stem from pol IV acting at DSB repair intermediates, rather than at stalled replication forks as is often assumed. Indeed, the authors of a study into the genetic determinants of 4-nitroquinoline-1-oxide resistance concluded that pol IV, as well as pol II, were likely acting through recombination pathways (17).

### Pol V is not activated by ROS-induced damage

In contrast to the observations made for pol IV, the formation of pol V foci did not appear to correlate with levels of DSB formation in *lexA*(Def) cells (Figure 6). This implies that DSB repair intermediates are unlikely to serve as major substrates for pol V-dependent synthesis. In the wild-type *lexA*^+^ background, interesting differences in pol V behavior were observed between cells treated with ciprofloxacin or trimethoprim. Intracellular pol V levels barely increased following trimethoprim treatment (Supplementary Figure S18). Thus unlike pol IV, the formation (and subsequent repair) of DSBs is not sufficient to trigger increased levels of pol V. Far less UmuD_2_ and UmuD′_2_ are produced in trimethoprim-treated cells than in ciprofloxacin-treated cells (Supplementary Figure S20). This presumably limits the amount of pol V (UmuD′_2_-UmuC) produced in trimethoprim-treated cells.

## Supplementary Material

gkaa597_Supplemental_Files
