# Supplementary material for: Single-molecule live-cell imaging reveals RecB-dependent function of DNA polymerase IV in double strand break repair
Source: Nucleic Acids Res. 2020 Jul 20;48(15):8490–508. doi: 10.1093/nar/gkaa597 (PMC7470938; doi:10.1093/nar/gkaa597)

Supplementary Figure S1. Experimental design. (A) Experimental setup. Cells are loaded in a flow-cell and immobilized on a positively charged aminopropyl silane glass surface. Cells were imaged before and after antibiotic exposure ± ROS mitigator. Time-lapse movies were recorded to follow the cellular response. Burst acquisitions were recorded to follow the dynamic behavior of fluorescent protein fusion constructs in cells. (B) Time-lapse movies were recorded over 3 h following the cellular response to antibiotic exposure. An image was taken every 10 min. At t = 0 min, the first image was taken and subsequently antibiotic-containing media was flowed into the flow cell. A total number of 19 frames was recorded. (C) Burst acquisition videos were recorded at specific time-points before or after antibiotic addition. Movies of MuGam-PAmCherry were recorded, containing 200 frames at 100 ms exposure. (D) Burst acquisition movies of DinB-YPet or UmuC-mKate2 were recorded, containing 300 frames at 50 ms exposure followed by 50 ms dark time.

Supplementary Figure S2. Determining L-arabinose concentration for PamCherry-MuGam expression for imaging. (A) Plate-based survival assays using ciprofloxacin or trimethoprim at different PAmCherry-MuGam expression levels. Wild-type **MG1655** cells carrying a pBAD plasmid for PAmCherry-MuGam expression (pEAW1162) were grown in EZ glycerol in the presence of ampicillin at different L-arabinose concentrations (0, 0.001, 0.003, 0.01, 0.03, 0.1% wt/vol) or in EZ glucose in order to inhibit expression from the pBAD plasmid. These cultures were split in three to perform two survival assays and a 'no damage' control. For the survival assays, antibiotic was added to these cultures (30 ng/mL ciprofloxacin or 1 μg/mL trimethoprim), then, cell cultures were grown for 2 h. For the control, cells were grown in the absence of antibiotic for 2 h. After 2 h of growth, cultures were centrifuged and resuspended in glucose or glycerol containing media (x 3) to remove the antibiotic. These cultures were serial diluted in PBS by factor ten down to 10^{-5} and spotted onto LB agar plates containing 100 μg/mL ampicillin. At an L-arabinose concentration of 0.003% (orange box), no drastic decrease in survival was observed in comparison to the sample grown in EZ glucose. (B) Fluorescence signal from MuGam-PAmCherry at 0.003% L-arabinose of *E. coli* MG1655

cells carrying plasmid pEAW1162: maximum intensity projections over 200 x 100 ms frames showing MuGam-PAmCherry foci. From left to right: MuGam signal with no antibiotic, 2 h treatment with 1 μg/mL trimethoprim, 2 h treatment with 30 ng/mL ciprofloxacin.

Supplementary Figure S3. P_{sodA}-*gfp* expression levels wild-type cells. 10^4 – 10^6 cells (MG1655 carrying pSTB-*sodA*-*gfp*) were added to each well at the beginning of the experiment. Measurements of absorbance (OD_{600}) and fluorescence intensity (a.u.) were carried out every 30 min over 17 h. For (A)-(C): upper row shows absorbance (OD_{600}) and bottom row illustrates intensity values/ OD_{600}, consistent with expression levels. Error bars represent standard error of the mean over three independent biological replicates. (A) *sodA* is regulated by SoxRS. Superoxides oxidize the Fe-S clusters of the SoxR transcription factor, promoting transcription of *soxS*. SoxS then acts as a transcription factor for *sodA*. For cells carrying P_{sodA}-*gfp*, superoxides then trigger the expression of GFP from the *sodA* promotor. (B) Comparison of normal growth condition with ciprofloxacin treatment ± ROS mitigator. First column: normal growth conditions (dark grey) or + 2% DMSO (grey); second column: ciprofloxacin treatment (5 ng/mL: black; 10 ng/mL: grey; 20 ng/mL: blue; 40 ng/mL: orange); third column: ciprofloxacin + 2% DMSO treatment (same color coding as second column). (C) Comparison of normal growth condition with trimethoprim treatment ± ROS mitigator. First column: as (A) first column; second column: trimethoprim treatment (0.1 µg/mL: black; 0.3 µg/mL: grey; 1 µg/mL: blue; 3 µg/mL: orange); third column: trimethoprim + 2% DMSO treatment (same color coding as second column). (D) Comparison of normal growth condition with hydrogen peroxide (H_2O_2) treatment ± ROS mitigator. First column: as (A) first column; second column: H_2O_2 treatment (30 mM: black; 100 mM: grey; 300 mM: blue; 500 mM: orange); third column: H_2O_2 + 2% DMSO treatment (same color coding as second column).

Supplementary Figure S4. P_{ahpC}-*gfp* expression levels wild-type cells. 10^4 – 10^6 cells (MG1655 carrying pCJH0008) were added to each well at the beginning of the experiment. Measurements of absorbance (OD_{600}) and fluorescence intensity (a.u.) were carried out every 30 min over 17 h. For (A)-(C): upper row shows absorbance (OD_{600}) and bottom row illustrates

intensity values/ OD_{600}, consistent with expression levels. Error bars represent standard error of the mean over three independent biological replicates. (A) *ahcP* is transcriptionally regulated by OxyR. Oxidation of OxyR cysteines induces transcription and expression of AhcP. For cells carrying P_{ahcP}-*gfp*, oxidative stress triggers the expression of GFP from the *ahcP* promotor. (B) Comparison of normal growth condition with ciprofloxacin treatment ± ROS mitigator. First column: normal growth conditions (dark grey) or + 2% DMSO (grey); second column: ciprofloxacin treatment (5 ng/mL: black; 10 ng/mL: grey; 20 ng/mL: blue; 40 ng/mL: orange); third column: ciprofloxacin + 2% DMSO treatment (same color coding as second column). (C) Comparison of normal growth condition with trimethoprim treatment ± ROS mitigator. First column: as (A) first column; second column: trimethoprim treatment (0.1 µg/mL: black; 0.3 µg/mL: grey; 1 µg/mL: blue; 3 µg/mL: orange); third column: trimethoprim + 2% DMSO treatment (same color coding as second column). (D) Comparison of normal growth condition with hydrogen peroxide (H_2O_2) treatment ± ROS mitigator. First column: as (A) first column; second column: H_2O_2 treatment (30 mM: black; 100 mM: grey; 300 mM: blue; 500 mM: orange); third column: H_2O_2 + 2% DMSO treatment (same color coding as second column).

Supplementary Figure S5. P_{fepD}-*gfp* expression levels wild-type cells., 10^4 – 10^6 cells (MG1655 carrying pCJH0009) were added to each well at the beginning of the experiment. Measurements of absorbance (OD_{600}) and fluorescence intensity (a.u.) were carried out every 30 min over 17 h. For (A)-(C): upper row shows absorbance (OD_{600}) and bottom row illustrates intensity values/ OD_{600}, consistent with expression levels. Error bars represent standard error of the mean over three independent biological replicates. (A) *fepD* is regulated by Fur. Under high iron conditions, transcriptional repressor Fur inhibits of *fepD* transcription. Under low iron conditions, in the presence of oxidative damage, Fur is de-repressed and *fepD* is transcribed. For cells carrying P_{fepD}-*gfp*, oxidative stress triggers the expression of GFP from the *fepD* promotor. (B) Comparison of normal growth condition with ciprofloxacin treatment ± ROS mitigator. First column: normal growth conditions (dark grey) or + 2% DMSO (grey); second

column: ciprofloxacin treatment (5 ng/mL: black; 10 ng/mL: grey; 20 ng/mL: blue; 40 ng/mL: orange); third column: ciprofloxacin + 2% DMSO treatment (same color coding as second column). (C) Comparison of normal growth condition with trimethoprim treatment ± ROS mitigator. First column: as (A) first column; second column: trimethoprim treatment (0.1 μg/mL: black; 0.3 μg/mL: grey; 1 μg/mL: blue; 3 μg/mL: orange); third column: trimethoprim + 2% DMSO treatment (same color coding as second column). (D) Comparison of normal growth condition with hydrogen peroxide (H_2O_2) treatment ± ROS mitigator. First column: as (A) first column; second column: H_2O_2 treatment of wild-type cells (30 mM: black; 100 mM: grey; 300 mM: blue; 500 mM: orange); third column: H_2O_2 + 2% DMSO treatment (same color coding as second column).

Supplementary Figure S6. Number of PAmCherry-MuGam foci per cell following ciprofloxacin or trimethoprim treatment under normal conditions or ROS-mitigating conditions in different genetic backgrounds. (A) Fluorescence signal from PAmCherry-MuGam at 0.003% L-arabinose: Maximum projections over 100 ms x 200 frames showing PAmCherry-MuGam foci. From left to right: MuGam signal after 2 h treatment with 30 ng/mL ciprofloxacin, 30 ng/mL ciprofloxacin + 2% DMSO, 1 μg/mL trimethoprim, 1 μg/mL trimethoprim + 2% DMSO, no damage. (B) Percentage of cells containing MuGam foci: 0 foci (light grey), 1 focus (grey), 2-4 foci (amber) and > 4 foci (red). Cells were treated with ciprofloxacin (*n* = 125), ciprofloxacin + DMSO (*n* = 127), trimethoprim (*n* = 138), trimethoprim + DMSO (*n* = 146), or experienced no damage (*n* = 140). (C) Mean number of MuGam foci per cell. Cells were treated with ciprofloxacin (*n* = 125), ciprofloxacin + DMSO (*n* = 127), trimethoprim (*n* = 138), trimethoprim + DMSO (*n* = 146), or experienced no damage (*n* = 140). The error bars represent standard error of the mean over the number of cells. * for $p < 0.05$; ** for $p < 0.01$ in two-sample t test for differences of means.

Supplementary Figure S7. P_{sulA}-*gfp* expression levels in wild-type and Δ*recB* cells. For each strain, 10^4 – 10^6 cells (MG1655 or EAW102 carrying pUA139-P_{sulA}-*gfp*) were added to each well at the beginning of the experiment. Measurements of absorbance (OD_{600}) and

fluorescence intensity (a.u.) were carried out every 30 min over 18 h. For (A)-(C): upper row shows absorbance (OD_{600}) and bottom row illustrates intensity values/ OD_{600}, consistent with expression levels. Error bars represent standard error of the mean over three independent biological replicates. (A) Comparison of normal growth condition with ciprofloxacin treatment ± ROS mitigator for wild-type cells or Δ*recB.* First column: normal growth conditions (wild-type: dark grey; Δ*recB*: orange), + 2% DMSO (wild-type: grey) or 0.35 mM BiP (wild-type: green); second column: ciprofloxacin treatment of wild-type cells (5 ng/mL: black; 10 ng/mL: grey; 20 ng/mL: blue; 40 ng/mL: orange); third column: ciprofloxacin + 2% DMSO treatment of wild-type cells (same color coding as second column); forth column: ciprofloxacin + 0.35 mM BiP treatment of wild-type cells (same color coding as second column); fifth column: ciprofloxacin treatment of Δ*recB* cells (same color coding as second column). (B) Comparison of normal growth condition with trimethoprim treatment ± ROS mitigator for wild-type cells or Δ*recB.* First column: as (A) first column; second column: trimethoprim treatment of wild-type cells (0.1 µg/mL: black; 0.3 µg/mL: grey; 1 µg/mL: blue; 3 µg/mL: orange); third column: trimethoprim + 2% DMSO treatment of wild-type cells (same color coding as second column); forth column: trimethoprim + 0.35 mM BiP treatment of wild-type cells (same color coding as second column); fifth column: trimethoprim treatment of Δ*recB* cells (same color coding as second column). (C) Comparison of normal growth condition with hydrogen peroxide (H_2O_2) treatment ± ROS mitigator for wild-type cells. First column: as (A) first column; second column: H_2O_2 treatment of wild-type cells (30 mM: black; 100 mM: grey; 300 mM: blue; 500 mM: orange); third column: H_2O_2 + 2% DMSO treatment of wild-type cells (same color coding as second column).

Supplementary Figure S8. P_{sulA}-*gfp* expression levels following ciprofloxacin-alone treatment in ΔrecB vs. MG1655 (wild-type). (A) Fluorescence images showing the expression of GFP from a SOS reporter plasmid (P*sulA*-GFP) from 0-110 min at intervals of 10 min and 120 min after 30 ng/mL ciprofloxacin addition in Δ*recB* (EAW102). Scale bar represents 5 µm. (B–C) Fluorescence microscopy analysis comparing the expression of GFP

from a SOS reporter plasmid (pUA139-P_{sulA}-*gfp*) in wild-type MG1655, Δ*recB* cells (EAW102), and *lexA*(Ind^{-}) cells (RW1568), 120 min after the addition of 30 ng/mL ciprofloxacin. (B) Representative fluorescence images. Scale bar represents 5 µm. (C) Mean cellular fluorescence intensities. Error bars represent the standard error of the mean.

Supplementary Figure S9. P_{sulA}-*gfp* expression levels in wild-type and Δ*recFOR* cells. For each strain, 10^5 – 10^6 cells were added to each well at the beginning of the experiment. Measurements of absorbance (OD_{600}) and fluorescence intensity (a.u.) were carried out every 30 min over 18 h. For (A)-(B): upper row shows absorbance (OD_{600}) and bottom row illustrates intensity values/ OD_{600}, consistent with expression levels. Error bars represent standard error of the mean over three independent biological replicates. (A) Comparison of normal growth condition with ciprofloxacin treatment for wild-type (MG1655) or Δ*recFOR* cells (EAW693). First column: normal growth conditions (wild-type: dark grey; Δ*recFOR*: grey); second column: ciprofloxacin treatment of wild-type cells (1 ng/mL: light grey; 10 ng/mL: grey; 20 ng/mL: blue; 40 ng/mL: orange); third column: ciprofloxacin treatment of Δ*recFOR* cells (same color coding as second column). (B) Comparison of normal growth condition with trimethoprim treatment for wild-type cells or Δ*recFOR.* First column: as (A) first column; second column: trimethoprim treatment of wild-type cells (0.1 µg/mL: light grey; 0.3 µg/mL: grey; 1 µg/mL: blue; 3 µg/mL: orange); third column: trimethoprim treatment of Δ*recFOR* cells (same color coding as second column).

Supplementary Figure S10. P_{sulA}-*gfp* expression levels (SOS response levels) following ciprofloxacin or trimethoprim treatment under normal or ROS-mitigating conditions in different genetic backgrounds. (A) Fluorescence images showing the expression of GFP from a SOS reporter plasmid (pUA139-P_{sulA}-*gfp*) at 0, 60, 120 and 180 min (left to right) after 30 ng/mL ciprofloxacin-alone, 30 ng/mL ciprofloxacin + 2% DMSO, 30 ng/mL ciprofloxacin-alone in Δ*recB* (EAW102), 1 µg/mL trimethoprim-alone, 1 µg/mL trimethoprim + 2% DMSO or 1 µg/mL trimethoprim – 0.35 mM BiP treatment (top to bottom). Scale bar represents 5 µm. (B) GFP expression levels from the *sulA* promotor during stress. Mean cell intensity is plotted

against time (ciprofloxacin-alone: dark grey line, ciprofloxacin-DMSO: light grey line, ciprofloxacin in Δ*recB*: purple, dotted line, trimethoprim-alone: red line, trimethoprim-DMSO: light red line, trimethoprim-BiP: rose-colored, dashed line). At each time-point, data are derived from >100 cells. Grey shaded error bands represent standard error of the mean.

Supplementary Figure S11. DMSO has no effect on GFP fluorescence *in vivo*. (A) Fluorescence images of *recA*[E38K] cells (RW244, constitutive SOS) carrying the SOS reporter plasmid (P_{sulA}-*gfp*) in the absence of DMSO (left) and in the presence of 2% DMSO (right). Scale bar represents 5 µm. (B) SulA expression levels. Mean cell brightness is plotted for *recA*[E38K] cells grown in the absence and presence of DMSO, showing that DMSO does not quench GFP signal. Error bars represent standard error of the mean from n > 100 cells.

Supplementary Figure S12. Survival of strains to ciprofloxacin and trimethoprim in EZ medium. Survival assays using ciprofloxacin or trimethoprim normal or ROS-mitigating condition (+ DMSO). Cell cultures (MG1655 [wild-type], *dinB-YPet dnaX-mKate2* [EAW643], Δ*dinB* [EAW18], *umuC-mKate2 dnaX-YPet* [EAW282] and Δ*umuDC* [RW880]) were grown in EZ glucose medium to exponential growth phase (OD_{600} = 0.2-0.3). Then, cultures were split in 6 before one sample was used as control, 2% DMSO, 30 ng/mL ciprofloxacin, 30 ng/mL ciprofloxacin + DMSO, 1 µg/mL trimethoprim or or 1 µg/mL trimethoprim + 2% DMSO were added in the others and grown for 60 min. Before the treatment and after 60 min samples were taken and serial diluted by factor ten down to 10^{-6}. Dilutions 10^{-1} to 10^{-6} of each culture were spotted on fresh LB plates, incubated in the dark overnight at 37ºC before the images were captured. Images selected are representative of a biological triplicate. Cells constructs used in this study (*dinB-YPet dnaX-mKate2* and *umuC-mKate2 dnaX-YPet*) exhibit a similar phenotype to MG1655.

Supplementary Figure S13. Scatter plots of cell-size from time-lapse imaging. White points indicate individual data-points, while blue-to-red contours indicate frequencies of observations. Blue areas indicate regions of the plot containing few data points; red areas

indicate regions containing a large number of data points. Frequencies were normalized at each time-point to the maximum value at that time-point with dark blue = 0 and dark red = 1. We conservatively estimate that >100 cells were used in each measurement. (A–D) EAW643 cells (*dinB-YPet dnaX-mKate2*) treated with (A) 30 ng/mL ciprofloxacin alone, (B) 30 ng/mL ciprofloxacin + 2% DMSO, (C) 1 μg/mL trimethoprim alone, (D) 1 μg/mL trimethoprim + 2% DMSO. (E–I) Wild-type cells (MG1655 carrying pUA139-P_{sulA}-*gfp*) treated with 30 ng/mL ciprofloxacin-alone, (F) 30 ng/mL ciprofloxacin + 2% DMSO, (G) 1 μg/mL trimethoprim-alone, (H) 1 μg/mL trimethoprim + 2% DMSO, (I) Wild-type cells (MG1655 carrying pUA139-P_{sulA}-*gfp*) treated with trimethoprim-BiP. (J) Δ*recB* cells (EAW102 carrying pUA139-P_{sulA}-*gfp*) treated with 30 ng/mL ciprofloxacin.

Supplementary Figure S14. Plots of cellular DinB-YPet concentrations and number of DinB-YPet foci per cell against the number of MuGam foci (DSBs) detected under similar conditions. (A) Mean cellular DinB-YPet concentrations inferred from fluorescence intensity measurements (Fig 3, main text; EAW643 cells) for untreated cells, and for cells treated for 3 h with 1 μg/mL trimethoprim-alone, 1 μg/mL trimethoprim + 2% DMSO, 30 ng/mL ciprofloxacin-alone and 30 ng/mL ciprofloxacin + 2% DMSO, plotted against the number of MuGam foci detected under similar treatment conditions (MG1655 carrying pEAW1162). (B) Mean number of DinB-YPet foci per cell (EAW643) for untreated cells, and for cells treated for 3 h with trimethoprim-alone, trimethoprim-DMSO, ciprofloxacin-alone and ciprofloxacin-DMSO, plotted against the number of MuGam foci detected under similar treatment conditions (MG1655 carrying pEAW1162).

Supplementary Figure S15. Number of pol IV foci per cell in *lexA*(Def) cells following ciprofloxacin or trimethoprim treatment under normal conditions or ROS-mitigating conditions. *lexA*(Def) cells were EAW1141. *lexA*(Def) Δ*recB* cells were EAW1144. (A) Upper row: Average projection in time (100 ms x 10 frames) showing DinB-YPet (pol IV) foci. Bottom row: Discoidal filtered projections. Cells were treated for 60 min prior to imaging. (B) Percentage of cells containing pol IV foci: 0 foci (light grey), 1 focus (grey), 2 foci (amber) and

≥ 3 foci (red). Cells were treated with 30 ng/mL ciprofloxacin (*n* = 106), 30 ng/mL ciprofloxacin + 2% DMSO (*n* = 109), 30 ng/mL ciprofloxacin in Δ*recB* (*n* = 106), 1 μg/mL trimethoprim (*n* = 145), 1 μg/mL trimethoprim + 2% DMSO (*n* = 102), 1 μg/mL trimethoprim in Δ*recB* (*n* = 94) experienced no damage for wild-type (*n* = 85) and Δ*recB* (*n* = 99). (C) Number of DinB-YPet foci per cell. Error bars represent standard error of the mean. Number of cells included in analysis: *n*(ciprofloxacin) = 106, *n*(ciprofloxacin-DMSO) = 109, *n*(ciprofloxacin in Δ*recB*) = 106, *n*(trimethoprim) = 145, *n*(trimethoprim-DMSO) = 102, *n*(trimethoprim in Δ*recB*) = 94, *n*(untreated $recB^+$) = 85, *n*(untreated Δ*recB*) = 99. * for $p < 0.05$; ** for $p < 0.01$ in two-sample t test for differences of means.

Supplementary Figure S16. Comparison of *recA*(E38K)-dependent focus formation in catalytically competent and catalytically dead pol IV fusions. (**A**) Images of DinB-YPet signals in *lexA*(Def) *recA*(E38K) cells (RW1598). (left panel) Average projection of 6 × 50 ms frames. (right panel) Average projection of 6 × 50 ms frames, where each from was subject to spatial filtering prior to projection [50]. (**B**) Images of DinB(D103N)-YPet signals in $lexA^+$ *recA*(E38K) cells (AR023). (left panel) Average projection of 6 × 50 ms frames. (right panel) Average projection of 6 × 50 ms frames, where each from was subject to spatial filtering prior to projection [50]. (**C**) Number of DinB-YPet foci per cell. Error bars represent standard error of the mean. Number of cells included in analysis: *n*(DinB-YPet) = 99, *n*(DinB[D103N]-YPet) = 175.

Supplementary Figure S17. Sensorgrams showing RecA(E38K) filament assembly on ssDNA and dsDNA in order to probe interactions with pol IV. (A) Sensorgram showing the immobilisation of ssDNA, $(dT)_{71}$, on the SA chip surface (association: dark grey phase; immobilised ssDNA: light grey phase). (B) Following ssDNA immobilisation, buffer containing 1 μM RecA(E38K) (+ 1 mM ATPγS) was flowed into the flow cell, at t = 0 min for 400 s. During this period, RecA(E38K) associated with ssDNA (blue phase), forming a RecA(E38K) filament.

At 400 s, buffer containing 1 mM ATPγS was flowed into the flow cell. RecA(E38K) dissociates from the surface (yellow phase). From 1,400 s, RU units are constant, consistent with stabilised RecA(E38K) filaments. (C) Sensorgram showing the immobilisation of dsDNA on the SA chip surface (association: dark grey phase; immobilised dsDNA: light grey phase). (D) Following dsDNA immobilisation, buffer containing 1 μM RecA(E38K) (+ 0.5 mM ATPγS) was flowed into the flow cell, at t = 0 min for 500 s. During this period, RecA(E38K) associated with dsDNA (blue phase), forming a RecA(E38K) filament. From 500 – 3,000 s, buffer containing 0.5 or 1 mM ATPγS was flowed into the flow cell (yellow phase). From 3,000 – 7,200 s, buffer containing 1 μM RecA(E38K) (+ 0.5 mM ATPγS) was flowed into the flow cell to allow for more RecA(E38K) to associate with the dsDNA. (E) Sensorgram showing the association of pol IV with RecA(E38K) structures formed on dsDNA. At t = 0 s, 0.65 uM pol IV (+ 0.5 mM ATPγS) was flowed into the flow cell for 220 s and association of pol IV was observed (green phase). At t = 220 s, buffer containing 0.5 mM ATPγS was flowed into the flow cell (yellow phase). (F) Sensorgram showing the association of pol IV with dsDNA. At t = 0 s, 0.65 uM pol IV (+ 0.5 mM ATPγS) was flowed into the flow cell for 220 s and association of pol IV was observed (green phase). At t = 220 s, buffer containing 0.5 mM ATPγS was flowed into the flow cell (yellow phase). Lower response units are recorded than for the association of pol IV with RecA(E38K) structures on dsDNA.

Supplementary Figure S18. RecA(E38K) forms RecA*-like structures on circular dsDNA. (A) RecA(E38K) readily binds to dsDNA. In six separate reactions, either RecA(E38K) or wild-type RecA was incubated at 37ºC with nicked circular dsDNA (cdsDNA), ATP, and an ATP regeneration system. (B) LexA Cleavage Assays. Reaction mixtures contained 40 mM Tris-HCl at pH 8.0, 10 mM $MgCl_2$, 30 mM NaCl, 2 mM dithiothreitol, 3 μM circular single-stranded DNA (cssDNA) or nicked circular double-stranded DNA (cdsDNA), 3 mM ATPγS, LexA, and RecA as noted. Reactions were incubated at 37˚C for 10 minutes before addition of UmuD or LexA. The reaction products were separated and visualized by 15% SDS-PAGE stained with Coomassie blue. Lane 1 contains a protein ladder while subsequent groups of three lanes

contain the same reaction mixture sampled at 0, 20, and 40 minutes. On cssDNA, RecA(E38K) and wild-type RecA form RecA* structures. On cdsDNA however, RecA(E38K) forms RecA*-like structures in contrast to wildtype RecA.

Supplementary Figure S19. UmuC concentration and activity following ciprofloxacin or trimethoprim treatment under normal conditions or ROS-scavenging conditions. All measurements used EAW282 cells. (A) Images showing UmuC-mKate2 (pol V) signal at 0, 90 and 180 min (left to right) for 30 ng/mL ciprofloxacin-alone, 30 ng/mL ciprofloxacin + 2% DMSO treatment or 1 μg/mL trimethoprim-alone treatment (top to bottom). Scale bar represents 5 μm. (B) Merged images showing UmuC-mKate2 (pol V) foci in magenta and τ-YPet (replisome) foci in magenta at 0, 90 and 180 min (left to right). Colocalized foci would appear as white foci. Scale bar represents 5 μm. (C) Concentration of UmuC-mKate2 during stress. Mean cell brightness is plotted against time (ciprofloxacin-alone: dark grey line, ciprofloxacin-DMSO: light grey line, trimethoprim-alone: magenta line). At each time-point, data are derived from >100 cells. Grey shaded error bands represent standard error of the mean. (D) Colocalization measurements following ciprofloxacin-alone treatment over 180 min: percentage of UmuC foci that are bound at replisomes (magenta line), percentage of replisomes that contain a UmuC focus (green line). Grey shaded error bands represent the standard error of the mean from three biological replicates together. Measurements are from >100 cells per time point. (E) Colocalization measurements following ciprofloxacin-DMSO treatment over 180 min: percentage of UmuC foci that are bound at replisomes (magenta line), percentage of replisomes that contain a UmuC focus (green line). Grey shaded error bands represent the standard error of the mean from three biological replicates together. Measurements are from >100 cells per time point.

Supplementary Figure S20. Western blots with anti-UmuD antibodies measuring levels of UmuD'. For each lane, 30 μL of lysate were loaded from cultures at OD_{600} 0.5. All strains used are Δ*umuDC* expressing UmuDC from a low-copy number plasmid (pRW154). After treatment, time points were taken at 1, 2, 3 h. (A) Western blot of *recA*$^+$ *lexA*$^+$ cells (RW120):

untreated, treated with 30 ng/mL ciprofloxacin or 30 ng/mL ciprofloxacin + 2% DMSO. (B) Western blot of *recA*$^{+}$ *lexA*$^{+}$ cells: untreated, treated with 1 μg/mL trimethoprim or 1 μg/mL trimethoprim + 2% DMSO. (*C*) Western blot of *recA*$^{+}$ *lexA51*(Def) cells (RW546): untreated, treated with ciprofloxacin or ciprofloxacin + 2% DMSO. (*D*) Western blot of *recA*$^{+}$ *lexA51*(Def) cells: untreated, treated with trimethoprim or trimethoprim + 2% DMSO.

Supplementary Movie S1. Time-lapse movie of Δ*recB* cells carrying P$_{sulA}$-*gfp*. Ciprofloxacin (30 ng/mL) was added to the media ay t = 0 min. An image was taken every 10 min over the period of 3 h. Upper movie: bright-field, bottom movie: signal from GFP expression (level of SOS induction).

Supplementary Movie S2. Burst acquisition movies of DinB-YPet in *recB*$^{+}$ cells treated with 1 μg/mL trimethoprim (trimethoprim, ± DMSO) or 30 ng/mL ciprofloxacin (± DMSO), Δ*recB* cells treated with trimethoprim or ciprofloxacin. Movies were recorded 60 min after antibiotic addition. Frames were taken every 0.1 s.

Supplementary Notes

Sequence of pJMuvrA-PAmCherry-mCI vector:

AAGCTGGAAGATCTTCCCTGGCACGACAGGTTTCCCGACTGGAAAGCGGGCAGTGAGC
GCAACGCAATTAATGTGAGTTAGCTCACTCATTAGGCACCCCAGGCTTTACACTTTATGC
TTCCGGCTCGTATGTTGTGTGGAATTGTGAGCGGATAACAATTTCACACAGGAAACAGCT
ATGACCATGATTACGCCAAGCGCGCAATTAACCCTCACTAAAGGGAACAAAAGCTGGGT
ACCGGGCCCCCCCTCGAGGTCGACTTCCGGGAAACAAACCTGGCCAGACATTGTTACA
CAACACTCCGGGTAATGCATTCCAATACTGTATATTCATTCAGGTCAATTTGTGTCATAATT
AACCGTTTGTGATCGGATCCAGCACCATGCCACCGGGCAAAAAAGCGTTTAATCCGGGA

AAGCATATGGTGAGCAAGGGCGAGGAGGATAACATGGCCATCATTAAGGAGTTCATGCG
CTTCAAGGTGCACATGGAGGGGTCCGTGAACGGCCACGTGTTCGAGATCGAGGGCGA
GGGCGAGGGCCGCCCCTACGAGGGCACCCAGACCGCCAAGCTGAAGGTGACCAAGG
GTGGCCCCCTGCCCTTCACCTGGGACATCCTGTCCCCTCAATTCATGTACGGCTCCAAT
GCCTACGTGAAGCACCCCGCCGACATCCCCGACTACTTTAAGCTGTCCTTCCCCGAGG
GCTTCAAGTGGGAGCGCGTGATGAAATTCGAGGACGGCGGCGTGGTGACCGTGACCC
AGGACTCCTCCCTGCAAGACGGTGAGTTCATCTACAAGGTGAAGCTGCGCGGCACCAA
CTTCCCCTCCGACGGCCCCGTAATGCAGAAGAAGACCATGGGCTGGGAGGCCCTCTCC
GAGCGGATGTACCCCGAGGACGGCGCCCTGAAGGGCGAGGTCAAGCCGCGCGTGAA
GCTGAAGGACGGCGGCCACTACGACGCTGAGGTCAAGACCACCTACAAGGCCAAGAA
GCCCGTGCAGCTGCCCGGCGCCTACAACGTCAACCGCAAGTTGGACATCACCTCACAC
AACGAGGACTACACCATCGTGGAACAGTACGAACGTGCCGAGGGCCGCCACTCCACCG
GCGGCATGGACGAGCTGTACAAGGAGCTCGCTGCAGGTGGCGGCGGCGGCTCCGGC
AGCCATATGTATGAGTACCCTGTTTTTCTCATGTTCAGGCAGGGATGTTCTCACCTGAG
CTTCGCACCTTTACCAAAGGTGATGCGGAGCGCTGGGTAAGCACAACCAAAAAAGCCA
GTGATTCTGCATTCTGGCTTGAGGTTGAAGGTAATTCCATGACCACACCAACAGGCTCC
AAGACAAGCTTTCCTGACGGAATGTTAATTCTCGTTGACCCTGAGCAGGCTGTTGAGCC
AGGTGATTTCTGCATTGCCCGCCTTGGGGGTGATGAGTTTACCTTCGCGAAACTGATCC
GCGATAGCGGTCAGGTGTTTTTACAACCACTGAACCCACAGTACCCAATGATCCCATGC
AATGAGAGTTGTTCCGTTGTGGGGAAAGTTATCGCTAGTCAGTGAGCGGCCGCGAATTC
GAAGTTCCTATAGTTTCTAGAGAATAGGAACTTCGATCTTTAGAAAAACTCATCGAGCATC
AAATGAAACTGCAATTTATTCATATCAGGATTATCAATACCATATTTTTGAAAAAGCCGTTTC
TGTAATGAAGGAGAAAACTCACCGAGGCAGTTCCATAGGATGGCAAGATCCTGGTATCG
GTCTGCGATTCCGACTCGTCCAACATCAATACAACCTATTAATTTCCCCTCGTCAAAAATA
AGGTTATCAAGTGAGAAATCACCATGAGTGACGACTGAATCCGGTGAGAATGGCAAAAG
CTTATGCATTTCTTTCCAGACTTGTTCAACAGGCCAGCCATTACGCTCGTCATCAAAATCA
CTCGCATCAACCAAACCGTTATTCATTCGTGATTGCGCCTGAGCGAGACGAAATACACG
ATCGCTGTTAAAAGGACAATTACAAACAGGAATCGAATGCAACCGGCGCAGGAACACTG

CCAGCGCATCAACAATATTTTCACCTGAATCAGGATATTCTTCTAATACCTGGAATGCTGTTTTCCCGGGGATCGCAGTGGTGAGTAACCATGCATCATCAGGAGTACGGATAAAATGCTTGATGGTCGGAAGAGGCATAAATTCCGTCAGCCAGTTTAGTCTGACCATCTCATCTGTAACATCATTGGCAACGCTACCTTTGCCATGTTTCAGAAACAACTCTGGCGCATCGGGCTTCCCATACAATCGATAGATTGTCGCACCTGATTGCCCGACATTATCGCGAGCCCATTTATACCCATATAAATCAGCATCCATGTTGGAATTTAATCGCGGGCGCGAGCAAGACGTTTCCCGTTGAATATGGCTCATAACACCCCTTGTATTACTGTTTATGTAAGCAGACAGTTTTATTGTTCATGATGATATATTTTTATCTTGTGCAATGTAACATCAGAGATTTTGAGACACAACGTGGCTTTCCCCGCCCGCCCGATCCCCGGGTACCGAGCTCGAATTTCGACCAATTCGAAGTTCCTATACTTTCTAGAGAATAGGAACTTCCCGCGGTGGAGCTCCAATTCGCCCTATAGTGAGTCGTATTACGCGCGCTCACTGGCCGTCGTTTTACAACGTCGTGACTGGGAAAACCCTGGCGTTACCCAACTTAATCGCCTTGCAGCACATCCCCCTTTCGCCAGCTGGCGTAATAGCGAAGAGGCCCGCACCGATCGCCCTTCCCAACAGTTGCGCAGCCTGAATGGCGAATGGGACGCGCCCTGTAGCGGCGCATTAAGCGCGGCGGGTGTGGTGGTTACGCGCAGCGTGACCGCTACACTTGCCAGCGCCCTAGCGCCCGCTCCTTTCGCTTTCTTCCCTTCCTTTCTCGCCACGTTCGCCGGAAGATCTTCCAATTCCCGACAGTAAGACGGGTAAGCCTGTTGATGATACCGCTGCCTTACTGGGTGCATTAGCCAGTCTGAATGACCTGTCACGGGATAATCCGAAGTGGTCAGACTGGAAAATCAGAGGGCAGGAACTGCTGAACAGCAAAAAGTCAGATAGCACCACATAGCAGACCCGCCATAAAACGCCCTGAGAAGCCCGTGACGGGCTTTTCTTGTATTATGGGTAGTTTCCTTGCATGAATCCATAAAAGGCGCCTGTAGTGCCATTTACCCCCATTCACTGCCAGAGCCGTGAGCGCAGCGAACTGAATGTCACGAAAAAGACAGCGACTCAGGTGCCTGATGGTCGGAGACAAAAGGAATATTCAGCGATTTGCCCGAGCTTGCGAGGGTGCTACTTAAGCCTTTAGGGTTTTAAGGTCTGTTTTGTAGAGGAGCAAACAGCGTTTGCGACATCCTTTTGTAATACTGCGGAACTGACTAAAGTAGTGAGTTATACACAGGGCTGGGATCTATTCTTTTTATCTTTTTTTATTCTTTCTTTATTCTATAAATTATAACCACTTGAATATAAACAAAAAAAACACACAAAGGTCTAGCGGAATTTACAGAGGGTCTAGCAGAATTTACAAGTTTTCCAGCAAAGGTCTAGCAGAATTTACAGATACCCACAACTCAAAGGAAAAGGACTAGTAATTATCATTGACTAGCCCATCTCAATTGGTATAGTGATTAAAATCACCTAGACCAATTGAGATGTATGTC

TGAATTAGTTGTTTTCAAAGCAAATGAACTAGCGATTAGTCGCTATGACTTAACGGAGCAT
GAAACCAAGCTAATTTTATGCTGTGTGGCACTACTCAACCCCACGATTGAAAACCCTACA
AGGAAAGAACGGACGGTATCGTTCACTTATAACCAATACGCTCAGATGATGAACATCAGT
AGGGAAAATGCTTATGGTGTATTAGCTAAAGCAACCAGAGAGCTGATGACGAGAACTGT
GGAAATCAGGAATCCTTTGGTTAAAGGCTTTGAGATTTTCCAGTGGACAAACTATGCCAA
GTTCTCAAGCGAAAAATTAGAATTAGTTTTTAGTGAAGAGATATTGCCTTATCTTTTCCAGT
TAAAAAAATTCATAAAATATAATCTGGAACATGTTAAGTCTTTTGAAAACAAATACTCTATGA
GGATTTATGAGTGGTTATTAAAAGAACTAACACAAAAGAAAACTCACAAGGCAAATATAGA
GATTAGCCTTGATGAATTTAAGTTCATGTTAATGCTTGAAAATAACTACCATGAGTTTAAAA
GGCTTAACCAATGGGTTTTGAAACCAATAAGTAAAGATTTAAACACTTACAGCAATATGAA
ATTGGTGGTTGATAAGCGAGGCCGCCCGACTGATACGTTGATTTTCCAAGTTGAACTAG
ATAGACAAATGGATCTCGTAACCGAACTTGAGAACAACCAGATAAAAATGAATGGTGACA
AAATACCAACAACCATTACATCAGATTCCTACCTACATAACGGACTAAGAAAAACACTACA
CGATGCTTTAACTGCAAAAATTCAGCTCACCAGTTTTGAGGCAAAATTTTTGAGTGACAT
GCAAAGTAAGTATGATCTCAATGGTTCGTTCTCATGGCTCACGCAAAAACAACGAACCAC
ACTAGAGAACATACTGGCTAAATACGGAAGGATCTGAGGTTCTTATGGCTCTTGTATCTAT
CAGTGAAGCATCAAGACTAACAAACAAAAGTAGAACAACTGTTCACCGTTACATATCAAA
GGGAAAACTGTCCATATATGCACAGATGAAAACGGTGTAAAAAAGATAGATACATCAGAG
CTTTTACGAGTTTTTGGTGCATTCAAAGCTGTTCACCATGAACAGATCGACAATGTAACA
GATGAACAGCATGTAACACCTAATAGAACAGGTGAAACCAGTAAAACAAAGCAACTAGAA
CATGAAATTGAACACCTGAGACAACTTGTTACAGCTCAACAGTCACACATAGACAGCCTG
AAACAGGCGATGCTGCTTATCGAATCAAAGCTGCCGACAACACGGGAGCCAGTGACGC
CTCCCGTGGGGAAAAAATCATGGCAATTCTGGAAGAAATAGCGCTTTCAGCCGGCAAAC
CTGAAGCCGGATCTGCGATTCTGATAACAAACTAGCAACACCAGAACAGCCCGTTTGCG
GGCAGCAAAACCCGTGGGAATTAATTCCCCTGCTCGCGCAGGCTGGGTGCCAAGCTCT
CGGGTAACATCAAGGCCCGATCCTTGGAGCCCTTGCCCTCCCGCACGATGATCGTGCC
GTGATCGAAATCCAGATCCTTGACCCGCAGTTGCAAACCCTCACTGATCCGCATGCCCG
TTCCATACAGAAGCTGGGCGAACAAACGATGCTCGCCTTCCAGAAAACCGAGGATGCG

AACCACTTCATCCGGGGTCAGCACCACCGGCAAGCGCCGCGACGGCCGAGGTCTTCC
GATCTCCTGAAGCCAGGGCAGATCCGTGCACAGCACCTTGCCGTAGAAGAACAGCAAG
GCCGCCAATGCCTGACGATGCGTGGAGACCGAAACCTTGCGCTCGTTCGCCAGCCAG
GACAGAAATGCCTCGACTTCGCTGCTGCCCAAGGTTGCCGGGTGACGCACACCGTGG
AAACGGATGAAGGCACGAACCCAGTGGACATAAGCCTGTTCGGTTCGTAAGCTGTAATG
CAAGTAGCGTATGCGCTCACGCAACTGGTCCAGAACCTTGACCGAACGCAGCGGTGGT
AACGGCGCAGTGGCGGTTTTCATGGCTTGTTATGACTGTTTTTTTGGGGTACAGTCTATG
CCTCGGGCATCCAAGCAGCAAGCGCGTTACGCCGTGGGTCGATGTTTGATGTTATGGA
GCAGCAACGATGTTACGCAGCAGGGCAGTCGCCCTAAAACAAAGTTAAACATCATGAGG
GAAGCGGTGATCGCCGAAGTATCGACTCAACTATCAGAGGTAGTTGGCGTCATCGAGCG
CCATCTCGAACCGACGTTGCTGGCCGTACATTTGTACGGCTCCGCAGTGGATGGCGGC
CTGAAGCCACACAGTGATATTGATTTGCTGGTTACGGTGACCGTAAGGCTTGATGAAACA
ACGCGGCGAGCTTTGATCAACGACCTTTTGGAAACTTCGGCTTCCCCTGGAGAGAGCG
AGATTCTCCGCGCTGTAGAAGTCACCATTGTTGTGCACGACGACATCATTCCGTGGCGT
TATCCAGCTAAGCGCGAACTGCAATTTGGAGAATGGCAGCGCAATGACATTCTTGCAGG
TATCTTCGAGCCAGCCACGATCGACATTGATCTGGCTATCTTGCTGACAAAAGCAAGAGA
ACATAGCGTTGCCTTGGTAGGTCCAGCGGCGGAGGAACTCTTTGATCCGGTTCCTGAA
CAGGATCTATTTGAGGCGCTAAATGAAACCTTAACGCTATGGAACTCGCCGCCCGACTG
GGCTGGCGATGAGCGAAATGTAGTGCTTACGTTGTCCCGCATTTGGTACAGCGCAGTAA
CCGGCAAAATCGCGCCGAAGGATGTCGCTGCCGACTGGGCAATGGAGCGCCTGCCGG
CCCAGTATCAGCCCGTCATACTTGAAGCTAGACAGGCTTATCTTGGACAAGAAGAAGATC
GCTTGGCCTCGCGCGCAGATCAGTTGGAAGAATTTGTCCACTACGTGAAAGGCGAGAT
CACCAAGGTAGTCGGCAAATAATGTCTAACAATTCGTTCAAGCCGACGCCGCTTCGCGG
CGCGGCTTAACTCAAGCGTTAGATGCACTAAGCACATAATTGCTCACAGCCAAACTATCA
GGTCAAGTCTGCTTTTATTATTTTTAAGCGTGCATAATAAGCCCTACACAAATTGGGAGAT
ATATCATGAAAGGCTGGCTTTTTCTTGTTATCGCAATAGTTGGCGAAGTAATCGCAACATC
CGCATTAAAATCTAGCGAGGGCTTTACT

Sequence of pBAD-MuGam-PAmCherry vector (pEAW1162):

AAGAAACCAATTGTCCATATTGCATCAGACATTGCCGTCACTGCGTCTTTTACTGGCTCT
TCTCGCTAACCAAACCGGTAACCCCGCTTATTAAAAGCATTCTGTAACAAAGCGGGACC
AAAGCCATGACAAAAACGCGTAACAAAAGTGTCTATAATCACGGCAGAAAAGTCCACAT
TGATTATTTGCACGGCGTCACACTTTGCTATGCCATAGCATTTTTATCCATAAGATTAGC
GGATCCTACCTGACGCTTTTTATCGCAACTCTCTACTGTTTCTCCATACCCGTTTTTTGG
GCTAACAGGAGGAATTAACATATGGCTAAACCAGCAAAACGTATCAAGAGTGCCGCAG
CGGCTTATGTGCCACAAAACCGCGATGCGGTGATTACCGATATTAAACGCATCGGGGA
TTTACAGCGCGAAGCATCACGTCTGGAAACGGAAATGAATGATGCCATCGCGGAAATTA
CGGAGAAATTTGCGGCCCGGATTGCACCGATTAAAACCGATATTGAAACCCTTTCAAAA
GGCGTTCAGGGATGGTGTGAAGCGAACCGCGACGAACTGACGAACGGCGGCAAAGTG
AAGACGGCGAATCTTGTCACCGGTGATGTATCGTGGCGGGTCCGTCCACCATCAGTAA
GTATTCGTGGTATGGATGCAGTGATGGAAACGCTGGAGCGTCTTGGCCTGCAACGCTT
TATTCGCACGAAGCAGGAAATCAACAAGGAAGCGATTTTACTGGAACCGAAAGCGGTC
GCAGGCGTTGCCGGAATTACAGTTAAATCAGGCATTGAGGATTTTTCTATTATTCCATTT
GAACAGGAAGCCGGTATTTCCGCTGGCTCCGCTGCTGGTTCTGGCGAATTCATGGTGA
GCAAGGGCGAGGAGGATAACATGGCCATCATTAAGGAGTTCATGCGCTTCAAGGTGCA
CATGGAGGGGTCCGTGAACGGCCACGTGTTCGAGATCGAGGGCGAGGGCGAGGGCC
GCCCCTACGAGGGCACCCAGACCGCCAAGCTGAAGGTGACCAAGGGTGGCCCCCTGC
CCTTCACCTGGGACATCCTGTCCCCTCAATTCATGTACGGCTCCAATGCCTACGTGAAG
CACCCCGCCGACATCCCCGACTACTTTAAGCTGTCCTTCCCCGAGGGCTTCAAGTGGG
AGCGCGTGATGAAATTCGAGGACGGCGGCGTGGTGACCGTGACCCAGGACTCCTCCC
TGCAGGACGGTGAGTTCATCTACAAGGTGAAGCTGCGCGGCACCAACTTCCCCTCCGA
CGGCCCCGTAATGCAGAAGAAGACCATGGGCTGGGAGGCCCTCTCCGAGCGGATGTA
CCCCGAGGACGGCGCCCTGAAGGGCGAGGTCAAGCCGAGAGTGAAGCTGAAGGACG
GCGGCCACTACGACGCTGAGGTCAAGACCACCTACAAGGCCAAGAAGCCCGTGCAGC
TGCCCGGCGCCTACAACGTCAACCGCAAGTTGGACATCACCTCACACAACGAGGACTA

CACCATCGTGGAACAGTACGAACGTGCCGAGGGCCGCCACTCCACCGGCGGCATGGA
CGAGCTGTACAAGTAAAAGCTTGGGCCCGAACAAAAACTCATCTCAGAAGAGGATCTG
AATAGCGCCGTCGACCATCATCATCATCATCATTGAGTTTAAACGGTCTCCAGCTTGGC
TGTTTTGGCGGATGAGAGAAGATTTTCAGCCTGATACAGATTAAATCAGAACGCAGAAG
CGGTCTGATAAAACAGAATTTGCCTGGCGGCAGTAGCGCGGTGGTCCCACCTGACCCC
ATGCCGAACTCAGAAGTGAAACGCCGTAGCGCCGATGGTAGTGTGGGGTCTCCCCATG
CGAGAGTAGGGAACTGCCAGGCATCAAATAAAACGAAAGGCTCAGTCGAAAGACTGGG
CCTTTCGTTTTATCTGTTGTTTGTCGGTGAACGCTCTCCTGAGTAGGACAAATCCGCCG
GGAGCGGATTTGAACGTTGCGAAGCAACGGCCCGGAGGGTGGCGGGCAGGACGCCC
GCCATAAACTGCCAGGCATCAAATTAAGCAGAAGGCCATCCTGACGGATGGCCTTTTTG
CGTTTCTACAAACTCTTTTGTTTATTTTTCTAAATACATTCAAATATGTATCCGCTCATGA
GACAATAACCCTGATAAATGCTTCAATAATATTGAAAAAGGAAGAGTATGAGTATTCAAC
ATTTCCGTGTCGCCCTTATTCCCTTTTTTGCGGCATTTTGCCTTCCTGTTTTTGCTCACC
CAGAAACGCTGGTGAAAGTAAAAGATGCTGAAGATCAGTTGGGTGCACGAGTGGGTTA
CATCGAACTGGATCTCAACAGCGGTAAGATCCTTGAGAGTTTTCGCCCCGAAGAACGTT
TTCCAATGATGAGCACTTTTAAAGTTCTGCTATGTGGCGCGGTATTATCCCGTGTTGAC
GCCGGGCAAGAGCAACTCGGTCGCCGCATACACTATTCTCAGAATGACTTGGTTGAGT
ACTCACCAGTCACAGAAAAGCATCTTACGGATGGCATGACAGTAAGAGAATTATGCAGT
GCTGCCATAACCATGAGTGATAACACTGCGGCCAACTTACTTCTGACAACGATCGGAG
GACCGAAGGAGCTAACCGCTTTTTTGCACAACATGGGGGATCATGTAACTCGCCTTGAT
CGTTGGGAACCGGAGCTGAATGAAGCCATACCAAACGACGAGCGTGACACCACGATG
CCTGTAGCAATGGCAACAACGTTGCGCAAACTATTAACTGGCGAACTACTTACTCTAGC
TTCCCGGCAACAATTAATAGACTGGATGGAGGCGGATAAAGTTGCAGGACCACTTCTG
CGCTCGGCCCTTCCGGCTGGCTGGTTTATTGCTGATAAATCTGGAGCCGGTGAGCGTG
GGTCTCGCGGTATCATTGCAGCACTGGGGCCAGATGGTAAGCCCTCCCGTATCGTAGT
TATCTACACGACGGGGAGTCAGGCAACTATGGATGAACGAAATAGACAGATCGCTGAG
ATAGGTGCCTCACTGATTAAGCATTGGTAACTGTCAGACCAAGTTTACTCATATATACTT
TAGATTGATTTAAAACTTCATTTTTAATTTAAAAGGATCTAGGTGAAGATCCTTTTTGATA

ATCTCATGACCAAAATCCCTTAACGTGAGTTTTCGTTCCACTGAGCGTCAGACCCCGTAGAAAAGATCAAAGGATCTTCTTGAGATCCTTTTTTTCTGCGCGTAATCTGCTGCTTGCAAACAAAAAAACCACCGCTACCAGCGGTGGTTTGTTTGCCGGATCAAGAGCTACCAACTCTTTTTCCGAAGGTAACTGGCTTCAGCAGAGCGCAGATACCAAATACTGTCCTTCTAGTGTAGCCGTAGTTAGGCCACCACTTCAAGAACTCTGTAGCACCGCCTACATACCTCGCTCTGCTAATCCTGTTACCAGTGGCTGCTGCCAGTGGCGATAAGTCGTGTCTTACCGGGTTGGACTCAAGACGATAGTTACCGGATAAGGCGCAGCGGTCGGGCTGAACGGGGGGTTCGTGCACACAGCCCAGCTTGGAGCGAACGACCTACACCGAACTGAGATACCTACAGCGTGAGCTATGAGAAAGCGCCACGCTTCCCGAAGGGAGAAAGGCGGACAGGTATCCGGTAAGCGGCAGGGTCGGAACAGGAGAGCGCACGAGGGAGCTTCCAGGGGGAAACGCCTGGTATCTTTATAGTCCTGTCGGGTTTCGCCACCTCTGACTTGAGCGTCGATTTTTGTGATGCTCGTCAGGGGGGCGGAGCCTATGGAAAAACGCCAGCAACGCGGCCTTTTTACGGTTCCTGGCCTTTTGCTGGCCTTTTGCTCACATGTTCTTTCCTGCGTTATCCCCTGATTCTGTGGATAACCGTATTACCGCCTTTGAGTGAGCTGATACCGCTCGCCGCAGCCGAACGACCGAGCGCAGCGAGTCAGTGAGCGAGGAAGCGGAAGAGCGCCTGATGCGGTATTTTCTCCTTACGCATCTGTGCGGTATTTCACACCGCATAtaTGGTGCACTCTCAGTACAATCTGCTCTGATGCCGCATAGTTAAGCCAGTATACACTCCGCTATCGCTACGTGACTGGGTCATGGCTGCGCCCCGACACCCGCCAACACCCGCTGACGCGCCCTGACGGGCTTGTCTGCTCCCGGCATCCGCTTACAGACAAGCTGTGACCGTCTCCGGGAGCTGCATGTGTCAGAGGTTTTCACCGTCATCACCGAAACGCGCGAGGCAGCAGATCAATTCGCGCGCGAAGGCGAAGCGGCATGCATAATGTGCCTGTCAAATGGACGAAGCAGGGATTCTGCAAACCCTATGCTACTCCGTCAAGCCGTCAATTGTCTGATTCGTTACCAATTATGACAACTTGACGGCTACATCATTCACTTTTTCTTCACAACCGGCACGGAACTCGCTCGGGCTGGCCCCGGTGCATTTTTTAAATACCCGCGAGAAATAGAGTTGATCGTCAAAACCAACATTGCGACCGACGGTGGCGATAGGCATCCGGGTGGTGCTCAAAAGCAGCTTCGCCTGGCTGATACGTTGGTCCTCGCGCCAGCTTAAGACGCTAATCCCTAACTGCTGGCGGAAAAGATGTGACAGACGCGACGGCGACAAGCAAACATGCTGTGCGACGCTGGCGATATCAAAATTGCTGTCTGCCAGGTGATCGCTGATGTACTGACAAGCCTCGCGTACCCGATTATCCATCGGTGGATGGAGCGA

CTCGTTAATCGCTTCCATGCGCCGCAGTAACAATTGCTCAAGCAGATTTATCGCCAGCA
GCTCCGAATAGCGCCCTTCCCCTTGCCCGGCGTTAATGATTTGCCCAAACAGGTCGCT
GAAATGCGGCTGGTGCGCTTCATCCGGGCGAAAGAACCCCGTATTGGCAAATATTGAC
GGCCAGTTAAGCCATTCATGCCAGTAGGCGCGCGGACGAAAGTAAACCCACTGGTGAT
ACCATTCGCGAGCCTCCGGATGACGACCGTAGTGATGAATCTCTCCTGGCGGGAACAG
CAAAATATCACCCGGTCGGCAAACAAATTCTCGTCCCTGATTTTTCACCACCCCCTGAC
CGCGAATGGTGAGATTGAGAATATAACCTTTCATTCCCAGCGGTCGGTCGATAAAAAAA
TCGAGATAACCGTTGGCCTCAATCGGCGTTAAACCCGCCACCAGATGGGCATTAAACG
AGTATCCCGGCAGCAGGGGATCATTTTGCGCTTCAGCCATACTTTTCATACTCCCGCCA
TTCAGAG

Supplementary Figure S1

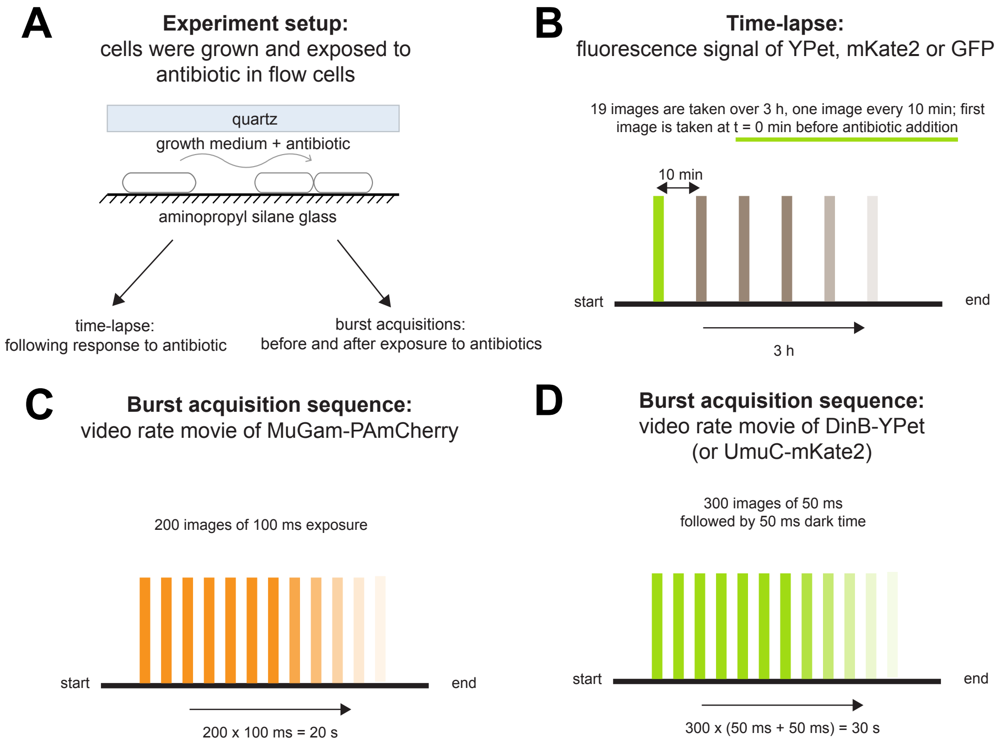

Supplementary Figure S2

A Survival plates for different MuGam-PAmCherry expression levels

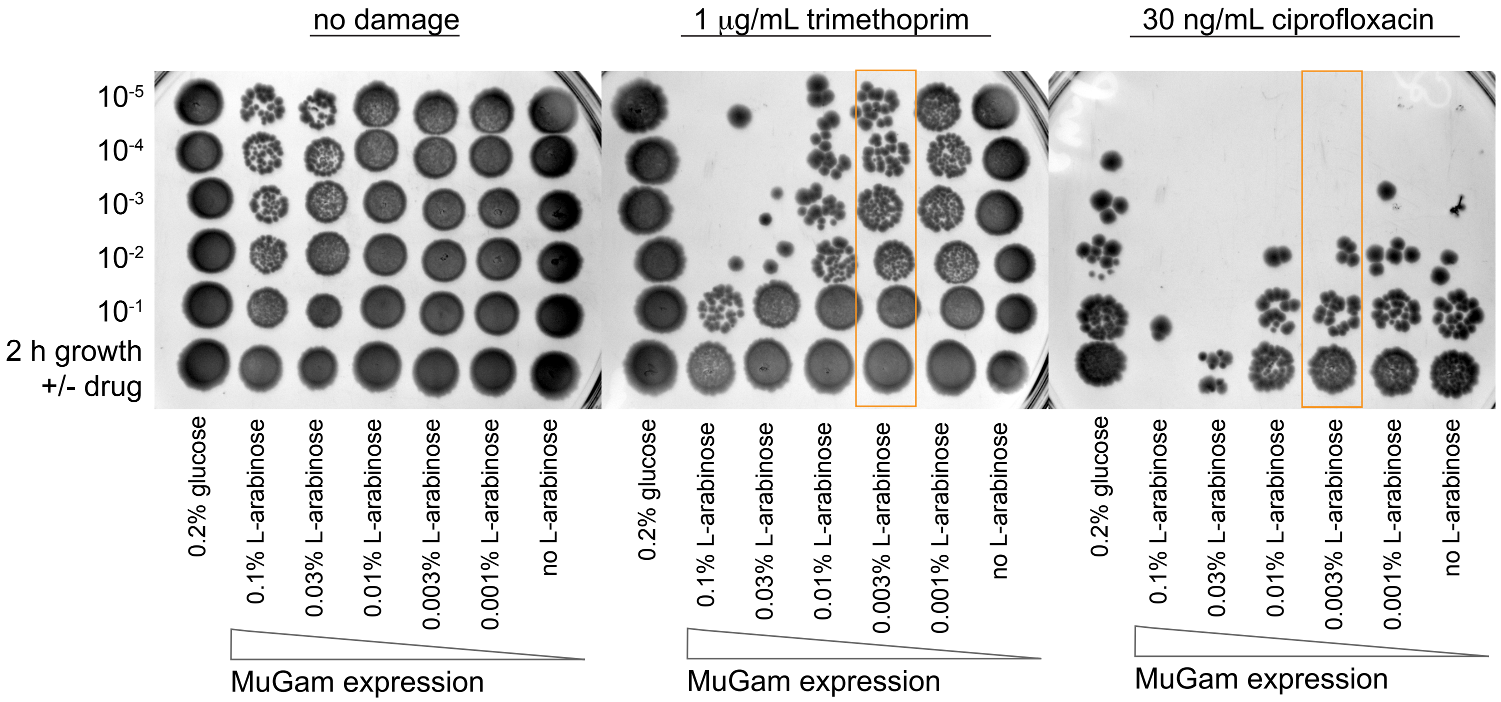

B MuGam-PAmCherry signal at 0.003% L-arabinose

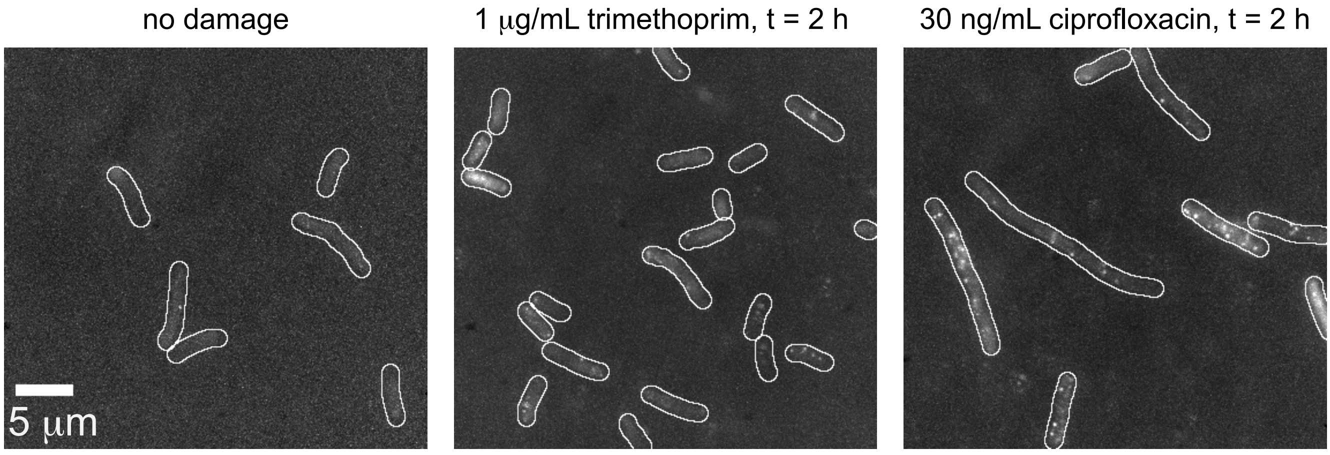

Supplementary Figure S3

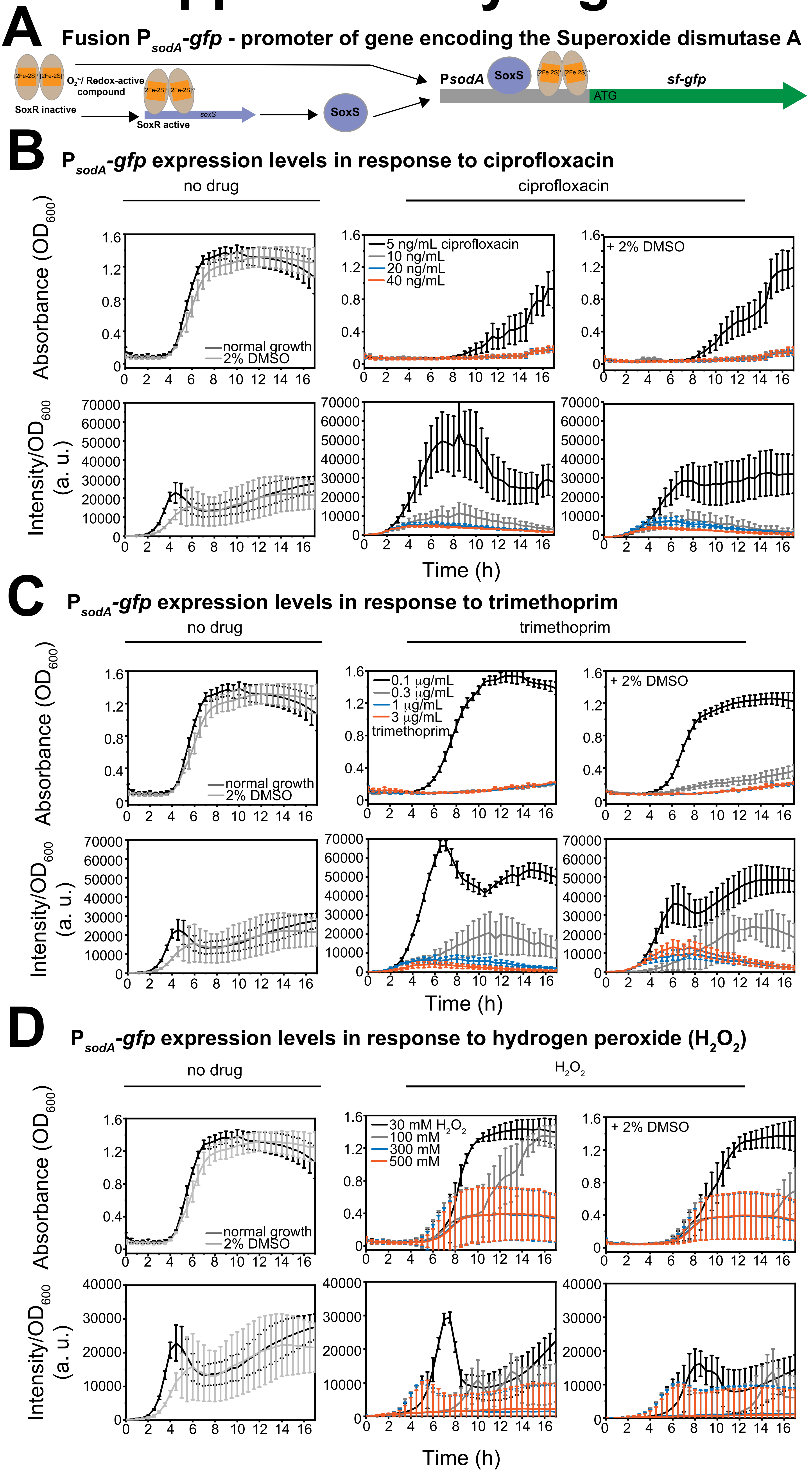

Supplementary Figure S4

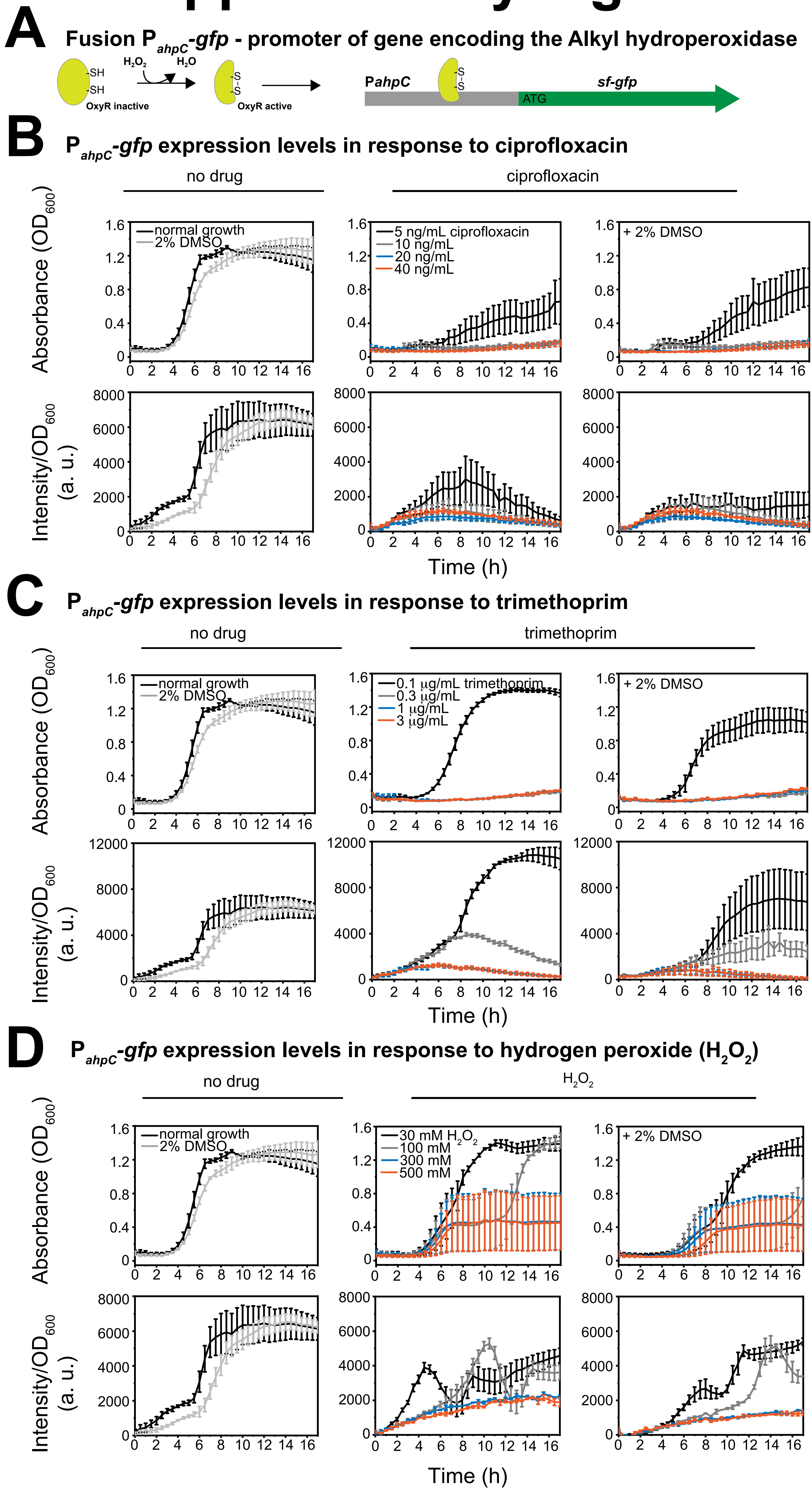

Supplementary Figure S5

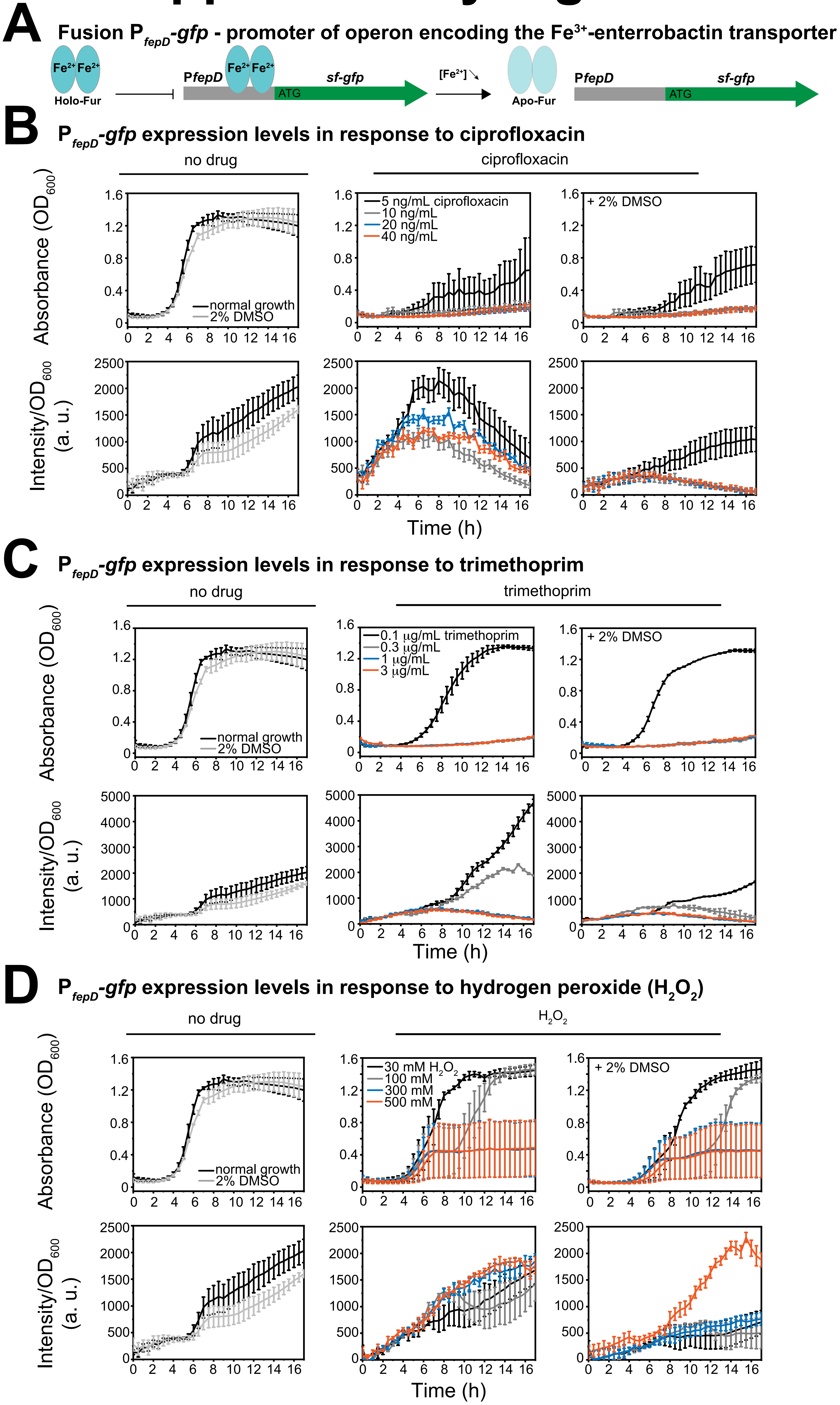

Supplementary Figure S6

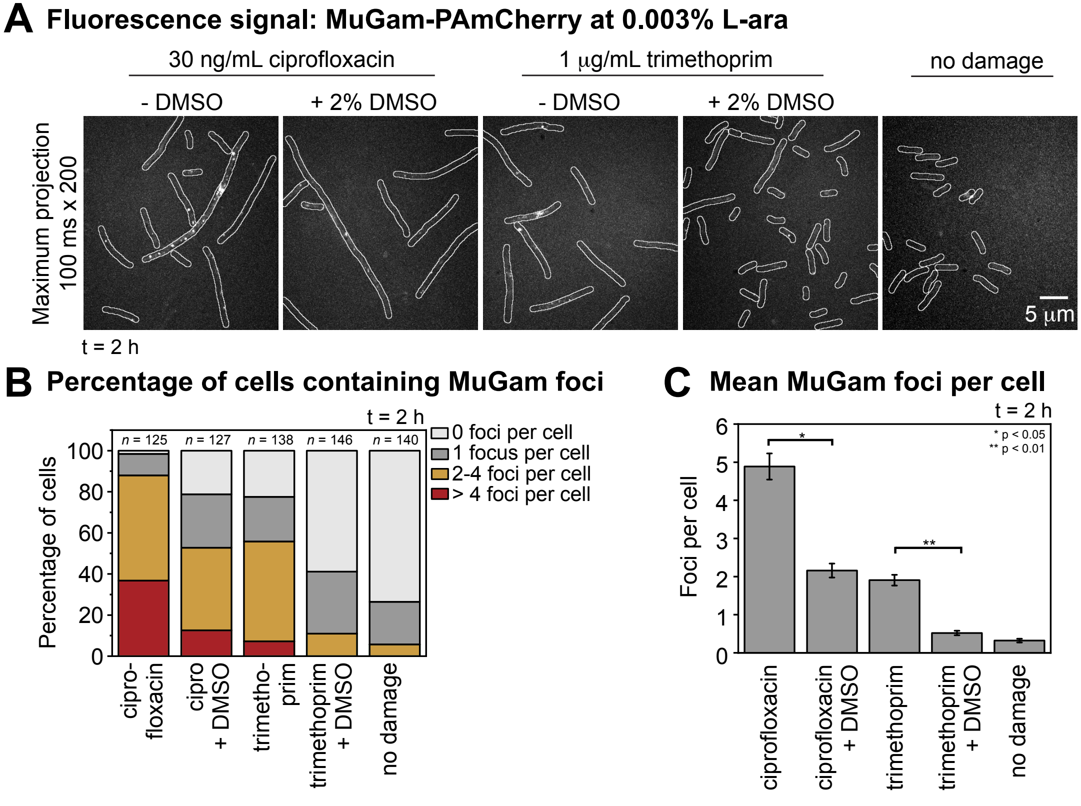

Supplementary Figure S7

A P_{sulA}-***gfp*** **expression levels in wild-type and Δ*recB* in response to ciprofloxacin**

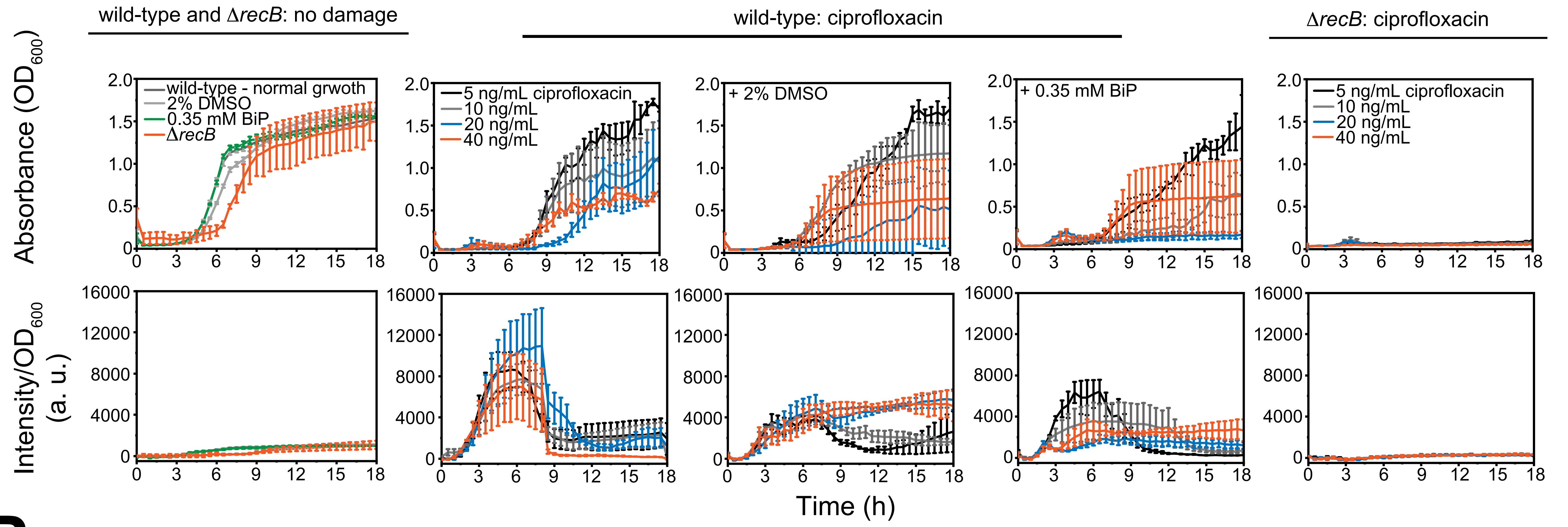

B P_{sulA}-***gfp*** **expression levels in wild-type and Δ*recB* in response to trimethoprim**

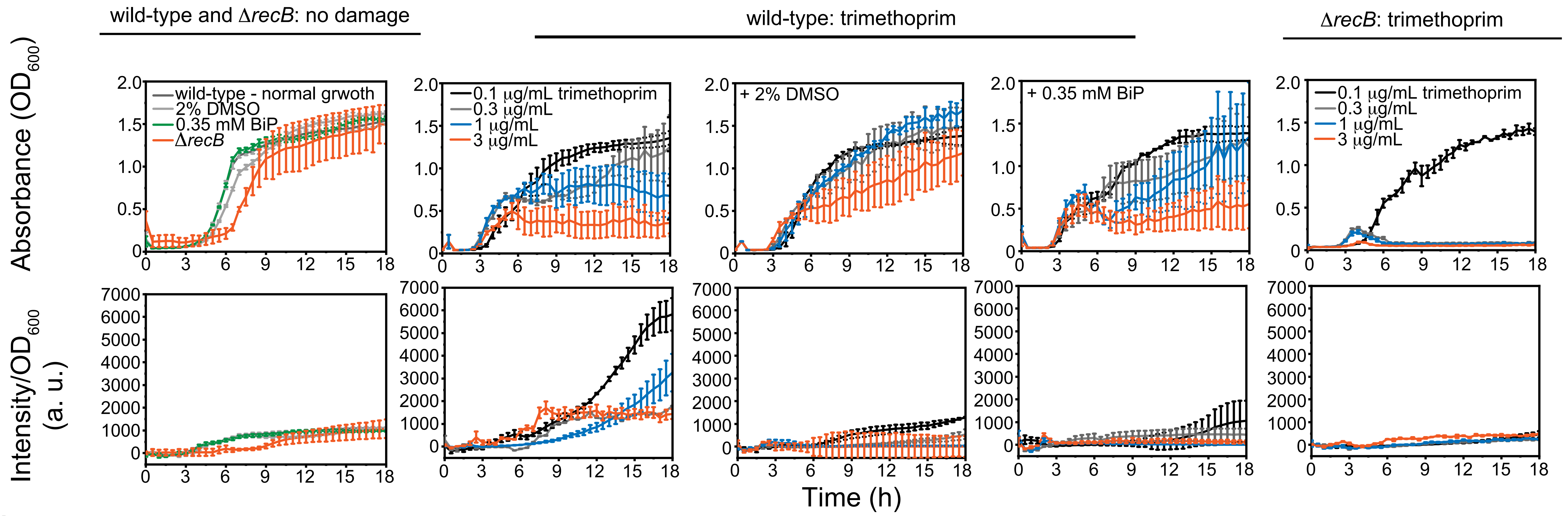

C P_{sulA}-***gfp*** **expression levels in wild-type cells in response to hydrogen peroxide (H_2O_2)**

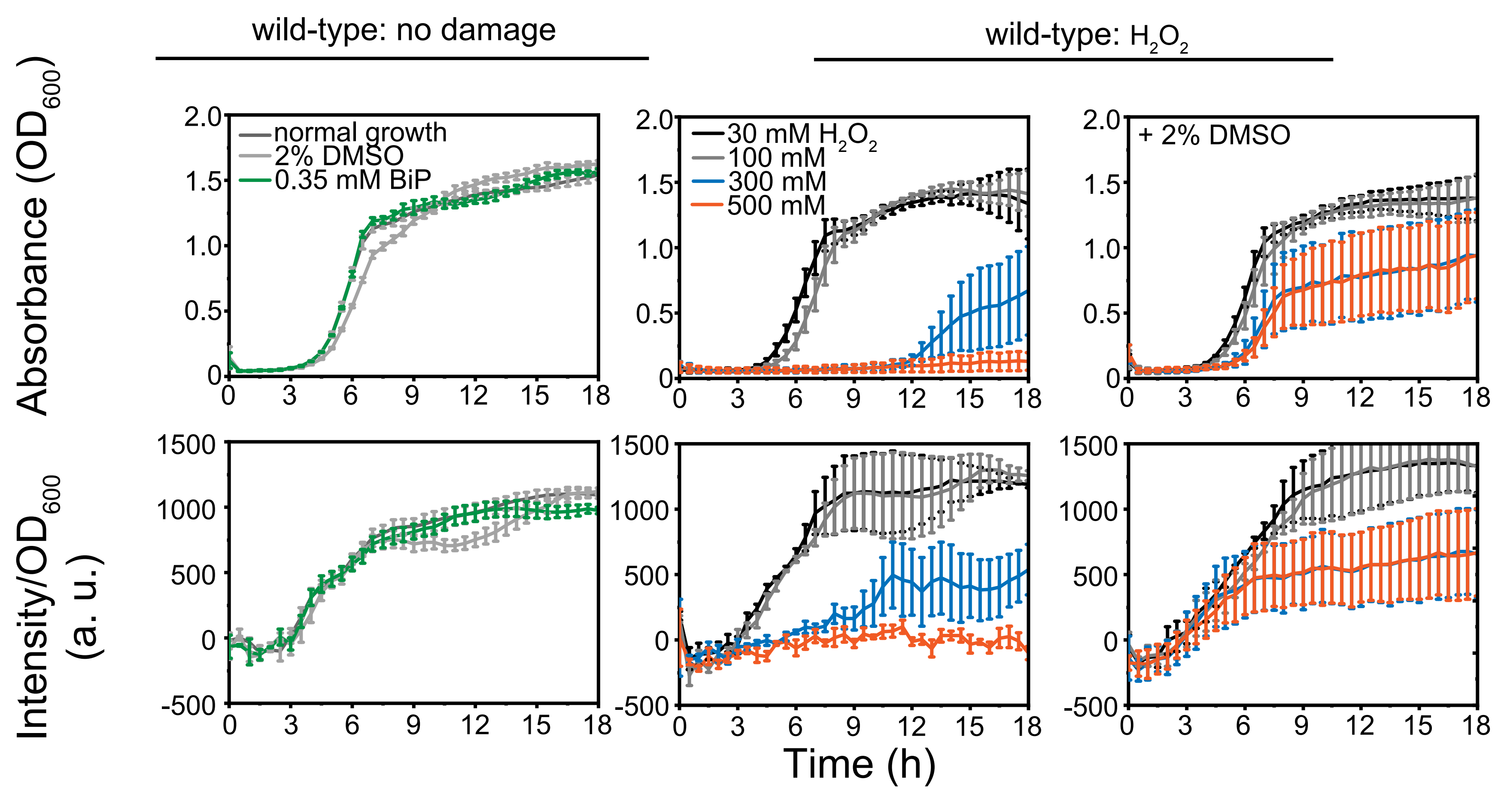

Supplementary Figure S8

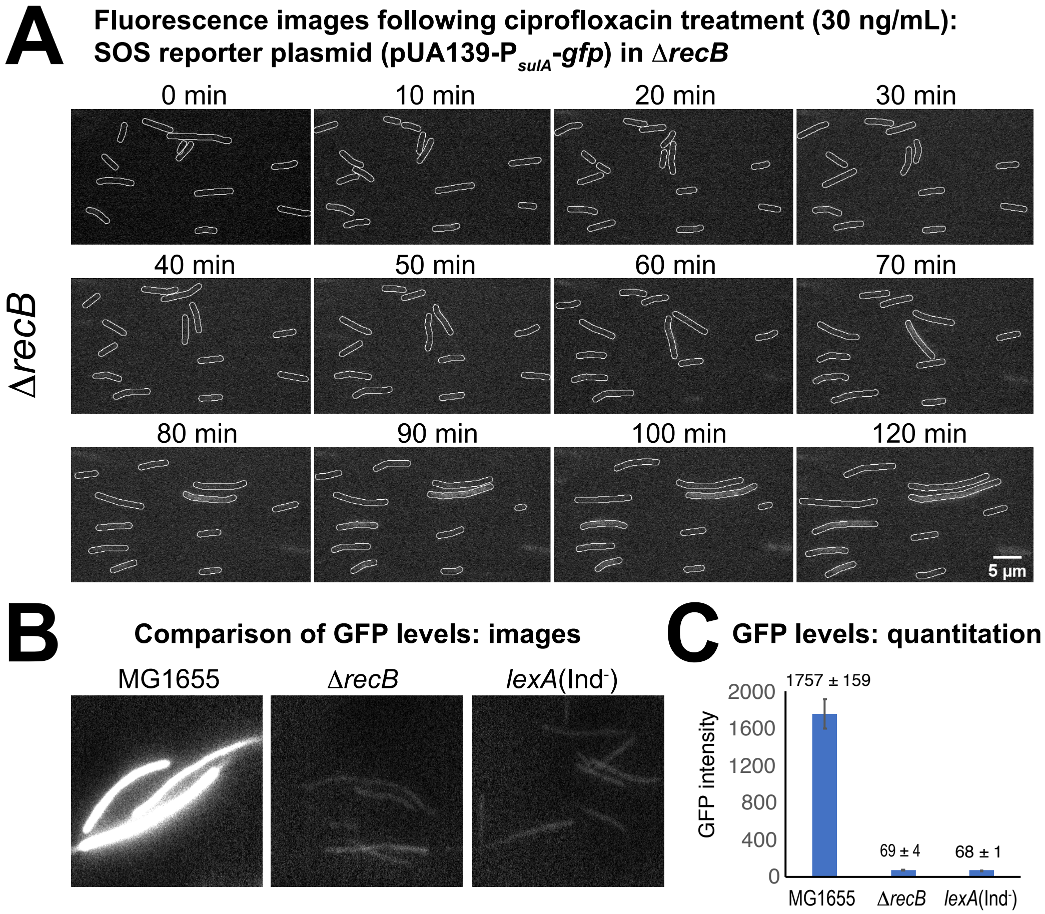

Supplementary Figure S9

A P_{sulA}-*gfp* expression levels in wild-type and Δ*recFOR* in response to ciprofloxacin

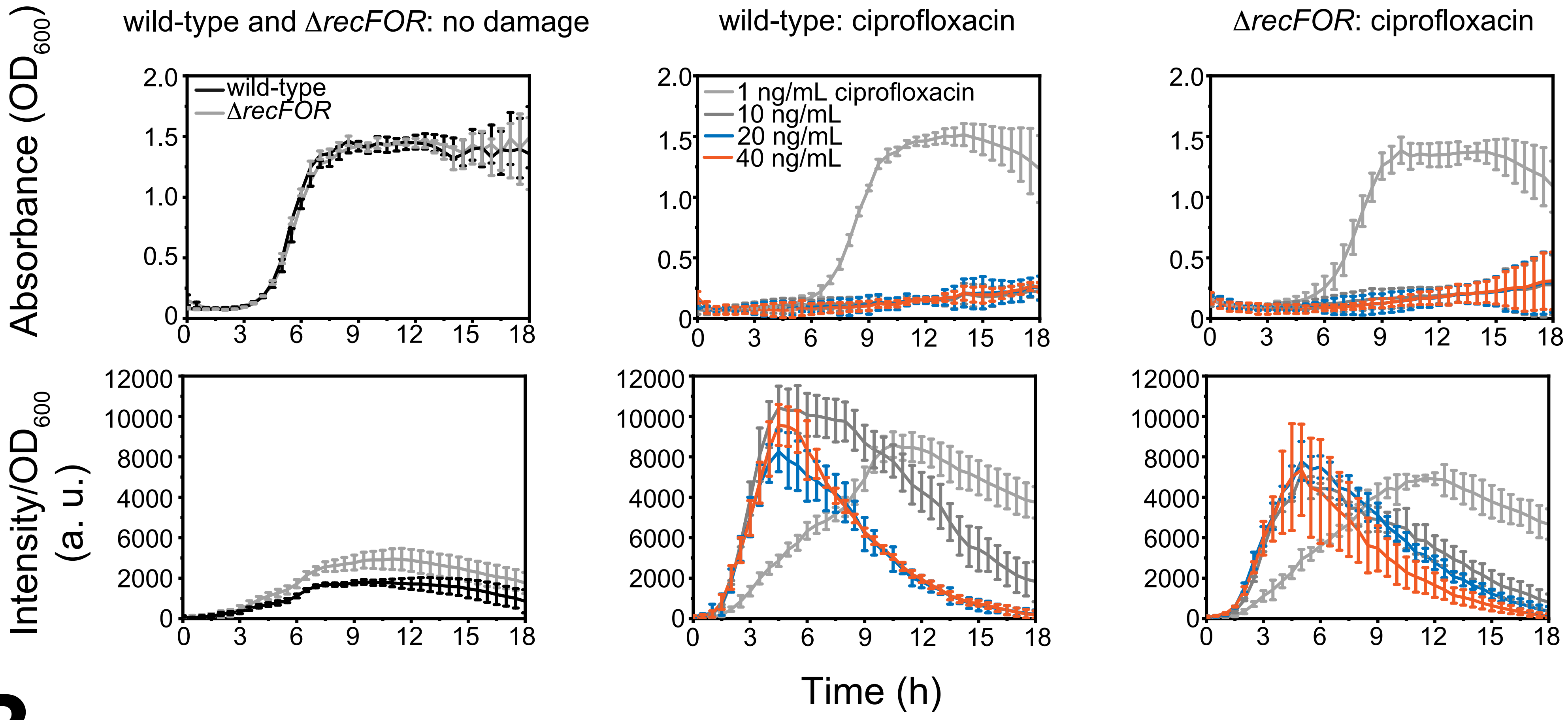

B P_{sulA}-*gfp* expression levels in wild-type and Δ*recFOR* in response to trimethoprim

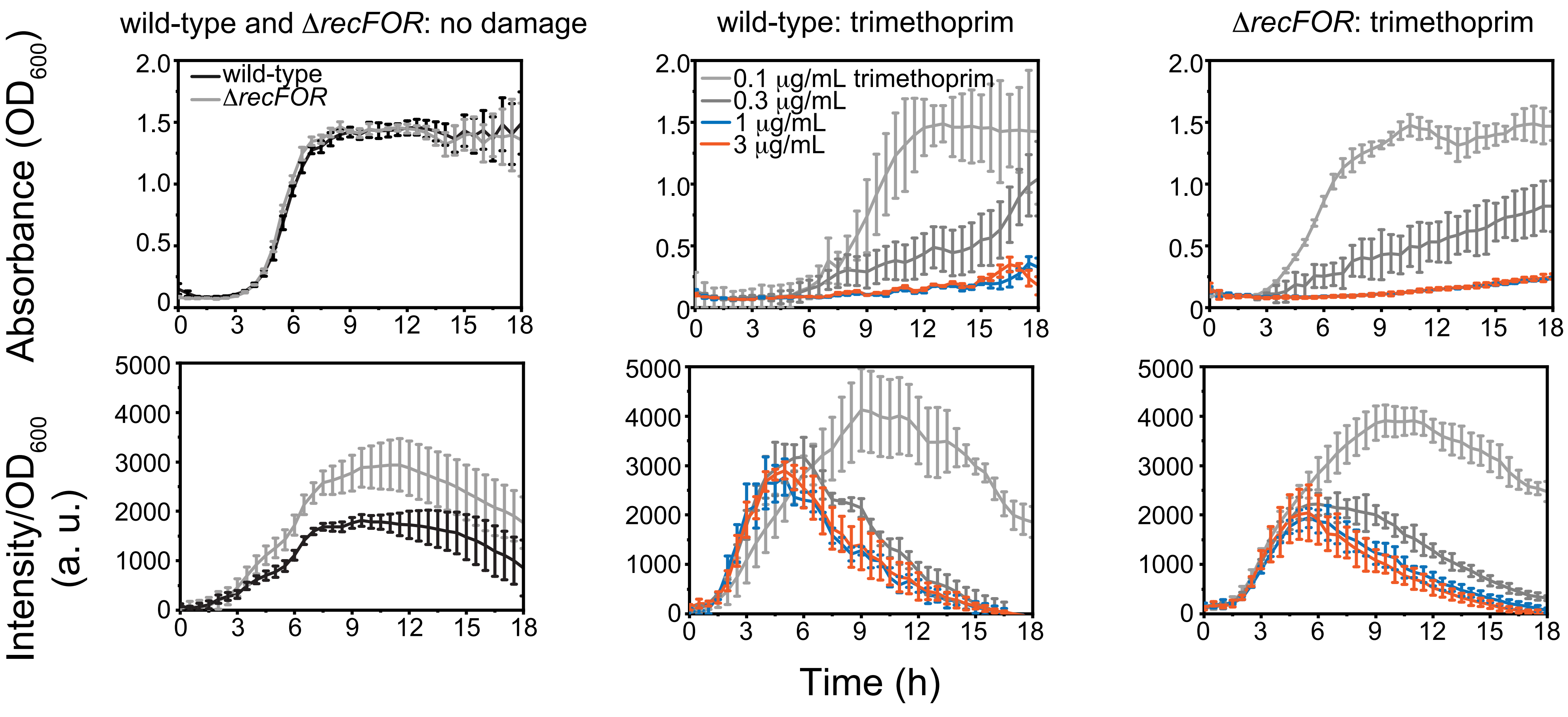

Supplementary Figure S10

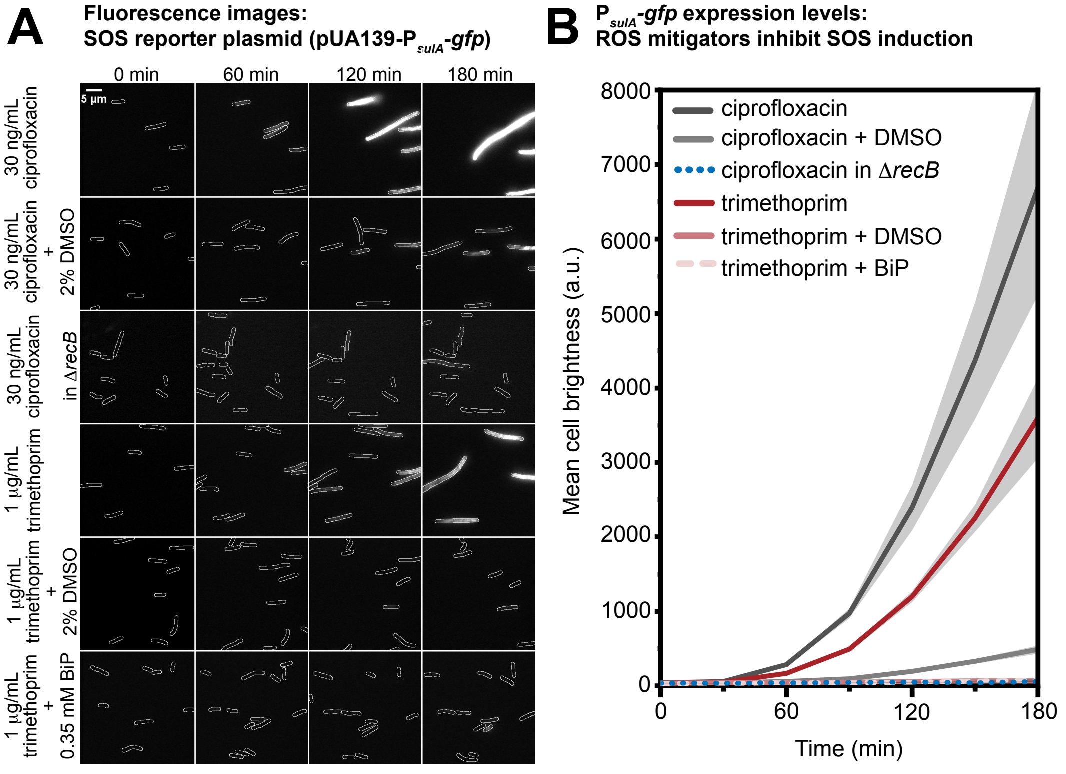

Supplementary Figure S11

A **Fluorescence images: *recA*[E38K] cells (constitutive SOS) carrying SOS reporter plasmid (pUA139-P$_{sulA}$-*gfp*)**

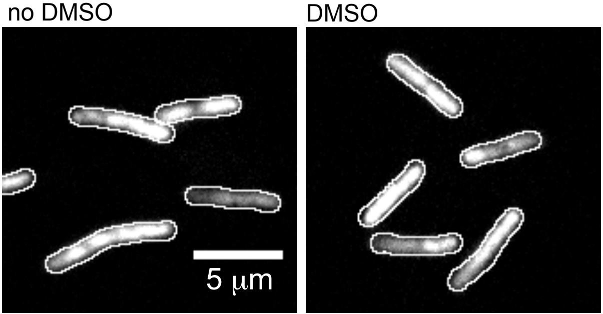

B **SulA expression levels: +/- DMSO (2%)**

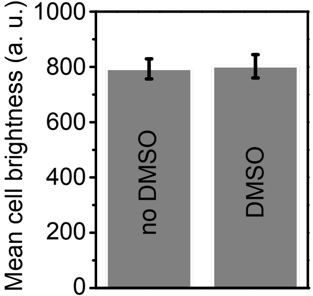

Supplementary Figure S12

Plate-based survival assay

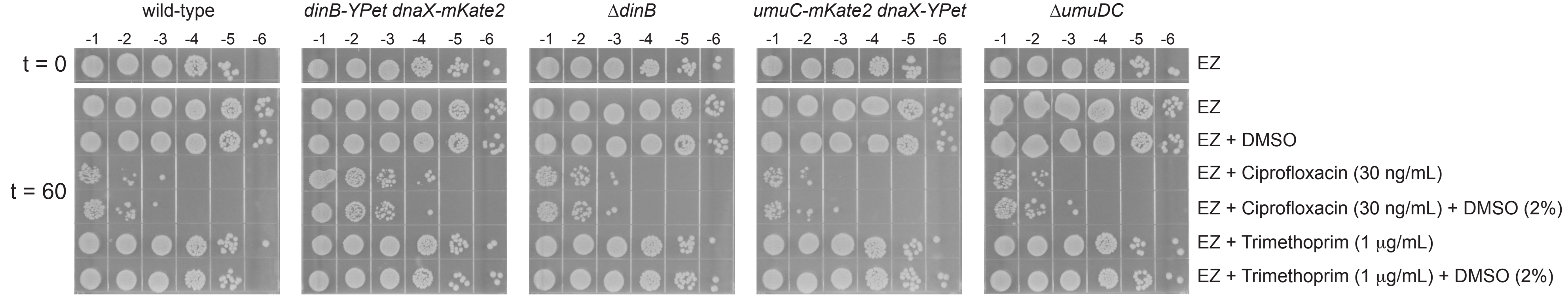

Supplementary Figure S13

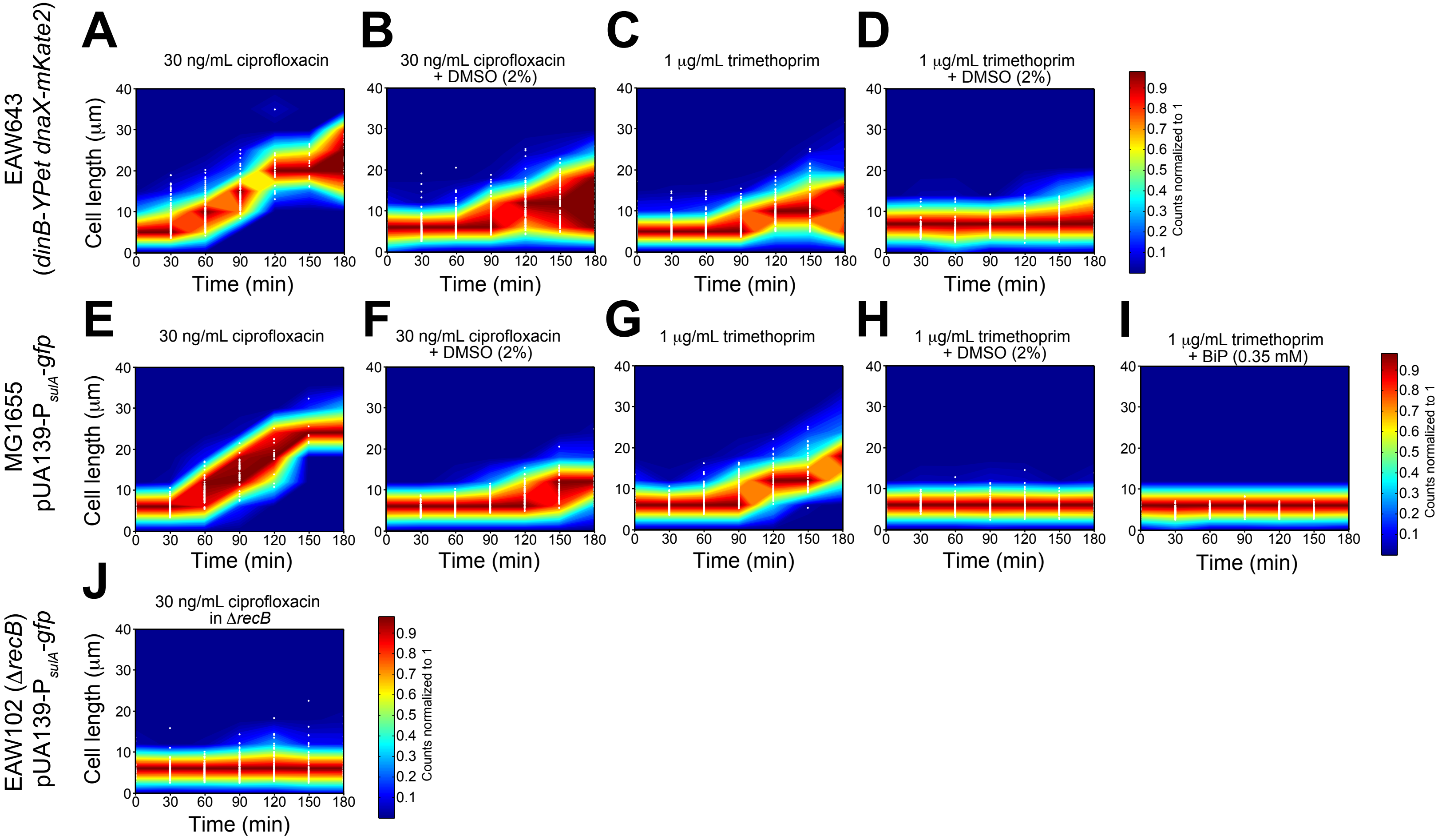

Supplemetary Figure S14

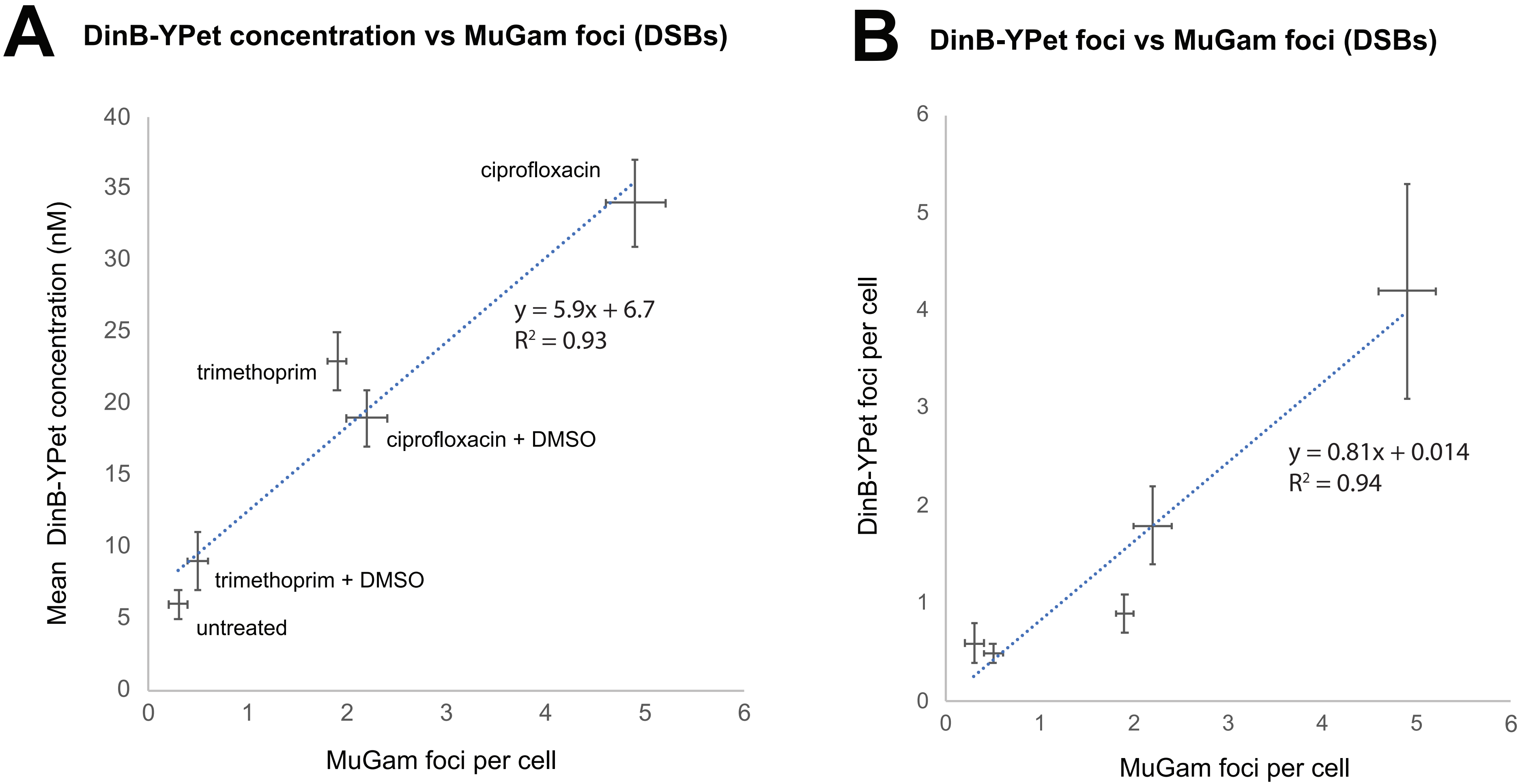

Supplementary Figure S15

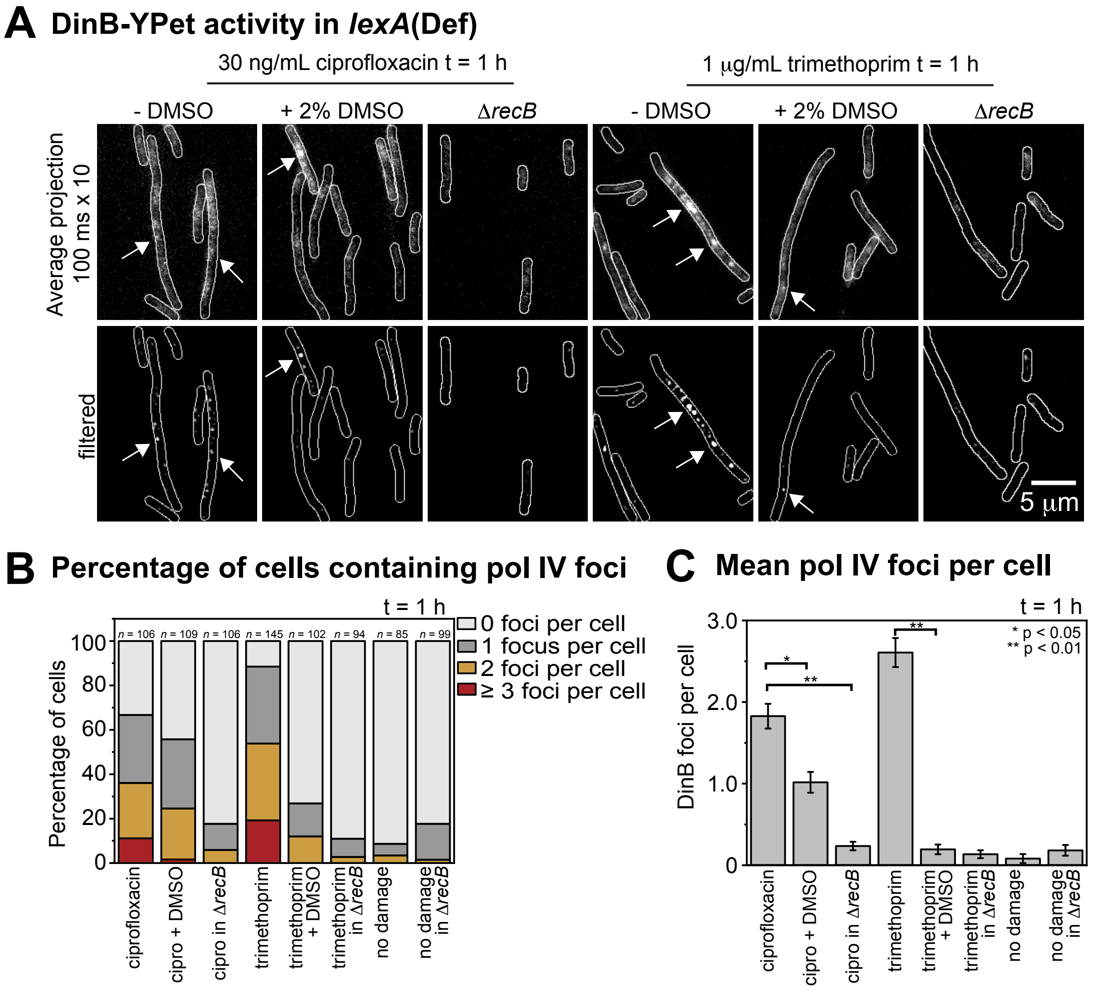

Supplementary Figure S16

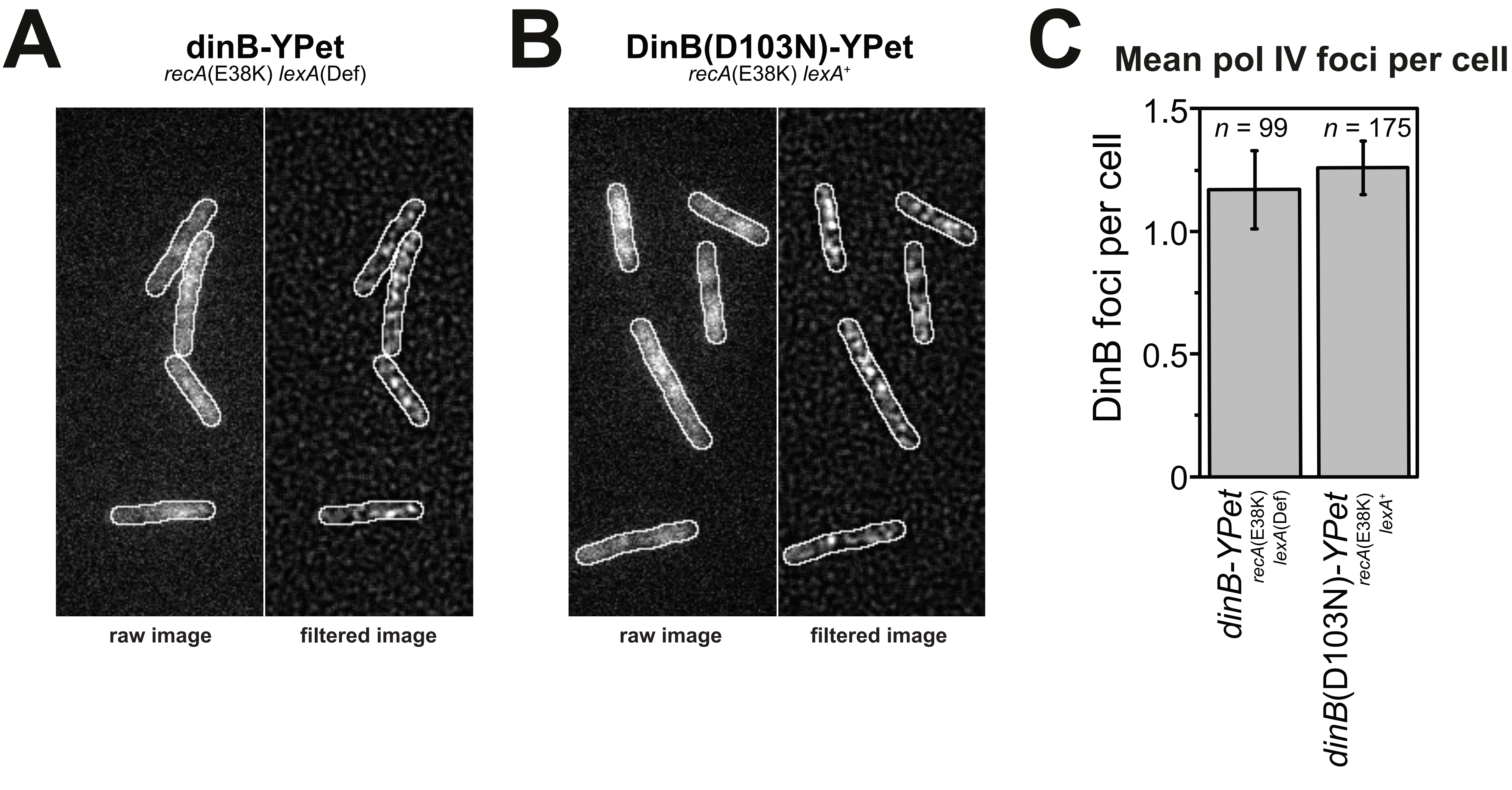

Supplementary Figure S17

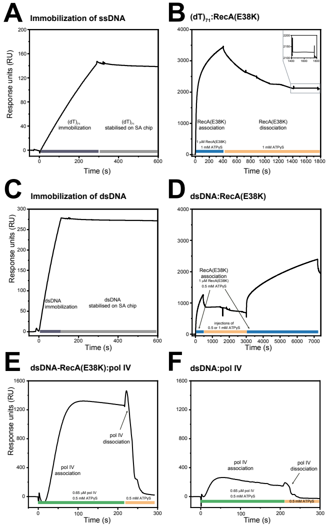

Supplementary Figure S18

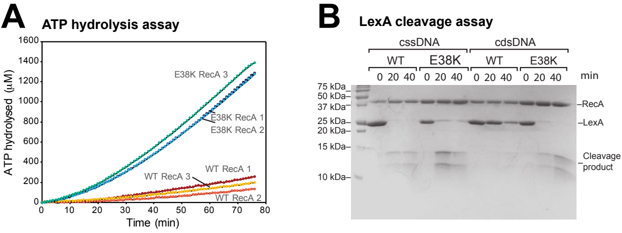

Supplementary Figure S19

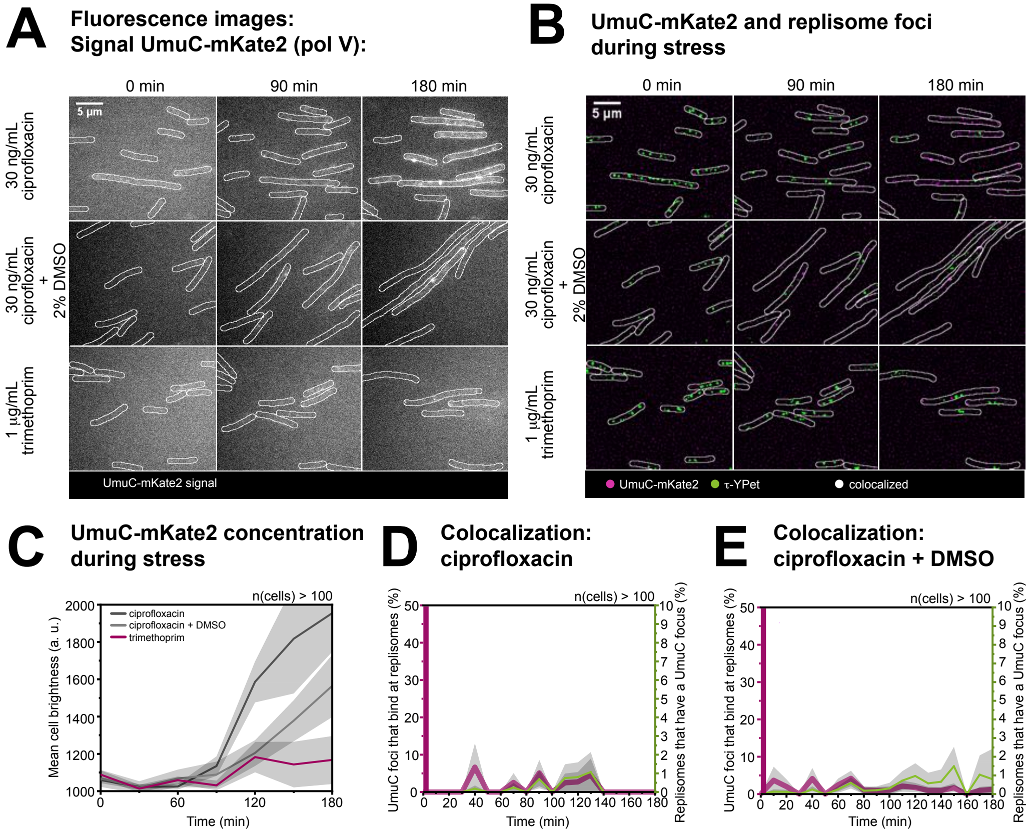

Supplementary Figure S20

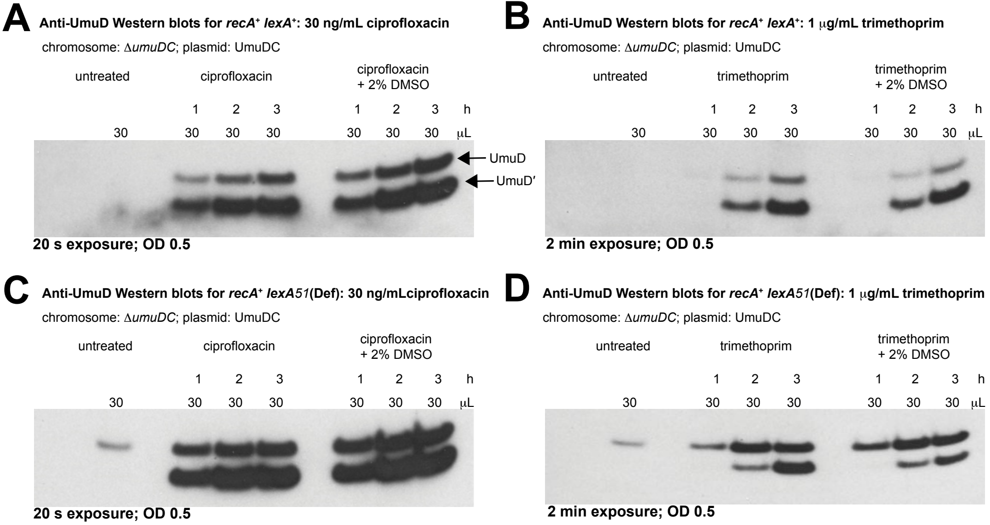

Supplement: gkaa597_Supplemental_Files [file gkaa597_supplemental_files.zip › SupplementaryMaterials.pdf]
